# Effects of sport specific unplanned movements on ankle kinetics and kinematics in healthy athletes from systematic review with meta-analysis

**DOI:** 10.1038/s41598-025-18746-9

**Published:** 2025-09-12

**Authors:** Florian Giesche, Felix Stief, David A. Groneberg, Jan Wilke

**Affiliations:** 1 Institute of Occupational, Social and Environmental Medicine, Division of Preventive and Sports Medicine, Goethe University Frankfurt, Theodor-Stern-Kai 7, 60590 Frankfurt/Main, Germany; 2 Function and Motion Lab, Berufsgenossenschaftliche Unfallklinik Frankfurt, Frankfurt/Main, Germany; 3 Bayreuth Center of Sport Science, Neuromotorics and Movement, University of Bayreuth, Bayreuth, Germany; 4 Institute of Sport Sciences, Department of Movement Science, University of Klagenfurt, Klagenfurt, Austria

**Keywords:** Anticipation, Cognitive function, Choice-reaction, Agility, Ankle sprain, Risk factors, Preventive medicine

## Abstract

Lower limb injuries are common in team sports, often occurring during athletic tasks with high cognitive demands, such as time-constrained decision-making. This systematic review and meta-analysis used robust variance estimation to compare ankle biomechanics during unplanned (time-constrained) versus pre-planned (no decision-making) tasks. Standardized mean differences (SMD) and 95% confidence intervals were pooled to assess differences in ankle kinematics and kinetics. A literature search was conducted in PubMed, Google Scholar, Cochrane Library, and ScienceDirect (until April 2025). Trials assessing ankle kinematics and/or kinetics during pre-planned and unplanned jump-landing or cutting tasks in healthy athletes were included. The GRADE approach was used to rate the certainty of evidence for each outcome as low, moderate, or high. Fifteen trials (162 men, 152 women) with fair to high methodological quality (modified Downs and Black checklist) were identified. In the sagittal plane, low to moderate certainty evidence indicated higher ankle plantarflexion angles during unplanned movement (SMD = 0.27, p = .017). Subgroup analyses showed this effect was most evident in run-and-cut tasks, at initial ground contact, and among elite athletes (SMD = 0.24 to 0.36, p = .017 to .045). Sensitivity analysis excluding outliers indicated low-certainty evidence of increased ankle dorsiflexion angles during unplanned tasks (SMD = 0.25, p = .044), predominantly during the stance phase. Unplanned sports movements may increase ankle plantar- and dorsiflexion, but their impact on injury risk remains uncertain, as these changes may also reflect performance or protective adaptations. Due to the limited number of studies, low certainty of the available evidence, small effect sizes in the sagittal plane, and limited findings in the frontal and transverse planes, further research is warranted to draw more specific conclusions. PROSPERO registration number: CRD42024598492 (date of registration: 18 October 2024)

**Supplementary Information:**

The online version contains supplementary material available at 10.1038/s41598-025-18746-9.

## Introduction

Jump landings and changes of direction (i.e., cutting) are common in team sports such as basketball, football (soccer), volleyball, and handball. These movement patterns create complex biomechanical demands—including velocity modulations, directional changes, and high impact loadings—and are closely linked to the occurrence of musculoskeletal injuries^1–3^. Ankle sprains are among the most frequent complaints at both collegiate and professional levels^4,5^. Traumas to the lateral ligamentous complex make up 80% to 90% of all ankle injuries^6,7^. While recovery is typically faster than for injuries like anterior cruciate ligament (ACL) ruptures, ankle sprains can still cause significant pain, swelling, feeling of giving way, or weakness^8^. With up to 70% of individuals experiencing recurrent lateral ankle sprains following the initial injury^9^, the risk of chronic instability^10^ and early-onset osteoarthritis^11,12 is increased^. Depending on the sport, approximately 20% to 48% of lateral ankle sprains result from non-contact mechanisms (e.g., failed landings or abrupt changes of direction) or indirect contact (e.g., blows to areas like the trunk or shoulder rather than the lower leg)^2,13,14^.

Team and interceptive sports are characterized by high cognitive demands. Athletes must perceive and process multiple visual stimuli (e.g.,  position and movement of the ball, teammates, and opponents) while continuously adapting their motor plans and actions (e.g., changes of direction, landings, and cutting maneuvers) within a highly variable and dynamic environment^15^. Both, non-contact and indirect contact injuries often occur during cognitively challenging game situations^16–19^. To mimic these complex motor-cognitive demands, previous research has employed unplanned movement tasks that include a sudden decision-making component^20^. In these tasks, participants perform athletic movements like jump landings or runs with subsequent cutting maneuvers, that need to be executed in response to spontaneous visual cues (e.g. arrow or light stimulus). During unplanned conditions, the visual stimulus indicating the requested landing side or cutting direction is presented only during the movement (about 400 to 600 ms before ground contact). In contrast, pre-planned tasks provide the visual cue before movement initiation, allowing for sufficient feedforward planning and mental preparation. Systematic reviews indicate that unplanned movement tasks evoke unfavourable knee biomechanics (e.g. reduced flexion angles, increased knee abduction or internal tibia rotation moments) potentially exposing the knee to higher loads and higher risks of injury (e.g. anterior cruciate ligament tears, medial collateral ligament or medial meniscus lesions)^20–24^. The lower limbs work as an integrated entity. Therefore, changes in the movement pattern or loads that occur in one joint lead to inevitable alterations in neighbouring joints^25^. While unplanned movement tasks’ effects on knee biomechanics have been widely examined, systematic evidence on ankle biomechanics is scarce. If unplanned movements also affect the ankle, it would support previous recommendations to include cognitive challenges (e.g. unplanned conditions) in training and testing for injury prevention and risk screening in sport^26^. Such approach could help identify athletes at risk who might remain undetected under conventional pre-planned testing conditions, which does not account for the high perceptual-cognitive demands experienced during real-game situations. The potential relevance of these biomechanical changes in relation to injury risk is well-established. For example, landing with a plantarflexed ankle might increase the risk of ankle sprain. However, altered kinematics in the sagittal plane may also modify performance. Some research argued that landing at increased plantar flexion would facilitate power generation during forward propulsion^27^. On the other hand, dorsiflexion rather than plantarflexion increases Achilles tendon stiffness, allowing for greater elastic energy storage and more explosive force production during rapid stretch–shortening cycles, which is particularly relevant during powerful movements such as running and jumping^28^.

Against this background, the objective of the present systematic review with meta-analysis was (1) to summarize the evidence regarding the effects of unplanned versus pre-planned movement tasks on ankle biomechanics and (2) to identify relevant effect modifiers in uninjured athletes.

## Methods

A systematic review with meta-analysis was performed adhering to the updated PRISMA (Preferred Reporting Items for Systematic Reviews and Meta-Analyses) guideline^29^. The study followed the recommendations for ethical publishing of systematic reviews proposed by Wager and Wiffen^30^ and was registered in the PROSPERO database (CRD42024598492). No changes were made to the methods, inclusion criteria, or other key aspects of the review compared to the original PROSPERO registration, which was published on October 18, 2024. The review was conducted fully in accordance with the registered protocol. Beyond this, no review protocol was published. The used methodology is based on a previous similar review by our working group^22^. All authors declare no conflicts of interest, as documented in the completed ICMJE disclosure forms^31^ available in the supplementary material(Supplementary Information 1)

### Searches

Two independent investigators (FG, JW) conducted a systematic literature search of articles published from inception until mid-April 2025. Relevant articles were identified using MEDLINE (PubMed), ScienceDirect, Cochrane Library and Google Scholar*.* The search terms (Supplementary Information 2) were consistent across all databases, with only minor modifications for Science Direct to meet its specific search requirements (e.g., a maximum of 8 Boolean connectors per query). Google Scholar was used as an additional source to supplement the main search using the above databases. Following the recommendations of Haddaway et al. (2015)^32^, the search was extended to include the first 300 results to ensure a broader capture of relevant studies. This expanded search strategy is widely accepted in comparable systematic reviews^22,33^ and was deemed appropriate for the objectives of the present study. Furthermore, the reference lists of all included studies were screened to identify any additional eligible articles.

### Study inclusion and exclusion criteria

Controlled cross-sectional trials as well as controlled intervention trials (extraction of baseline data only) examining ankle kinematics and/or kinetics during pre-planned (control condition) and unplanned landings/cuttings were considered eligible. Planned movement was defined as any task where the required landing leg/cutting direction was already known prior to movement initiation (e.g., before jumping/starting to run). Unplanned movement was defined as any task where this information was presented spontaneously during movement (e.g., shortly prior to ground contact in jump landings or during running, immediately preceding a change of direction/cutting movement). Besides assessment of both, planned and unplanned movement, further inclusion criteria were (1) enrolment of uninjured individuals (i.e., no history of knee or ankle surgery, no lower limb injury within the last 6 months), and (2) publication in English or German language (latter is the native language of the authors) in a peer-reviewed journal. Uncontrolled studies examining pre-planned or unplanned tasks only were not included. If a study included an uninjured and an injured group (e.g., history of ankle sprain or chronic ankle instability), only the data of the uninjured group were extracted. Table 1 summarizes the inclusion and exclusion criteria in detail, following the PICO framework. Additionally, Supplementary Information 2 provides a detailed overview of the individual exclusion criteria as applied during the literature search.

**Table 1:** Inclusion and Exclusion Criteria Based on the PICO Framework.

PICO	Description
Population	Uninjured individuals (no history of knee or ankle surgery, no lower limb injury within the last 6 months)
Intervention	Ankle kinematics and/or kinetics assessment during pre-planned (control condition) and unplanned landings/cuttings
Comparison	Pre-planned (control) vs. unplanned movement
Outcome	Differences in ankle kinematics and/or kinetics between pre-planned and unplanned landings/cuttings
Additional Inclusion Criteria	1. Published in English or German language in a peer-reviewed journal 2. Controlled (pre-planned task) cross-sectional or controlled intervention trials (baseline data only) 3. Exclusion of uncontrolled studies examining pre-planned or unplanned tasks only 4. If study includes both uninjured and injured groups, only data from the uninjured group were included

### Study quality assessment

We used an adapted version of the Downs and Black checklist^34^ to assess the methodological quality of the included studies like in a similar previous systematic review^22,33^. The modified version included 18 items, grouped into the four categories: reporting quality (9 items), external validity (2 items), internal validity (6 items), and power (1 item). For each criterion met, 1 point was awarded, and a sum score (maximum 18 points) was calculated. The following cutoff points were used to rate the overall study quality: excellent (17–18 points), high (13–16 points), fair (10–12 points) and poor (< 10 points). For details of rating criteria, refer to Table 2). Two investigators (FG, JW) independently performed all ratings, and any disagreements were resolved through discussion, with consensus reached in all cases without consulting the third investigator (FS). Inter-rater reliability for the study quality ratings was assessed using the Intraclass Correlation Coefficient (ICC), which was calculated to evaluate the consistency between the two raters. Publication bias was assessed through visual inspection of funnel plots^35^. In case of a suspected bias, we additionally considered Egger’s regression tests to screen for funnel plot asymmetry as proposed by Peters et al.^36^. However, in accordance with the Cochrane Handbook for Systematic Reviews of Interventions (version 6.5, Chapter 10.4.3.1), these assessments were only conducted when at least 10 studies were available per outcome measure.

**Table 2:** Adapted version of the Downs and Black checklist – used rating criteria.

	Points were awarded, if the following criteria were clearly met:
**Items**	
**Reporting**	
Aim	The objective of the study is clearly described
Outcomes	Outcome measures are stated in the Introduction or Methods section. Reliability/validity data are provided. Scored “0” if methods are first mentioned in the Results section
Sample characteristics	Characteristics of the included participants (e.g., age, sex, body weight/height, sports and performance level) described. Inclusion and exclusion criteria should be stated
Motor task/conditions	Adequate and comprehensible reporting of the procedure of the motor task, including the pre-planned and unplanned condition
Confounders	Potential confounders (i.e. assessment of dominant/non-dominant limb, sex, available response time to react to the visual cue; approach speed) are reported
Findings	Adequate and comprehensible reporting of the study findings. All tests mentioned in the Methods section are addressed
Variability estimates	Standard deviations, standard errors or confidence intervals reported. For non-normally distributed data, the interquartile range is reported
Actual p-values	Actual p-values reported instead of the mere reporting of thresholds (e.g., p < 0.05)
Funding	External funding/grants reported
**External validity**	
Participants representative	The study identified the source and target population and provided su fficient details about related characteristics (e.g., sex, age, activity/performance level or playing position), and these participants were actually included. For example, scored “0” if only males/females were included and this was not mentioned in the objectives, or if elite athletes were included and the objective was not specific for this
Setting representative	The athletic tasks consisted of movements performed in the sports habitually performed by the participants (e.g., cutting/jump landing in team sports) and contained a clear decision-making component
**Internal validity (study bias and confounding)**	
Data dredging	If data analysis was consistent with the pre-registration (if available), or described in the Introduction or Methods section, indicating no data dredging, a score of 1 was given
Adequate statistics	Adequate inference statistical analyses were applied to answer the research question. Alpha error inflation is controlled for (statistical power is rated as a separate item)
Multiple testing of the same outcome measure*	If one study tested/investigated the same outcome multiple (> 2x) times (e.g. using different tasks, cutting angles, response times, limb, phases or groups)
Accurate measures	Objective measurement tools with suffi cient test quality (reliability/validity) were used
Randomness of conditions/directions	The order of the planned and unplanned conditions and landing side/cutting direction was randomized
Adjustment for confounders	Potential confounders were considered as covariates in the statistical analysis, or, for example, it was made clear that potential confounding variables (e.g., approach speed and available response time) did not differ between conditions
**Statistical power**	An a priori sample size calculation was performed and detailed in the Methods section and/or if statistically significant (p < 0.05) differences between conditions were found for at least one biomechanical outcome

### Assessment of the certainty of evidence

The GRADE (Grading of Recommendations Assessment, Development and Evaluation) approach was used to classify the certainty about the evidence for each outcome measure^37^ as very low, low, moderate, or high. Briefly, classification began with the assumption of high certainty if a randomized trial design without important limitations was used. However, this was not the case, as most included studies used cross-sectional designs, which was considered appropriate for answering the research question, but not sufficient for high certainty according to GRADE. Certainty, was downgraded by one or two levels in case of (very) serious concerns regarding limitations in study design or execution (risk of bias), inconsistency of results, indirectness of evidence, imprecision, and publication bias. On the contrary, presence of a large effect magnitude, evidence of a dose–response gradient (not applicable for the present review), and plausible confounding (controlling for confounding) resulted in an upgrade by one or two levels. Classifications were performed by two independent investigators (FG, JW). A breakdown of all GRADE judgment ratings, including downgrading and upgrading decisions across kinematic and kinetic outcomes, is provided in Supplementary Information 3.

### Data extraction strategy

Two authors (FG and JW) independently extracted the following data: sample size, participant characteristics, movement tasks, measured parameters, and results (i.e., between-condition differences and standard deviations for both conditions). Biomechanical data were cross-verified by a third author (FS). The outcomes of the meta-analyses were ankle angles (kinematics) as well as ankle moments (kinetics) in the frontal (inversion/supination or eversion/pronation), sagittal (plantarflexion/dorsiflexion) and transverse (external/internal rotation or abduction/adduction) plane. In the present review, joint moments were expressed as external moments (e.g. internal plantarflexion moment changed to external dorsiflexion moment). If a study assessed more than one biomechanical outcome, all effect sizes were extracted.

### Data synthesis and presentation

To compute effect sizes, the means and standard deviations (SDs) of the two conditions (pre-planned/unplanned) as well as the SD of the difference between both were extracted or computed. If reporting was incomplete, the corresponding authors of the trials were contacted to request the missing information. If the SD of the difference was not reported or could not be obtained from the study authors, it was imputed according to the recommendations of the Cochrane handbook, using the formula: SD difference = √(SD2 pre-planned + SD2 unplanned) – (2 × correlation coefficient × SD pre-planned × SD unplanned (correlation coefficient was 0.5, which represents a conservative estimate of the correlation between both means` SDs). If the standard deviations of the means for the two conditions were not reported, the values (mean, standard deviation, or confidence intervals) were estimated from figures, following the recommendations of the Cochrane Handbook. The WebPlotDigitizer tool (https://automeris.io/WebPlotDigitizer) was used for data extraction.

Meta-analyses with robust variance estimation were used to pool the standardized mean differences (SMD) and 95% confidence intervals (CI) of ankle kinematics and kinetics between the pre-planned and unplanned conditions. This approach accounts for the dependency of multiple effect sizes within the same study (e.g., ankle plantarflexion in different landing/standing phases, movement tasks or stimulus conditions, or for males and females or dominant and non-dominant limb) in some of the included studies^27^, considering both sampling variance and within- and between-study variance^28^. The meta-analytical results were presented visually by forest plots. Meta-analytical calculations were performed if data from at least five studies were available for each outcome. To obtain the within-study variance (more than one dependent effect size), we used ω^2^, which is effective for estimating heterogeneity when studies report multiple effect sizes for the same outcome under different conditions (e.g., landing/standing phases, sex, dominant leg)^38^. Between-study heterogeneity was assessed using I^2^ statistics and classified as low (< 40%), moderate (30 to 60%), substantial (50 to 90%), or considerable (75 to 100%)^39^. To classify the magnitude of effect sizes, we applied the thresholds suggested by Hopkins et al. (2009)^40^, which are more appropriate for athletes and sports-related outcomes. According to Hopkins’ classification, effect sizes are interpreted as follows: small (0.20), moderate (0.60), large (1.20), very large (2.0), and extremely large (4.0). Publication bias was assessed through visual inspection of funnel plots as indicated above.

### Potential effect modifiers and reasons for heterogeneity

Subgroup analyses were performed to identify potential sources of heterogeneity if the following criteria were fulfilled. Firstly, at least two levels needed to be distinguishable for each moderator (e.g. for sex: female or male). Secondly, for each moderator level (categorical variable), effect sizes from at least four studies were required^41^. Finally, there had to be a reasonable theory of how the moderator could affect biomechanical differences between conditions. A variety of participant-related (e.g. female vs. male^42^, elite vs. non-elite performance level^22,43^) and design-related factors (e.g. running vs. jump landing tasks, initial contact vs. weight acceptance phase, reacting to ≤ 2 vs. > 2 visual stimuli during unplanned trials, available time to respond to the unplanned stimuli: ≤ vs. > median of 400 ms^44^) were considered. The selection of the potential moderators was based on a previous similar review^22^ and its characteristics for each of the included study are shown in Table 3. The subgroup analyses were conducted only on parameters that showed significant differences between the unplanned and pre-planned conditions. Potential effect modifiers were detected by (1) estimating the significance of each moderator level (e.g., only females) using the 95% CI, and (2) testing whether the between-condition differences significantly differed between the respective levels (e.g., females vs. males) ^45^.

**Table 3:** Characteristics of the studies included into qualitative and quantitative synthesis.

Study	Participants	Motor Task	Conditions	Outcomes
Borotikar et al. 2008^49^	N = 24 females (21 ± 3 years; 176 ± 5 cm; 68 ± 7 kg); NCAA Division 1 (football/soccer, basketball, volleyball)**	**Single-leg landing and sidestep/jump** (both limbs assessed and single-leg landings analysed) - land on left foot, immediately and aggressively jump laterally to the right (jumping/cutting angle unclear) - jump off the right foot, laterally to the left - land on both feet and jump vertically	**Visual (light) stimulus** UP: following take-off (350 ms prior to ground contact) PP: before initiation of the jump (3 stimuli indicating motor task)	Initial contact *** 3D ankle angles (sagittal plane) Maximum during (mid-)stance phase (approx. at 50%): *** 3D ankle angles (frontal plane)
Meinerz et al. 2015^50^	N = 18 females (20 ± 1 yrs.; 167 ± 6 cm; 66 ± 2 kg); NCAA Division (football/soccer) **	**Single-leg landing and sidestep/cut** (dominant limb and sidecut analyzed) standing on a box and perform a single-leg forward stride to land on the dominant leg and then: - running straight ahead - stop and balance on the leg - sidecut away from landing leg (cutting angle unclear)	**Visual (light) stimulus** UP: stimulus, when the participant fully left the contact mat (available response time unclear) PP: prior to trial-initiation (3 stimuli indicating motor task)	Initial contact: *** 3D ankle angles (sagittal, frontal and transverse plane) From initial contact to max. knee or hip flexion: *** 3D ankle moments (sagittal, frontal, transverse plane)
Mache et al. 2013^56^	N = 13 females (22 ± 1 yrs.; 172 ± 0.1 cm; 61 ± 6 kg) and N = 16 men (22 ± 3 yrs.; 180 ± 0.1 cm; 79 ± 10 kg); recreational athletes	**Bilateral landing with/without vertical jump** (dominant limb assessed) Participants began each trial suspended in the air, hanging by the hands from a horizontal overhead bar (hanging ~ 20% of measured body height above force plates): - drop-jump landings (maintain stance/balance upon landing) - drop-jumps (jump up immediately upon landing and attempt to touch the bar)	**Visual (light) stimulus** UP: stimulus, when the participant released the bar (~ 250 ms before ground contact) PP: prior to releasing the bar (2 stimuli indicating motor task)	Initial contact and from initial contact to max. knee or hip flexion: *** 3D ankle angles (sagittal and frontal plane) From initial contact to max. knee or hip flexion: *** 3D ankle moments (sagittal and frontal frontal)
Yom et al. 2018^57^	N = 18 females (21 ± 2 yrs.; 169 ± 4 cm; 63 ± 4 kg); recreational athletes	**Bilateral landing and sidestep/cut** (dominant limb assessed) Participants hung from a suspended drop bar at a normalised 0.5 m drop height above force plates: - land on both feet and sidecut to the left (45° angle) - land on both feet and sidecut to the right (45° angle)	**Visual (arrow) stimulus** UP: stimulus, when the participant released the bar (~ 320 ms before ground contact) PP: prior to releasing the bar (2 stimuli indicating motor task)	Initial contact *** 3D ankle angles (frontal plane) From initial contact to max. knee or hip flexion: *** 3D ankle angles (frontal plane)
Weinhandl et al. 2013^58^	N = 20 females (21 ± 1 yrs.; 166 ± 5 cm; 62 ± 6 kg); recreational athletes (e.g. soccer, volleyball, tennis, basketball	**Run and sidestep/cut** (right limb assessed and sidecut analysed) - run and straight run ahead - run and sidestep to the left (45°) - run and stop quickly (approach speed: 4.5 to 5 m/s)	**Visual (light) stimulus** UP: 600 ms before reaching the force plate PP: before the initiation of the run (3 stimuli indicating motor task)	Weight acceptance/loading response phase (at peak ACL loading): *** - 3D ankle angles (sagittal plane) - 3D ankle moments (sagittal plane)
Stoffel et al. 2010^51^	N = 22 males (22 ± 2 yrs.; 185 ± 0.1 cm; 83 ± 7 kg); Semi-professional or elite Australian Rules Football players **	**Run and sidestep/cut and straight run** (dominant limb, straight run and sidestep/cut analysed) - run and straight run - run and sidestep (45°) - run and crossover cut (45°) (approach speed: 5.5 ± 0.5 m/s)	**Visual (light) stimulus** UP: 400 ms before reaching the force plate PP: at the commencement of the run (3 stimuli indicating motor task)	Weight acceptance/loading response phase (from heel strike to first trough in V-GRF): *** - 3D ankle angles (sagittal, frontal and transverse plane) - 3D ankle moments (sagittal, frontal and transverse plane)
Whyte et al. (B)^55^	N = 28 males (21.7 ± 2 yrs.; 179 ± 15 m; 82 ± 11 kg) *; University Gaelic Football	**Single-leg landing and sidestep/cut** (dominant limb and sidecut analyzed) Horizontal jump equaling 70% of the maximal jump distance and then: - crossover cut (45°) - sidecut (45°) - stop jump	**Visual (light) stimulus** UP: 150 ms prior to initial contact PP: three seconds before the initiation of the task (3 stimuli indicating motor task)	Weight acceptance/loading response phase (from heel strike to first trough in V-GRF): *** - 3D ankle angles (sagittal plane) - 3D ankle moments (sagittal and transverse plane)
Whyte et al. (A)^54^	N = 28 males (21.7 ± 2 yrs.; 179 ± 15 m; 82 ± 11 kg) *; University Gaelic Football	**Single-leg landing and crossover cut** (dominant limb and crosscut analyzed) Horizontal jump equaling 70% of the maximal jump distance and then: - crossover cut (45°) - sidecut (45°) - stop jump	**Visual (light) stimulus** UP: 150 ms prior to initial contact PP: three seconds before the initiation of the task (3 stimuli indicating motor task)	Weight acceptance/loading response phase (from heel strike to first trough in V-GRF): *** - 3D ankle angles (sagittal, frontal and transverse plane) - 3D ankle moments (sagittal and frontal plane)
Richwalski et al. 2019^53^	N = 12 females (20 ± 1 yrs.; 170 cm; 71 ± 10 kg); Volleyball at collegiate club-level	**Bilateral landing and crossover cut or vertical jump** (dominant limb assessed) Drop from a 40 cm box and land on both feet and then: - crossover step left - crossover step right - vertical jump	**Visual (arrow) stimulus** UP: while stepping of the box (available response time unclear) PP: prior to leaving the box (3 stimuli indicating motor task)	Initial contact to max. knee flexion: *** 3D ankle moments (sagittal, frontal and transverse plane)
Kim et al. 2014^48^	N = 37 young, male middle school football/soccer players (expertise level, exact age, and anthropometrics unclear)	**Run and side/crossover step/cut** (dominant limb assessed) - run and sidecut (45°) - run and crossover cut (45°) (approach speed: 3.5 ± 0.2 m/s)	**Visual (light) stimulus** UP: 90% of stride length before the force plate (available response time not reported, approx. 250 to 350 ms) PP: before the initiation of the run (2 stimuli indicating motor task)	Maximum during (mid-)stance phase: *** - 3D ankle angles (sagittal, frontal and transverse plane) - 3D ankle moments (sagittal, frontal and transverse plane)
Kim et al. 2016^47^	N = 16 young, male middle school football/soccer players (expertise level unclear; 165 ± 9 cm; 55 ± 10 kg; exact age unclear)	**Run and side step/cut** (dominant limb assessed) - run and sidecut left (45°) - run and sidecut right (45°) (approach speed 3.5 ± 0.2 m/s)	**Visual (light) stimulus** UP: 90% of stride length before the force plate (available response time not reported, approx. 250 to 350 ms) PP: before the initiation of the run (2 stimuli indicating motor task)	Weight acceptance/loading response phase (approx. at 8%) and max. during (mid-)stance phase (approx. at 45%): *** - 3D ankle angles (sagittal, frontal and transverse plane) - 3D ankle moments (sagittal, frontal and transverse plane)
Hou et al. 2024^59^	N = 15 physically (recreational) active participants (n = 7 females; age: 24 ± 6 years, BMI: 23 ± 4 kg/m^2^)	**Bilateral countermovement-jump with single-leg landing** (both limbs assessed) - landing on the left side - landing on the right side	**Visual (written word) stimulus** UP: landing side (left or right) automatically shown 100 ms after take-off (available response time: ~ 400 ms) PP: landing side (left or right) shown before jump (2 stimuli indicating motor task)	~ 20 ms prior initial contact *** 3D ankle angles (sagittal plane) Peak within 200 ms upon landing (~ 120 ms after initial contact): *** 3D ankle angles (sagittal and frontal plane)
Zou et al. 2024^52^	N = 21 female college soccer players (national level or above; all had finished top three national competitions), age: 18 ± 1 years; height: 1.66 ± 0.05 m; weight: 60.1 ± 5.5 kg **	**Run and cutting** (dominant limb assessed) - run and sidecut (45°) - run and crosscut (90°) - run and pivot turn (180°) (approach speed: 2.6 ± 0.3 m/s)	**Visual (arrow) stimulus** UP: 2 m before the force plate (available response time not reported, approx. 750 ms) PP: before the initiation of the run (3 stimuli indicating motor task)	Initial contact and first peak vGRF/weight acceptance/loading response phase) *** 3D ankle angles (sagittal, frontal and transverse plane)
Rikken et al. 2024^60^	N = 15 male basketball players (local, regional, national level), age: 22 ± 2 years; height: 1.89 ± 11 m; weight: 85 ± 12 kg	**Run and side step/cut** (dominant limb assessed) - run and sidecut left (90°) - run and sidecut right (90°) (approach speed: unclear)	**Visual (light) stimulus** Each trial involved two sidestep cuts: the first with the non-dominant leg after an 8.35 m sprint (Fitlight#2), and the second—a 90° cut with the dominant leg (Fitlight#3) following a 3.5 m sprint. The trial ended with another 3.5 m sprint (available response time: unclear) UP: route indicated when reaching Fitlight#2 (available response time not reported) PP: route indicated during the entire trial (2 stimuli indicating motor task)	Initial contact*** 3D ankle angles (plantarflexion; sagittal plane) Peak between initial contact and max. knee flexion*** 3D ankle angles (dorsiflexion; sagittal plane) (IMU-based system; Xsens MVN Analyze)
Dutaillis et al. 2021^61^	N = 19 recreationally active females (age, 24 ± 3yrs; height, 164 ± 5 cm; and weight, 58 ± 6 kg), type of sports (e.g. Soccer, Australian Rules football and gymnastics)	**Single-leg landing and cutting** (both limbs assessed) forward jump off a 0.31 m box placed 1.35 m from the centre of the force plate, landing on a single limb and immediately: - sidecut to the left (45°) - sidecut to the right (45°)	**Visual stimulus** UP: 450 ms before initial contact PP: before the initiation of the jump (2 stimuli indicating motor task)	**Peak during stance phase (~ 60–80%)** - 3D ankle angles (dorsiflexion; sagittal plane) - 3D ankle angles (inversion; frontal plane)

* same participants, ** allocated to elite performance level.

*** used definition of stance/landing phase: (1) initial contact, (2) loading response/weight acceptance phase (1 to 20% of stance), (3) midstance/peak push off phase (21 to 60% of stance), and (4) terminal stance/final push off phase (last 15% of stance)^99,100^.

UP = Unplanned, PP = pre-planned, yrs = years, 3D = three-dimensional motion analysis; NCAA = National Collegiate Athletic Association

To quantify the potential influence of outliers on the overall effects of unplanned movement on individual kinematic and kinetic outcomes, sensitivity analyses were conducted. These involved excluding effect sizes whose confidence intervals did not overlap with the pooled effect line in the forest plots. The software used was R (R Foundation for Statistical Computing, Vienna, Austria), packages Meta and Robumeta (version 2.1)^46^. The corresponding analysis code used has been added as Supplementary Information 4.

## Results

### Review statistics

**Fig. 1:**
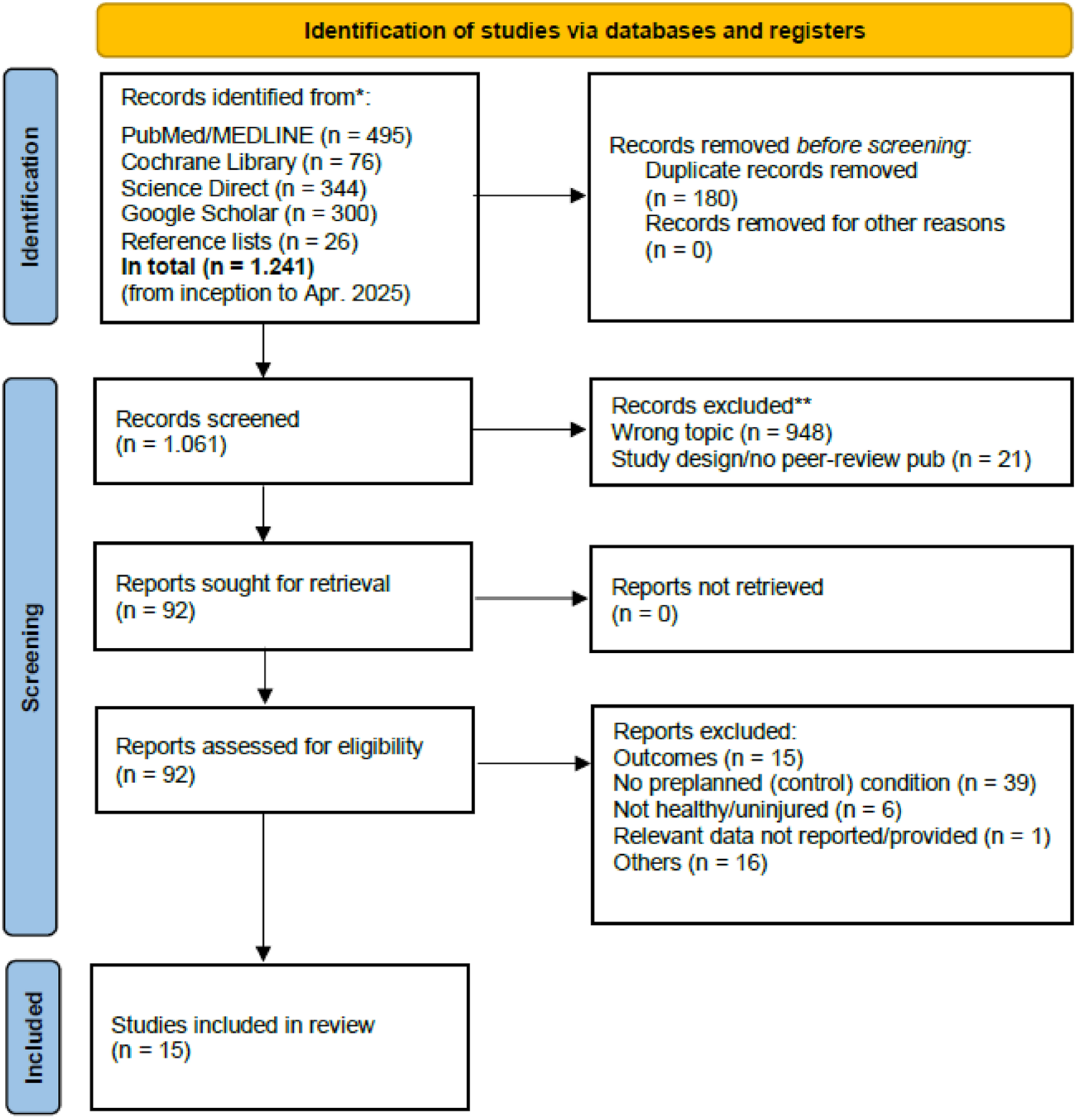
PRISMA Flow Diagram.

The searches returned a total of 1.241 articles (Fig. 1). After duplicate removal and application of exclusion criteria, 15 studies (11 studies identified via PubMed/MEDLINE, 1 via Google Scholar, 1 via Cochrane library, and 2 through manual reference list screening) were included in the quantitative synthesis (all full texts were available). For a list of the excluded studies, refer to Supplementary Information 5.

The 15 papers collectively evaluated 314 participants (162 men and 152 women) with mean age of 21.5 ± 1.6 years and mean BMI of 23.0 ± 1.2 kg/m^2^ (detailed characteristics see Table 3). Two studies included children or adolescents^47,48^. Four studies included elite or semi-professional athletes^49–52^, nine studies included non-elite athletes (e.g., college/university athletes on a lower performance level or recreational athletes^53–61^. Four studies reported external and five studies reported internal joint moments. Fourteen trials^47–52,54–61^ investigated ankle kinematics (14 sagittal, 12 frontal, 6 transverse plane) while 9 studies^47,48,50,51,53–56,58^ examined ankle kinetics (9 sagittal, 7 frontal, 6 transverse plane). In 12 studies, only the dominant limb was analyzed.

Thirteen studies investigated pre-planned and unplanned change of direction tasks involving running or jump landings followed by side or cross steps. Of these, 11 studies employed running or single-leg jump landing tasks^47–52,54,55,58,60,61^, while 2 studies^53,57^ used bilateral landing tasks. Additionally, two studies^56,59^ examined movement conditions without a change of direction component, such as bilateral landings followed by vertical jumps or bilateral jumps with single-leg landings only. Ankle biomechanics were assessed during the initial contact phase by 6 studies^49,50,52,56,57,60^ and the weight acceptance phase by 6 studies^47,51,52,54,55,58^. Four studies^47–49,61^ used the peak values during the entire stance phase and 5 studies^50,53,56,57,60^ used the peak values occurring between initial contact and maximum knee or hip flexion. In one study ankle mechanics were assessed before initial contact^59^. All studies used motion capture systems with reflective markers (e.g., Vicon system), either with or without force plates (e.g., Kistler or AMTI). One study used an inertial measurement unit (IMU)-based system (Xsens) instead^60^. As the same participants were included in two different publications^54,55^, the effect sizes of these two studies were considered as one (Whyte A^54^ + B^55^) to account for dependency of effect sizes.

Because of an insufficient number of studies, meta-analysis was not possible for ankle angles in the transverse plane as well as external ankle plantarflexion, inversion/supination, abduction/adduction and external rotation moments. Evidence for these variables is reported in the qualitative synthesis. All data for the meta-analyses were available in the tables of the original publications, except for four cases. In three of these, the authors^54,55,59^ provided the requested data upon request, while in the remaining case^61^, the relevant values were imputed from figures.

### Study quality assessment

The methodological quality was fair to high (mean: 13.9/18 points), ranging from 12 to 16 points (Table 4). With regard to the sub-scores, the mean values were 7.8 out of 9 points (range: 7 to 9 points) for reporting (very low reporting bias), 1.3 out of 2 points (range: 1 to 2 points) for external validity, and 3.9 out of 6 points (range: 4 to 6 points) for study bias and confounding (for detailed rating criteria, refer to Table 2). All studies had adequate statistical power. Eight studies lacked a clear link between the objective and target population (0 points for participant representativeness). Six studies did not specify marker placement on the foot and ankle or use a biomechanical foot model (0 points for accurate measures). Eleven studies failed to report or adjust for potential differences in available response time (e.g. flight time or approach speed) between pre-planned and unplanned conditions. From these, three trials did not consider limb dominance, and one study did not report sex-specific results (0 points for adjusting potential confounders). Eleven studies measured outcomes multiple times; thus, due to potential inaccuracies and confounding, bias cannot be excluded. The inter-rater reliability of our study quality ratings was high (ICC of 0.82).

**Table 4:** Study quality rating (adapted Down and Black Checklist).

**Item**	**Borotikar**^49^	**Hou**^59^	**Kim A**^48^	**Kim B**^47^	**Mache**^56^	**Meinerz**^50^	**Richwalski**^53^	**Stoffel**^51^	**Weinhandl**^58^	**Whyte A**^54^	**Whyte B**^55^	**Yom **^57^	**Zou **^52^
Aim	1	1	1	1	1	1	1	1	1	1	1	1	1
Outcome	1	1	1	1	1	1	1	1	1	1	1	1	1
Sample	1	1	0	0	1	1	1	1	1	1	1	1	1
Motor tasks/Conditions	1	1	1	1	1	1	1	1	1	1	1	1	1
Confounders	1	1	1	1	1	1	1	1	1	1	1	1	1
Findings	1	0	1	1	1	1	1	1	1	0	0	1	1
Variability estimates	1	0	1	1	1	1	1	1	1	0	0	1	1
Actual p-values	0	1	1	1	1	1	1	1	1	1	1	1	1
Funding	1	1	1	1	0	0	0	1	1	1	1	0	1
**Total reporting**	**8/9**	**7/9**	**8/9**	**8/9**	**8/9**	**8/9**	**8/9**	**9/9**	**9/9**	**7/9**	**7/9**	**8/9**	**9/9**
Participants representative	0	1	0	0	1	0	1	0	0	0	0	1	1
Setting representative	1	1	1	1	0	1	1	1	1	1	1	0	1
**Total external validity**	**1/2**	**2/2**	**1/2**	**1/2**	**1/2**	**1/2**	**2/2**	**1/2**	**1/2**	**1/2**	**1/2**	**1/2**	**2/2**
Data dredging	1	1	1	1	1	1	1	1	1	1	1	1	1
Adequate statistics	1	1	1	1	1	1	1	1	1	1	1	1	1
Multiple testing same outcome	0	0	0	0	0	1	0	0	0	0	0	1	0
Accurate measures	1	1	0	0	1	0	0	1	1	0	0	1	1
Randomization of conditions	1	1	1	1	1	0	1	1	1	1	1	1	1
Adjustment for confounders	0	0	0	0	1	1	1	0	0	0	0	1	0
**Total internal** **validity— study bias and confounding**	**4/6**	**4/6**	**4/6**	**3/6**	**4/6**	**4/6**	**4/6**	**4/6**	**4/6**	**3/6**	**3/6**	**6/6**	**4/6**
Sufficient power	1	1	1	1	1	1	1	1	1	1	1	1	1
**Total power**	**1/1**	**1/1**	**1/1**	**1/1**	**1/1**	**1/1**	**1/1**	**1/1**	**1/1**	**1/1**	**1/1**	**1/1**	**1/1**
**Sum score**	**14/18**	**14/18**	**13/18**	**13/18**	**14/18**	**14/18**	**15/18**	**15/18**	**15/18**	**12/18**	**12/18**	**16/18**	**15/18**

Item	Rikken^60^	Dutailli^61^
Aim	1	1
Outcome	1	1
Sample	1	1
Motor tasks/Conditions	1	1
Confounders	0	1
Findings	1	0
Variability estimates	1	0
Actual p-values	0	0
Funding	1	1
**Total reporting**	**7/9**	**6/9**
Participants representative	1	1
Setting representative	1	1
**Total external validity**	**2/2**	**2/2**
Data dredging	1	1
Adequate statistics	1	1
Multiple testing same outcome	0	0
Accurate measures	1	1
Randomization of conditions	0	1
Adjustment for confounders	0	1
**Total internal** **validity— study bias and confounding**	**3/6**	**5/6**
Sufficient power	1	1
**Total power**	**1/1**	**1/1**

### Quantitative synthesis/Meta-analysis and certainty of evidence

#### Kinematics

**Fig. 2:**
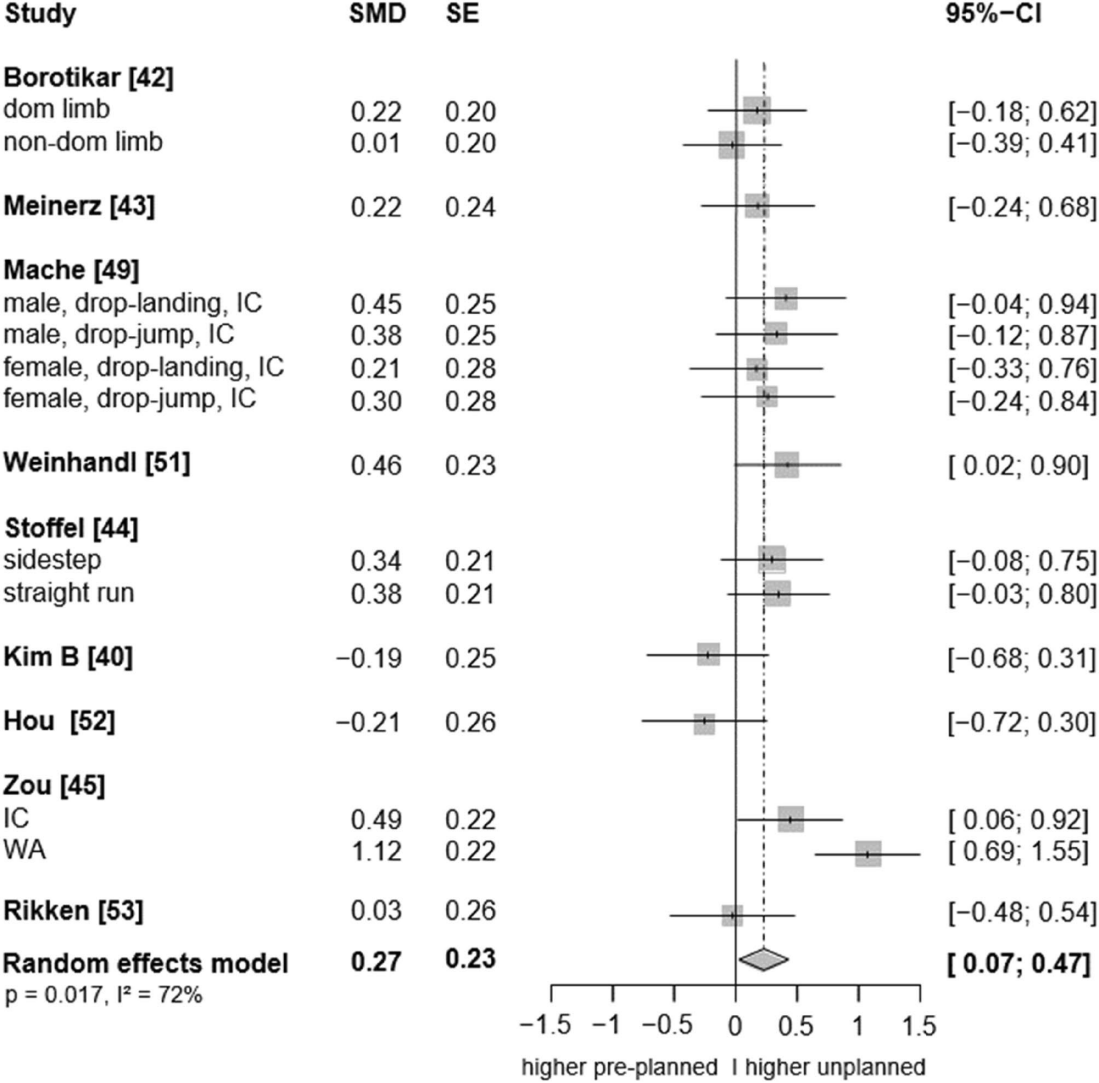
Forest plots of the effects of unplanned athletic movement on ankle plantarflexion angles. Legends: pooled standardized mean differences (SMDs), standard errors (SEs), and 95% confidence intervals (CIs) are displayed. Abbreviations: SMD = Standard mean differences, SE = Standard error are displayed, I^2^ = between-study heterogeneity, IC = initial contact, WA = weight acceptance phase.

**Fig. 3:**
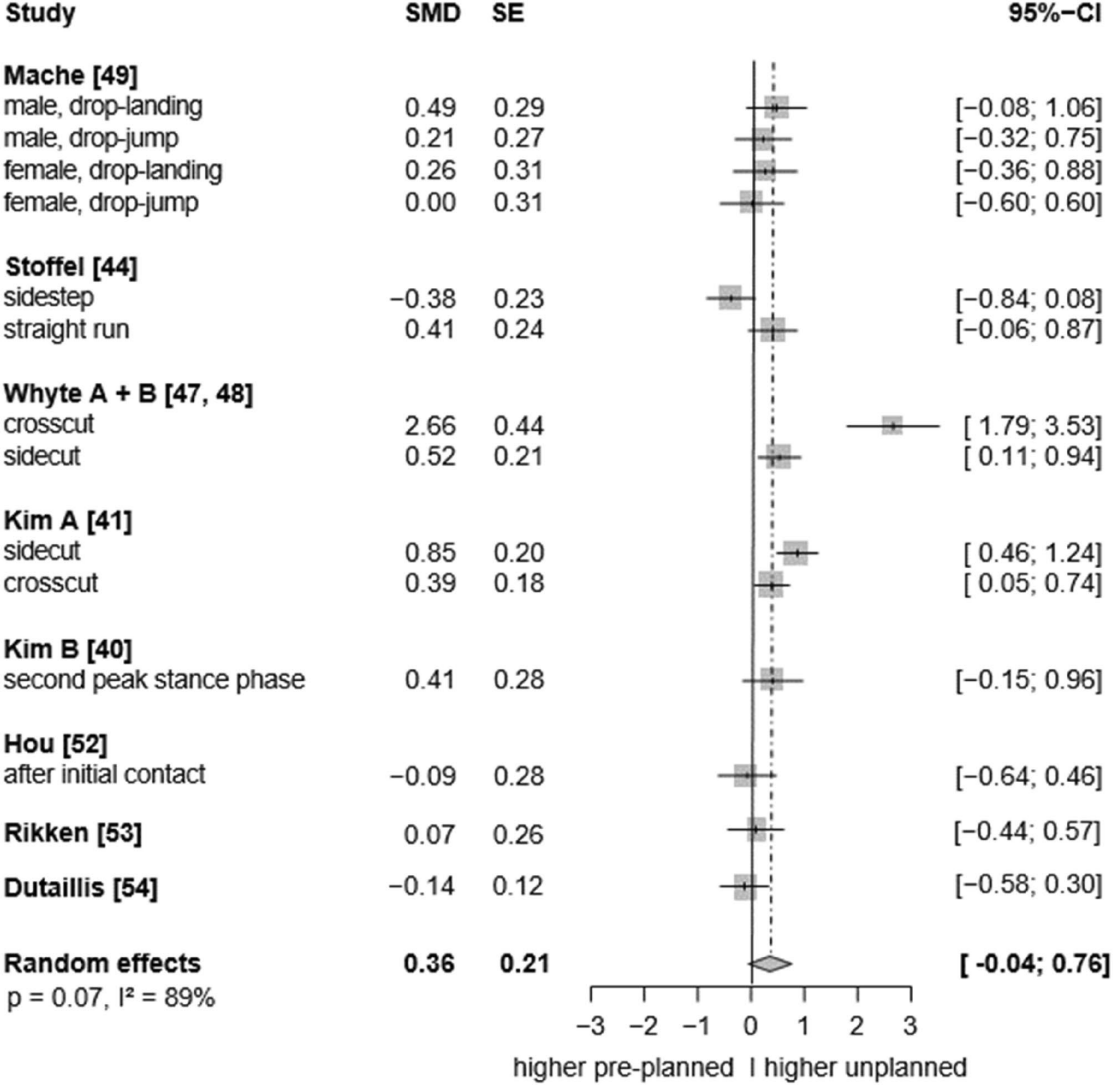
Forest plots of the effects of unplanned athletic movement on ankle dorsiflexion angles. Legends: pooled standardized mean differences (SMDs), standard errors (SEs), and 95% confidence intervals (CIs) are displayed. Abbreviations: SMD = Standard mean differences, SE = Standard error are displayed, I^2^ = between-study heterogeneity.

**Fig. 4:**
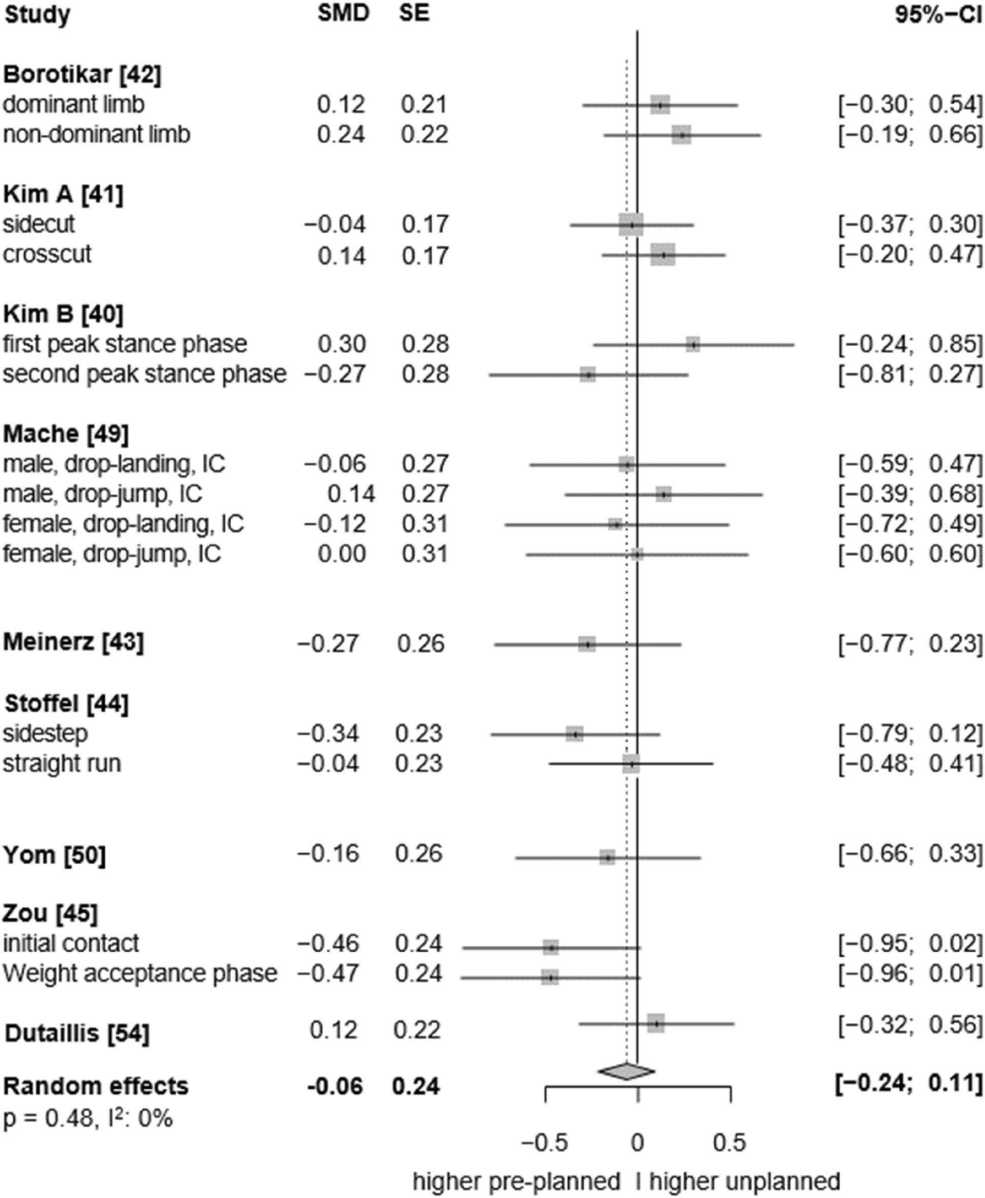
Forest plots of the effects of unplanned athletic movement on ankle inversion angles.Legends: pooled standardized mean differences (SMDs), standard errors (SEs), and 95% confidence intervals (CIs) are displayed. Abbreviations: SMD = Standard mean differences, SE = Standard error are displayed, I^2^ = between-study heterogeneity, IC = initial contact.

**Fig. 5:**
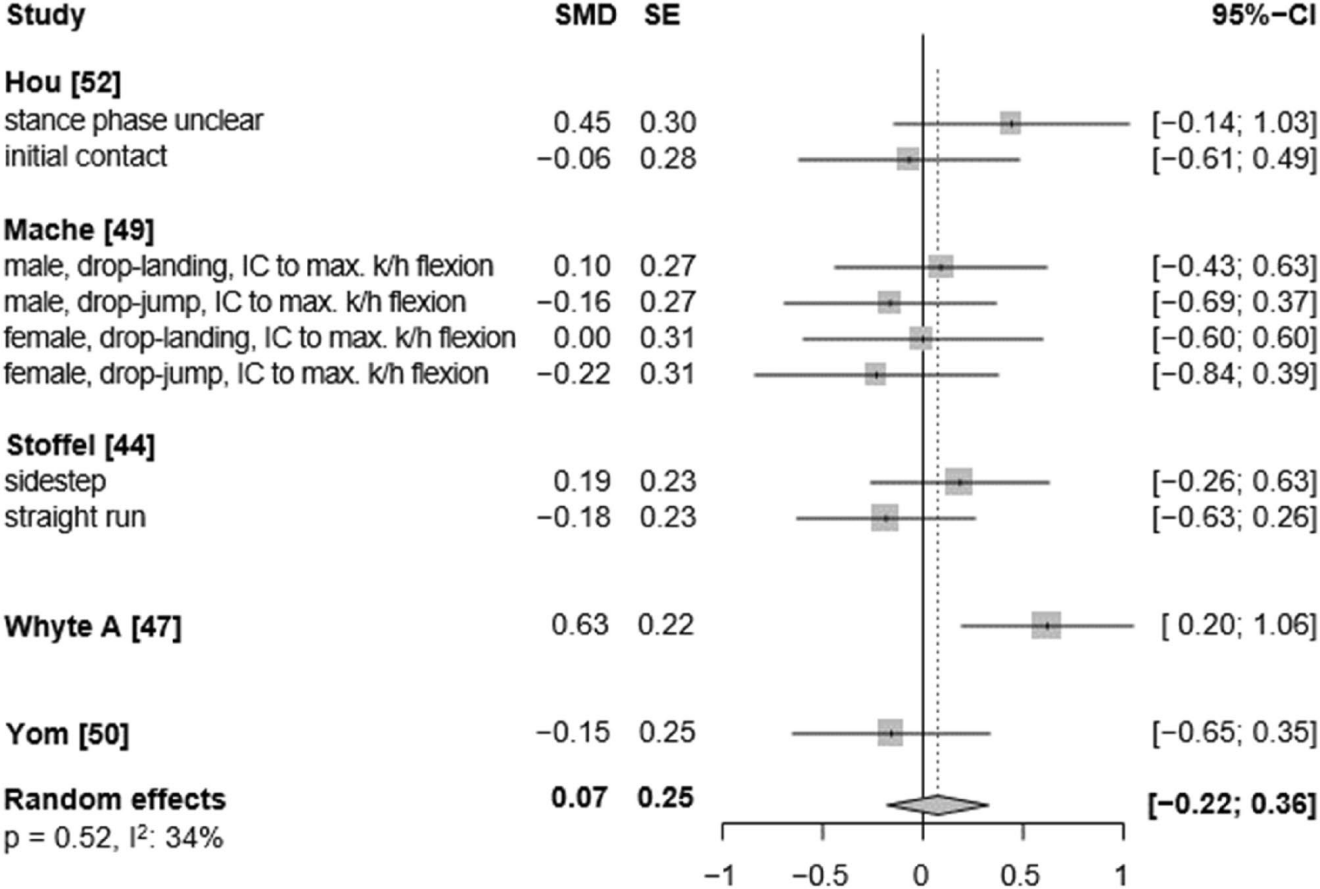
Forest plots of the effects of unplanned athletic movement on ankle eversion angles.Legends: pooled standardized mean differences (SMDs), standard errors (SEs), and 95% confidence intervals (CIs) are displayed. Abbreviations: SMD = Standard mean differences, SE = Standard error are displayed, I^2^ = between-study heterogeneity, IC = initial contact, k/h = knee/hip.

In the sagittal plane, low to moderate certainty evidence indicates that unplanned conditions resulted in higher ankle plantarflexion angles than the pre-planned tasks (SMD: 0.27, 95% CI: 0.07 to 0.47, p = 0.017, ω^2^: 0.00, I^2^: 72%; 9 studies, 15 ES; Fig. 2). Similarly, low-certainty evidence suggests a small effect of unplanned tasks on higher ankle dorsiflexion angles, however, the analysis marginally failed statistical significance (SMD: 0.36, 95% CI: −0.04 to 0.76, p = 0.070, ω^2^: 0.00, I^2^: 89%; 8 studies, 14 ES; Fig. 3). For the frontal plane, there is very low-certainty evidence that unplanned movement has no effect on frontal plane kinematics (inversion angles: SMD: −0.06, 95% CI: −0.24 to 0.11, p = 0.482, ω^2^: 0.00, I^2^: 0%; 9 studies, 17 ES; Fig. 4; eversion angles: SMD: 0.07, 95% CI: −0.22 to 0.36, p = 0.52, ω^2^: 0.02, I^2^: 34%, 5 studies, 10 ES, Fig. 5).

#### Kinetics

**Fig. 6:**
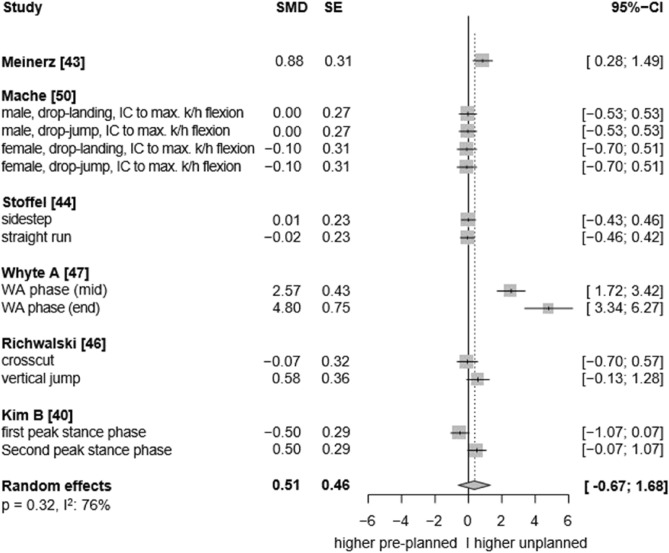
Forest plots of the effects of unplanned athletic movement on ankle eversion moments.Legends: pooled standardized mean differences (SMDs), standard errors (SEs), and 95% confidence intervals (CIs) are displayed. Abbreviations: SMD = Standard mean differences, SE = Standard error are displayed, I^2^ = between-study heterogeneity, IC = initial contact, k/h = knee/hip.

**Fig. 7:**
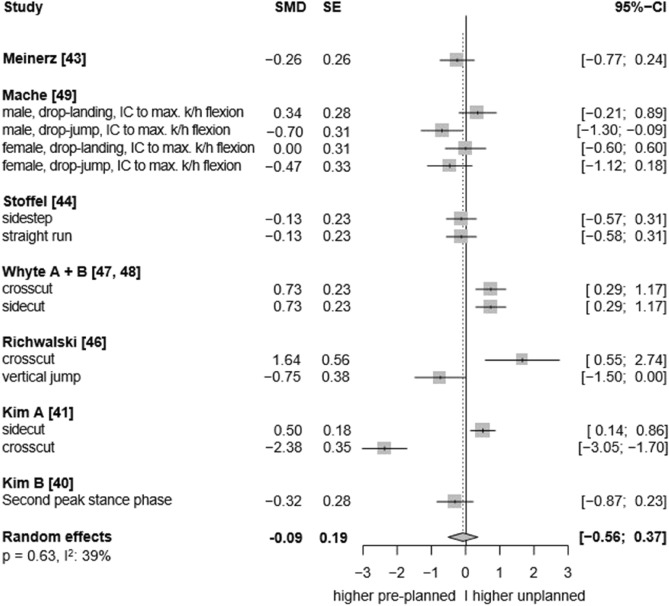
Forest plots of the effects of unplanned athletic movement on ankle dorsiflexion moments.Legends: pooled standardized mean differences (SMDs), standard errors (SEs), and 95% confidence intervals (CIs) are displayed. Abbreviations: SMD = Standard mean differences, SE = Standard error are displayed, I^2^ = between-study heterogeneity, IC = initial contact, k/h = knee/hip.

**Fig. 8:**
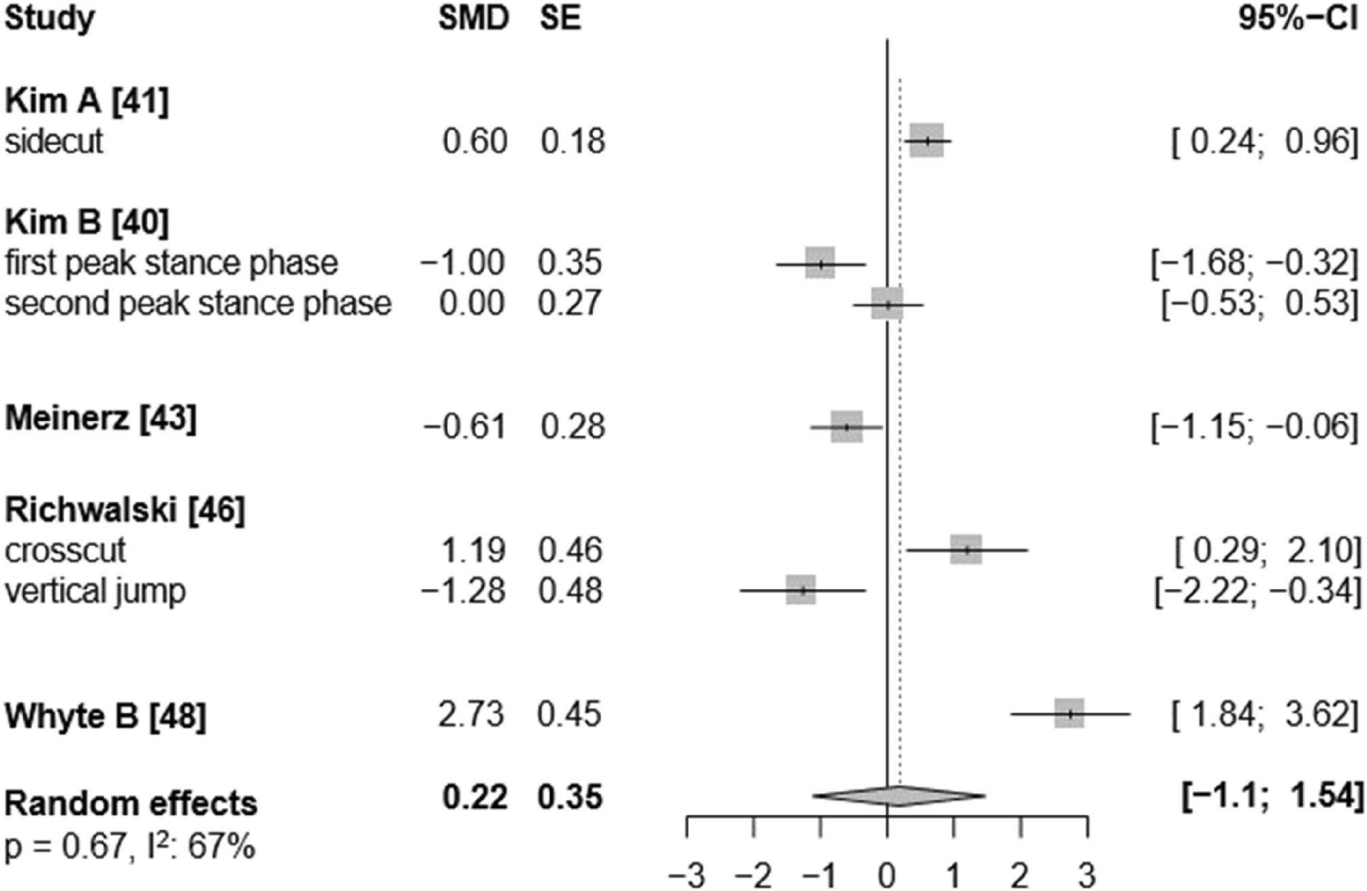
Forest plots of the effects of unplanned athletic movement on ankle internal rotation moments. Legends: pooled standardized mean differences (SMDs), standard errors (SEs), and 95% confidence intervals (CIs) are displayed. Abbreviations: SMD = Standard mean differences, SE = Standard error are displayed, I^2^ = between-study heterogeneity.

In the frontal plane, very low certainty evidence indicates a non-significant effect of unplanned movement on external ankle eversion moments (SMD: 0.51, 95% CI: −0.67 to 1.68, p = 0.32, ω^2^: 0.00, I^2^: 76%; 6 studies, 13 ES; Fig. 6). In the sagittal plane, very low certainty evidence indicates no between-condition effect in terms of the produced external ankle dorsiflexion moments (SMD: −0.09, 95% CI: −0.56 to 0.37, p = 0.64, ω^2^: 0.46, I^2^: 39%; 7 studies, 14 ES; Fig. 7). In the transverse plane, very low-certainty evidence indicates no differences between both conditions regarding external ankle internal rotation moments (SMD: 0.22, 95% CI: −1.1 to 1.54, p = 0.67, ω^2^: 0.63, I^2^: 67%; 5 studies, 7 ES; Fig. 8).

#### Sensitivity analysis

Sensitivity analyses excluding the outlier SMDs from Hou et al. (2024)^59^ (effect size: −0.21), Kim et al. (2016)^47^ (−0.19), and Zou et al. (2024)^52^ (1.12) did not meaningfully alter the results regarding the effect of unplanned movement on ankle plantarflexion angle (SMD = 0.28, 95% CI: 0.14 to 0.43, p = 0.003, ω^2^ = 0.00, I^2^ = 0%; 7 studies, 12 effect sizes). Similarly, excluding the SMD estimated from the figure in the study by Dutaillis et al. (2021)^61^ (–0.14) did not alter the effect of unplanned movement on ankle dorsiflexion angle (SMD = 0.40, 95% CI: –0.04 to 0.84, p = 0.067, ω^2^ = 0.00, I^2^ = 87%). However, excluding the outlier SMD from Whyte et al. (2018)^54^ (2.66)—while retaining the effect size from Dutaillis et al. (2021)^61^—resulted in a statistically significant, but small effect of unplanned movement on ankle dorsiflexion angle (SMD = 0.25, 95% CI: 0.01 to 0.48, p = 0.044, ω^2^ = 0.07, I^2^ = 0%; 8 studies, 13 effect sizes, low certainty of evidence).

For the remaining outcomes, no sensitivity analyses were conducted, as, either no clear outliers were identified or the exclusion of individual outliers would not have influenced the overall effect due to p-values being far from statistical significance.

#### Publication bias

**Fig. 9:**
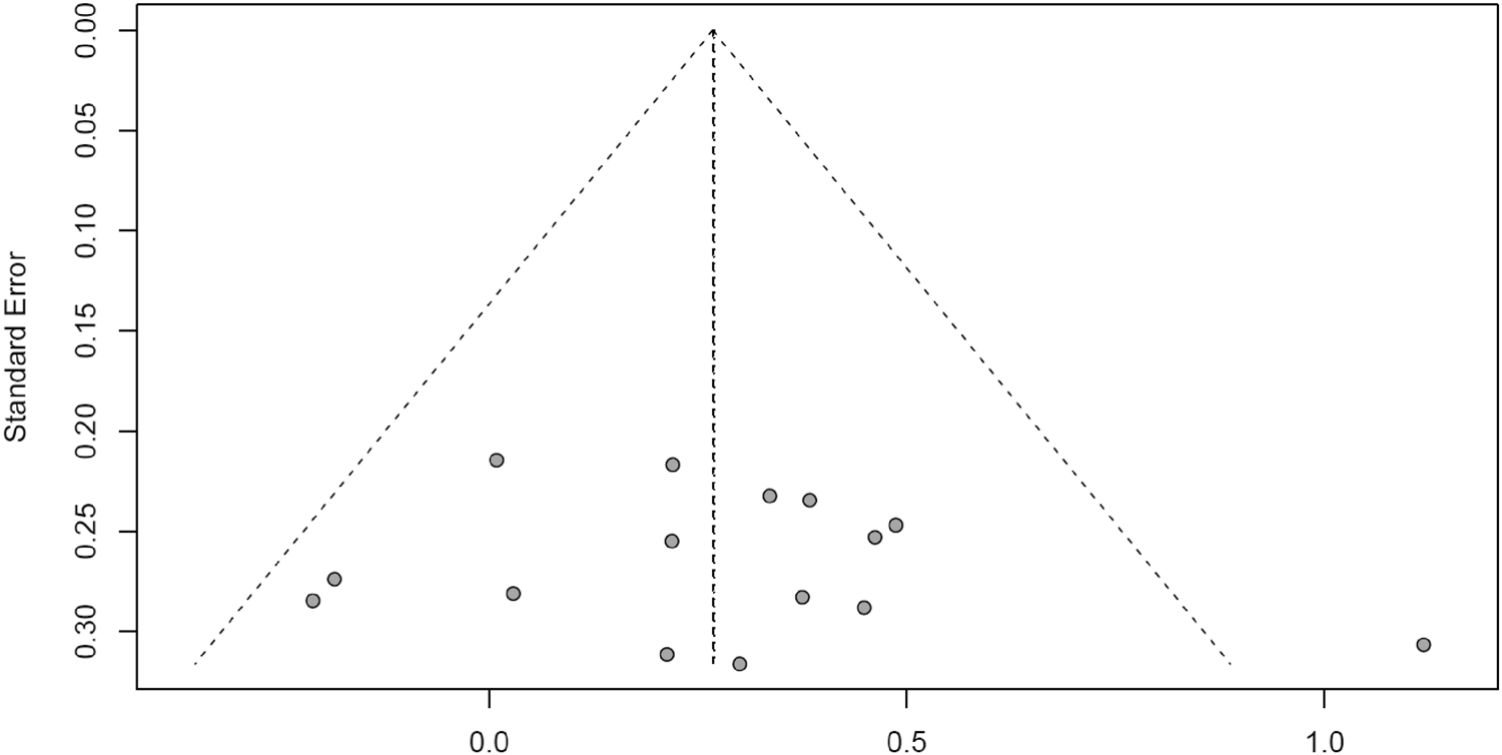
Funnel plot of the effects of unplanned athletic movement on ankle plantarflexion angle.

Although it is generally recommended to assess publication bias only when at least 10 studies are available (see chapter: study quality assessment), we chose to exploratively examine this risk for the effects of unplanned conditions on ankle plantarflexion (9 studies) by creating a funnel plot, including the outlier effect size that was excluded in the sensitivity analysis. Despite the fact that all included studies had small sample sizes, visual inspection of the funnel plot revealed no clear asymmetry, suggesting no strong evidence of publication bias (Fig. 9). For the remaining outcomes (including ankle dorsiflexion angles), an assessment of publication bias was not feasible due to the limited number of studies, and thus, the potential presence of bias cannot be ruled out.

#### Subgroup analyses

##### Ankle plantarflexion angles

Compared to jump landings, running tasks were significantly associated with greater ankle plantarflexion angles in the unplanned versus the pre-planned condition (Table 5). Additionally, plantarflexion angles at initial contact were significantly higher in unplanned conditions (Table 5). Although plantarflexion angles during the weight acceptance phase also tended to be higher under the unplanned condition, this difference did not reach statistical significance. Elite athletes demonstrated significantly greater plantarflexion angles in the unplanned compared to the pre-planned conditions, an effect not observed in non-elite athletes. Moreover, between-condition differences were significant when participants were required to respond to more than two visual stimuli, whereas no significant difference was found when the task involved a maximum of two stimuli. Sex had no significant effect on the between-condition differences in ankle plantarflexion angles (p > 0.05; Table 5). Additionally, the between-condition differences did not significantly differ between the corresponding moderator levels, including movement task (running vs. jump landing), standing/landing phase (initial contact vs. weight acceptance), expertise level (elite vs. non-elite athletes), and cognitive load (≤ 2 stimuli vs. > 2 stimuli) (all p > 0.05; Table 5). These results are based on all available effect sizes, including outliers, as there were insufficient data to conduct the analysis without them. The potential influence of available response time (436 ± 225 ms, range: 250–750 ms, 2/9 studies unclear) and running approach speed (4 ± 1.3 m/s, range: 2.6–5.5 m/s, 1/5 studies unclear) could not be examined due to the limited number of studies.

**Table 5:** Results of the subgroup analysis (effects of unplanned athletic movement on ankle plantarflexion angle within and between the moderator levels) including all effects sizes.

Moderator	No. of effect sizes/studies/ participants	Mean estimate (95% CI)	p-value	ω^2^/I^2^
**Sex**				0/30%
Females	9/6/103	0.34 (−0.004 to 0.69)	0.052	
Males	7/5/77	0.24 (−0.045 to 0.53)	0.085	
Females vs. males		−0.10 (−0.57 to 0.36)	0.607	
**Expertise Level**				0/38%
Elite	7/4/85	0.35 (0.02 to 0.69)	**0.042**	
Non-elite	8/5/95	0.18 (−0.12 to 0.48)	0.199	
Elite vs. Non-elite		−0.17 (−0.62 to 0.28)	0.394	
**Movement task**				0/69%
Running	7/5/91	0.36 (0.01 to 0.72)	**0.045**	
Landing	8/4/79	0.19 (−0.03 to 0.40)	0.086	
Running vs. Landing		−0.18 (−0.59 to 0.24)	0.340	
**Stance/landing phase**				0/68%
Initial contact	9/5/107	0.24 (0.06 to 0.43)	**0.017**	
Weight acceptance	5/4/79	0.40 (−0.03 to 0.82)	0.063	
Initial contact vs. weight acceptance		0.15 (−0.25 to 0.55)	0.394	
**Number of stimuli**				0/64%
≤ 2 stimuli		0.13 (−0.24 to 0.49)	0.430	
> 2 stimuli	8/5/105	0.36 (0.07 to 0.66)	**0.023**	
≤ 2 stimuli vs. > 2 stimuli	7/4/75	0.23 (−0.24 to 0.70)	0.280	

##### Ankle dorsiflexion angles

The subgroup analysis for the effects of unplanned movement on ankle dorsiflexion angles excluding the outlier SMD by Whyte (sensitivity analysis) was only possible for the moderator movement task (running vs. jump landing). The results indicated that neither running (SMD: 0.31, p = 0.14, 4 studies, 6 effect sizes) nor jump-landing (SMD: 0.19, p = 0.13, 4 studies, 7 effect sizes) had a significant effect on the between-condition effects. The between-condition differences in dorsiflexion angles did also not significantly differ between the two moderator levels (SMD: −0.12, p = 0.59, I^2^ = 65%). The potential influence of available response time (dorsiflexion: 321 ± 149 ms, range: 150–450 ms, 1/8 studies unclear) and running approach speed (dorsiflexion: 4.2 ± 1.2 m/s, range: 3.5–5.5 m/s, 1/4 studies unclear) could not be examined due to the limited number of studies. In most studies, peak dorsiflexion angles during unplanned conditions were observed during the stance phase (e.g., at midstance or between initial contact and peak knee or hip flexion), which prevented a moderator analysis across different phases.

### Qualitative synthesis

The qualitative synthesis did not suggest a clear significant effect of unplanned movement on ankle biomechanics. Regarding ankle kinematics, one from four studies^54^ found significantly higher external rotation angles for the unplanned conditions and neither of two studies^48,50^ found that unplanned movement resulted in higher internal rotation angles. For abduction angles (one study^51^), it was unclear whether the between-condition differences were statistically significant (due to poor reporting). For full details of the qualitative results, refer to Table 6.

**Table 6:** Results of the qualitative synthesis of the Effects of unplanned movement on ankle kinematics (joint angles).

	**UN < PP**	**UN > PP**	**UN = PP**	**Comment**
***Abduction angle***				
Stoffel^51^	X? run and sidestep condition	X? straight run condition	X?	Based on descriptive values of the full-text paper, but inference statistics were not reported for this outcome.
***Internal rotation angle***				
Meinerz^50^	X			Based on full-text paper (table of results with inference statistics)
Kim 2014^48^			X side-cutting condition	Based on full-text paper (table of results with inference statistics)
***External rotation angle***				
Kim 2014^48^			X cross-cutting condition	Based on full-text paper (table of results with inference statistics)
Kim 2016^47^			X	Based on full-text paper (table of results with inference statistics)
Whyte A^54^		X		Based on original data sent by authors (including inference statistics)
Zou^52^	X (at weight acceptance phase)		X (at initial contact)	Based on full-text paper (table of results with inference statistics)

UN < PP: unplanned condition resulted in significantly lower kinematic values compared to the pre-planned condition, UN > PP: unplanned condition resulted in significantly higher kinematic values compared to the pre-planned condition, UN = PP: no significant different kinematic values between the unplanned and pre-planned condition. X?: besides the descriptive mean values of the full-text papers, inference statistics were not reported/available. Thus, it was unclear whether the descriptive differences were significantly different.

Regarding ankle kinetics, one of two studies^48^ found higher external rotation moments for the unplanned versus the preplanned conditions. For plantarflexion (two studies^47,58^) and inversion/supination (three studies^48,53,56^) moments, no significantly higher values were verified for the unplanned conditions. For abduction and adduction moments (one study each^51^), it was unclear whether the higher mean values of the unplanned condition were significantly different (due to incomplete reporting). For full details of the qualitative results, refer to Table 7.

**Table 7:** Results of the qualitative synthesis of the effects of unplanned movement on ankle kinetics (joint moments).

	**UN < PP**	**UN > PP**	**UN = PP**	**Comment**
***Plantarflexion moments***				
Kim 2016^47^	X weight acceptance phase			Based on full-text paper (table of results with inference statistics)
Weinhandl^58^			X	
***Inversion/supination moments***				
Kim 2014^48^			X	Based on full-text paper (table of results with inference statistics)
Mache^56^			X	Based on full-text paper (table of results with inference statistics)
Richwalski^53^			X	Based on full-text paper (table of results with inference statistics)
***Abduction moments***				
Stoffel^51^		X?	X?	Based on descriptive values of the full-text paper, but inference statistics were not reported for this outcome.
***Adduction moments***				
Stoffel^51^	X? straight run condition	X? run and sidestep condition	X?	Based on descriptive values of the full-text paper, but inference statistics were not reported for this outcome.
***External rotation moments***				
Kim 2014^48^		X cross-cutting condition		Based on full-text paper (table of results with inference statistics)
Richwalski^53^			X	Based on full-text paper (table of results with inference statistics)

UN < PP: unplanned condition resulted in significantly lower kinetic values compared to the pre-planned condition, UN > PP: unplanned condition resulted in significantly higher kinetic values compared to the pre-planned condition, UN = PP: no significant different kinetic values between the unplanned and pre-planned condition. X?: besides the descriptive mean values of the full-text papers, inference statistics were not reported/available. Thus, it was unclear whether the descriptive differences were significantly different.

## Discussion

This systematic review and meta-analysis provides low to moderate certainty evidence that unplanned decision-making during athletic movement leads to slightly greater ankle plantarflexion angles compared to pre-planned conditions. Exploratory findings from our subgroup analysis suggested that the plantarflexion effects were more pronounced in running tasks and at initial contact. Effects were also significant in elite athletes and during tasks involving more complex decision-making (i.e., more than two stimuli), but not with respect to sex. However, the between-condition differences were not statistically significant between the corresponding moderator levels, indicating no interaction effects. Sensitivity analysis further indicated that unplanned movement tasks were associated with significantly greater ankle dorsiflexion angles, typically observed during later landing/stance phases than the plantarflexion effects, again with a small effect size. Also subtle biomechanical changes in the sagittal plane may have functional relevance in complex movements. These could represent potential injury risk factors, protective mechanisms, or performance-modifying adaptations, as discussed in the following sections. In contrast to the sagittal plane, we found no conclusive evidence pointing to differences between the pre-planned and unplanned conditions in the frontal and transverse planes.

  .

### Potential injury risk factors

Aberrant ankle biomechanics have been associated with increased injury risk. In inversion traumas, the anterior talofibular ligament (ATFL) and the calcaneofibular ligament (CFL) are most frequently affected. The injury mechanism of the ATFL typically involves a combination of plantarflexion and inversion or internal rotation, whereas tears of the CFL are primarily linked to dorsiflexion combined with excessive inversion or internal rotational loads^6,62^. While inversion and internal rotation (i.e., frontal and transverse plane motions) are considered the primary stressors of the lateral ligament complex, plantarflexion and dorsiflexion are generally regarded as secondary contributors to lateral ankle sprain^6,62^. Although our finding of increased isolated ankle plantarflexion may suggest a potential predisposition to malleolar fractures^63^ or a contributing factor to lateral ankle sprains, these interpretations remain speculative. The absence of concomitant primary stressors,such as elevated inversion angles or moments,limits the ability to draw firm conclusions about its role as an injury risk factor in the context of unplanned movements. A similar consideration applies to the observed increase in dorsiflexion, which may relate to CFL injury mechanisms. However, without concurrent inversion or internal rotation, its relevance to ankle injury risk remains unclear. Moreover, the low certainty of evidence, combined with substantial heterogeneity across participants (e.g., sex, expertise level) and task characteristics (e.g., movement task, landing/contact phase), may have limited the detection of additional biomechanical risk factors in the frontal and transverse planes and highlights the need for future research to confirm these hypotheses. Apart from this, the assessment of ankle biomechanics in the frontal and transverse planes is also inherently less accurate, particularly when using skin-mounted reflective markers, resulting in reduced measurement reliability^64,65^. In addition, several studies did not employ multi-segment foot models, which would have allowed for a more precise analysis of the complex mechanics of the foot and ankle^66^. This substantially limits the certainty of evidence regarding the effects of unplanned movement on frontal and transverse ankle biomechanics. Future research should aim to address these gaps to clarify the potential role of unplanned movement in ankle injury risk.

Moreover, the effects of single-leg jump landings on ankle biomechanics were rarely investigated by the included studies. Consequently, we were unable to quantify the specific influence of this movement pattern—particularly since all jump landing tasks were grouped together regardless of whether they involved single- or double-leg landings, due to an insufficient number of studies available to analyse single-leg landings separately—on multiplanar ankle biomechanics. While both run-and-cut movements and jump landings are considered high-risk manoeuvres for ankle sprains^3^, single-leg landings may pose an especially high risk due to the substantial vertical impact forces involved. In such cases, body weight is absorbed by only one leg, increasing the mechanical load on the ankle joint^7^. Additionally, the uneven weight distribution inherent to single-leg landings,compared to more symmetrical movements like running,may further elevate the risk of sprains. Supporting this, evidence indicates that over 45% of ankle injuries in basketball players occur following failed landings, with ankle inversion identified as the predominant biomechanical injury mechanism^2,9^. In fact, our results provide tentative evidence regarding the potential injury relevance of single-leg landings. For instance, in most of the included single-leg landing trials, higher ankle inversion angles were observed under unplanned conditions^49,61^ (see forest plot, Fig. 4), along with elevated eversion and internal rotation moments at the ankle^48,50,53–55^ (see forest plot, Fig. 6 and 8). Conversely, for the running tasks, the between-condition effects were generally smaller. Thus, to better understand the potential effects of unplanned movement on biomechanical risk factors for ankle injuries, more studies are needed that specifically investigate single-leg landings and biomechanics in the frontal and transverse planes.

### Potential injury protective factors

Given that the lower limb joints function as an integrated system^25^, our findings suggest that increased ankle plantarflexion may serve as a protective mechanism to compensate for excessive knee joint loading during unplanned movements^20–23^. Under time constraints, neuromuscular feedforward control may be limited^20^, potentially hindering the effective absorption of peak ground reaction forces and joint loads, such as knee valgus stress^57,67,68^. This protective mechanism theory is supported by initial evidence indicating that a moderate increase in ankle plantarflexion or the use of a forefoot strike pattern at initial contact may reduce peak knee valgus moments^69,70^ and combined peak knee valgus and internal rotation moments^71^. It has been demonstrated that plantarflexion in a forefoot strike pattern prior to initial contact enhances the activation of the hamstrings and gastrocnemius muscles, which may help prevent excessive anterior tibia translation and thereby improve knee joint stability^72–74^. Moreover, a recent study found that greater ankle plantarflexion during landings is associated with lower knee impact loading, potentially reducing the risk of severe knee injuries^75^. However, it is not yet clear whether these relationships apply to both sexes^69^ or which plantarflexion angles optimally contribute to impact absorption during landing^76^. It should be noted that increased plantarflexion was primarily observed during the early landing phases, such as initial contact and weight acceptance, whereas peak dorsiflexion was predominantly assessed in later phases, such as stance phase or the time between initial contact and maximum knee flexion. In these phases, ankle dorsiflexion may contribute to impact absorption and joint stabilization, potentially reducing mechanical load on both the ankle and knee^77,78^. Specifically, by promoting a more stable alignment of the talocrural joint in a close-packed position, dorsiflexion may help prevent excessive motion in other planes—particularly when compared to the more unstable plantarflexed position, which places the narrowest portion of the talus within the ankle mortise and reduces bony stability^77,79^, and may compromise ankle stability if pronounced excessively. While the link between reduced dorsiflexion range of motion (ROM) and increased injury risk is typically viewed as a compensatory mechanism in individuals with restricted ankle mobility^80^, the dorsiflexion increases observed in this review were small and of questionable clinical relevance. Future studies are needed to determine whether these changes reflect a beneficial functional adaptation, even in individuals with unrestricted ROM.

### Potential performance-modifying factors

Our finding of more ankle plantarflexion during the unplanned condition may also serve as a performance-modifying factor, but it is unclear if in a negative or positive way. Subgroup analysis showed that the increase in plantarflexion was particularly prominent for running with subsequent side cutting, but not for jump landing tasks. The largest effects were observed at initial contact, whereas no significant effect was found during the weight acceptance phase. This is not surprising, as higher movement speeds, such as in running or sprinting, may increase the tendency to land with greater ankle plantarflexion^81,82^, typically associated with a forefoot strike pattern^83^. Such contact may facilitate quicker push-off (e.g. shorter ground contact times^84^) and enhance efficiency and power during forward propulsion^27^. However, explosive performance in powerful movements requiring stretch–shortening cycles may benefit from an increase in dorsiflexion rather than plantarflexion. At dorsiflexion, the Achilles tendon is stiffened, which enhances energy storage and contributes to elastic recoil^28^. In this context, the more pronounced plantarflexion may hence also reduce the capacity to perform subsequent changes of direction due to less effective tendon loading. Conversely, the slightly increased dorsiflexion mostly observed during the stance phase (i.e., after initial contact and weight acceptance) may support stretch–shortening cycle performance and enhance explosive force production during push-off. As subgroup analysis was not feasible for dorsiflexion, the moderating influence of movement task (running or jump-landing) or athlete expertise remains unclear.

However, the slightly increased plantarflexion during unplanned conditions was statistically significant only among elite or semi-professional athletes, but not in amateur or recreational athletes. This suggests that elite athletes may be better able to adapt their movement strategies to specific contextual demands. In unplanned conditions, they may adopt biomechanically optimized patterns—such as increased plantarflexion at initial contact—to respond more quickly and efficiently, facilitating feedforward planning of landing and subsequent cutting despite heightened perceptual-cognitive demands. While greater plantarflexion may support faster reaction and shorter ground contact times, it could also limit dorsiflexion-dependent mechanisms, such as energy storage and elastic recoil, during the subsequent movement phase^28^. Elite athletes appear to exhibit superior perception–action coupling^85–87^, The ability to effectively integrate cognitive and motor processes during dynamic sports movements may make unplanned tasks less challenging for them, aligning with the findings from a similar review^22^. This reflects a higher degree of sensorimotor adaptability, potentially enabling them to differentiate between task demands and select the most effective movement strategy to maintain or even improve performance—for example, by prioritizing movement speed in run-and-cut tasks through increased plantarflexion at initial contact. In contrast, recreational athletes with lower levels of expertise may rely on more generalized or rigid patterns, regardless of the specific decision-making context.

Interestingly, the effect of unplanned movement on ankle plantarflexion was significant only in tasks that required a choice reaction to three stimuli (e.g., left, right, straight), whereas no significant differences were observed in tasks involving two or fewer stimuli. This finding suggests that more complex decision-making demands may trigger movement adaptations, like increased plantarflexion that could modify performance, for example by reducing ground contact times and enhancing forward propulsion, but potentially at the expense of effective stretch–shortening cycle performance. However, we were unable to determine whether this effect was more pronounced in elite athletes or specific movement tasks, as these interactions were not specifically analysed. These findings should therefore be regarded as exploratory.

Although female athletes tend to sustain ankle injuries at higher rates and with greater severity compared to males^42^, the subgroup analysis did not show that the effect of unplanned movement on ankle plantarflexion was slightly more pronounced in females. This lack of sex-specific difference further supports the idea that the observed increase in isolated plantarflexion may more likely reflect a strategy to modify or optimize performance rather than an injury risk factor. However, since no statistically significant interactions were observed between movement task (running vs. jump landing), contact phase (initial contact vs. weight acceptance), expertise level (elite vs. non-elite), task complexity (≤ 2 vs. > 2 stimuli), and the pre- versus unplanned movement conditions, these subgroup-specific findings should be interpreted with caution and regarded as hypothesis-generating rather than conclusive evidence of moderator-specific effects. .

### Practical implications

Our results suggest potential small effects of unplanned movements on ankle biomechanics, but there is insufficient evidence to draw specific conclusions or practical recommendations. Nonetheless, coaches and therapists should be aware that unplanned movement tasks affect not only knee biomechanics but also ankle joint kinematics. The ankle should therefore be considered during high-risk, unplanned movements, even though it is currently uncertain whether observed increases in ankle plantarflexion and dorsiflexion during unplanned versus pre-planned tasks represent injury risk factors or performance-modifying strategies. In line with previous recommendations^88–90^ we encourage practitioners working with team sport athletes to incorporate neurocognitive challenges—such as time-constrained decision-making or distractions—into motor exercises and injury risk screenings (e.g., balance, jump landing, change-of-direction tasks) to improve ecological validity. Examples of such training or assessment tasks could include agility drills requiring reactive change of direction, unplanned landing and cutting maneuvers that demand time-constrained motor responses to visual cues (e.g., validated stationary^91,92^ or mobile setups, such as sensor-based systems^93,94^). Another task type may involve dual-task scenarios, such as jumping or cutting combined with simultaneous cognitive challenges such as counting backward or performing a Stroop interference test, all of which can influence lower limb biomechanics^24^. Incorporating these perceptual-cognitive demands into motor training and assessments may enhance ecological validity and better facilitate transfer to on-field performance^95^. Task complexity can be increased by adding more possible movement decisions (e.g., more visual stimuli) or by combining decision-making with additional cognitive tasks or distractions (e.g., catching a ball during single-leg landing exercises). Higher-performing athletes may require more complex tasks to sufficiently challenge their perception–action coupling, while recreational or amateur athletes may benefit from starting with lower motor-cognitive demands. Importantly, movement quality (e.g., safe landing technique) should be prioritized before increasing task complexity.

### Limitations and future research

A major strength of our review is that the evidence provided is based on studies with relatively high homogeneity of the investigated participants (e.g., age, BMI, sports, injury history) and the use of three-dimensional measurements by all included trials, which represents the gold standard in biomechanical analyses^96^. The methodological quality of the included studies was fair to high.

However, limitations include using an adapted Downs and Black checklist for quality assessment, as no suitable checklist exists for non-randomized cross-sectional studies. We included only studies published in English or German, and no polyglot search was conducted. This may have resulted in the exclusion of relevant studies published in other languages and introduced publication bias. Furthermore, the limited certainty of the evidence—particularly regarding ankle biomechanics in the frontal and transverse planes—hinders the ability to draw definitive conclusions about injury risk (e.g., ankle sprain). Ankle kinetics in the sagittal plane were infrequently assessed (plantarflexion) or showed considerable heterogeneity (dorsiflexion, see forest plot, Fig. 3), limiting our ability to draw firm conclusions.

Due to an insufficient number of studies and poor reporting, the potential impact of other important moderators of sagittal plane kinematics—such as running approach speed or available response time—could not be quantified through subgroup analysis, which may have affected the robustness of the findings. Unlike jump landings, where the time available to react to an unplanned stimulus is primarily constrained by flight time, run-and-cut tasks allow participants to actively reduce their approach speed, thereby gaining additional time for motor adjustments^22^. This strategy was evident in one of the included running trials^52^, whereas the remaining studies^47,48,51,58^ did not report any between-condition differences in running approach speed. Existing evidence suggests that running velocity influences lower limb joint mechanics during unplanned cutting manoeuvres^21,97,98^. During jump landings, variations in available response time (e.g. jump height) may also influence ankle biomechanics. For example, shorter response windows could result in prolonged ground contact times before initiating subsequent movements (e.g., cutting), possibly as a strategy to maximize decision-making time^22^.

To draw more definitive conclusions and recommendations, further studies with high methodological rigor are needed to compare the effects of unplanned versus pre-planned athletic movements on multiplanar ankle biomechanics. While changes in the sagittal plane have been observed, it remains unclear whether increased ankle plantarflexion and dorsiflexion during unplanned movements represent a risk factor or a performance-modifying or -enhancing adaptation. As such, it is not yet possible to recommend whether these movement patterns should be encouraged or mitigated. Beyond sagittal plane mechanics, high-quality studies should investigate the effects of unplanned decision-making during sport-specific tasks on frontal and transverse ankle biomechanics. This is particularly important for understanding the potential injury risk associated with cognitively demanding movement tasks. To achieve this, studies using advanced methodological approaches capable of detecting subtle changes across all planes of motion are required. Given that most existing studies in this area are cross-sectional, prospective trials are crucial to determine whether altered multiplanar ankle biomechanics under unplanned conditions can predict injury risk. Understanding the relationships between these biomechanical changes and injuries—such as ankle sprains or knee injuries—in highly dynamic and variable sports contexts will be essential for advancing injury prevention strategies. Finally, our subgroup analysis identified several promising participants- and task-related moderators that may influence ankle plantarflexion angles during unplanned movement conditions. Future research should prioritize investigating important task-related moderators—such as running speed or available response time, movement task (e.g., run-and-cut vs. jump landing) and stance phase (e.g., initial contact vs. weight acceptance)—before focusing on participant-related moderators like sex or expertise level. This stepwise approach may help to better understand the effects of unplanned conditions on ankle biomechanics first, and then determine which participant characteristics may moderate these effects. In turn, this could support more individualized training and assessment approaches—tailored, for example, to an athlete’s expertise level.

## Conclusion

Low to moderate certainty evidence suggests that unplanned movements evokes increased ankle plantarflexion (run-and-cut tasks, initial ground contact, and elite athletes) and dorsiflexion (mainly observed during the stance phase) angles compared to pre-planned conditions, with small effect sizes. Given the limited number of studies, predominantly low certainty of evidence, small sagittal plane effects, and limited findings in the frontal and transverse planes, these results should be interpreted with caution. It is not yet possible to recommend whether increased ankle plantarflexion and dorsiflexion during unplanned movements should be encouraged for potential performance benefits or mitigated due to possible injury risk, as no prospective studies have linked these biomechanical patterns to injury rates or performance outcomes. This remains an important direction for future research. High-quality studies are especially needed to assess unplanned decision-making during sport-specific tasks, not only in the sagittal, but also in the frontal and transverse planes, and in relation to task-related moderators, in order to draw more definitive conclusions and inform evidence-based recommendations for injury prevention and performance enhancement in team and interceptive sports.

## Supplementary Information

Supplementary Information 1.

Supplementary Information 2.

Supplementary Information 3.

Supplementary Information 4.

Supplementary Information 5.

Supplementary Information 6.

## Data Availability

All the information supporting the findings of this study can be found within the paper and its supplementary materials. The datasets used and/or analysed during the current study are available from the corresponding author on reasonable request.

## Abbreviations

SD: Standard deviations
SMD: Standardized mean differences
SE: Standard errors
CI: Confidence intervals
RE: Random effects,
I^2^: Between-study heterogeneity.
ATFL: Anterior talofibular ligament
CFL: Calcaneofibular ligament
