# Supplementary material for: Effects of sport specific unplanned movements on ankle kinetics and kinematics in healthy athletes from systematic review with meta-analysis
Source: Sci Rep. 2025 Sep 12;15:32476. doi: 10.1038/s41598-025-18746-9 (PMC12432200; doi:10.1038/s41598-025-18746-9)
Supplement: Supplementary file 3 — Supplementary Information 3. [file 41598_2025_18746_MOESM3_ESM.pdf]

Search terms

PubMed/MEDLINE: Ankle AND (anticipation OR "decision-making" OR "preparation time" OR unplanned OR unanticipated OR anticipated OR anticipatory) AND (biomechanics OR kinematics OR kinetics OR posture OR loading OR "ground reaction")

Science direct search (sorted based on date, research article): ankle AND (unplanned OR unanticipated) AND (biomechanics OR kinetics OR kinematics)

Cochrane library search (sorted based on relevance): Ankle AND (anticipation OR "decision-making" OR "preparation time" OR unplanned OR unanticipated OR anticipated OR anticipatory) AND (biomechanics OR kinematics OR kinetics OR posture OR loading OR "ground reaction")

Google Scholar search (based on relevance): Ankle AND (anticipation OR "decision-making" OR "preparation time" OR unplanned OR unanticipated OR anticipated OR anticipatory) AND (biomechanics OR kinematics OR kinetics OR posture OR loading OR "ground reaction")

Exclusion criteria

- Duplicates = 2
- Wrong Topic (e.g. no unanticipated/unplanned, non-decision-making/non-choice-reaction conditions during athletic movements) = 3
- No assessment of ankle kinematic/kinetics (e.g. only EMG, intersegmental coordination, coupling angles, joint work/power, centre of pressure, time to stabilisation, foot placement, foot progression angle) =4
- No preplanned/anticipated control condition = 5
- Not healthy participants = 6
- Study Design or no peer-review publication (e.g. systematic review, not a trial, not in vivo, animals, retrospective, case-study, trial registration, preprint, thesis, conference abstract) = 7
- Others (performing movement tasks with additional weights, walking on uneven terrain, gait initiation/termination, landing unexpectedly on inclinated/inverted platforms, unexpected perturbations, movement tasks not comparable between conditions) = 8
- Relevant data for inclusion into quantitative/qualitative synthesis not reported/provided by the authors after requesting it = 9
