# Supplementary material for: Effects of sport specific unplanned movements on ankle kinetics and kinematics in healthy athletes from systematic review with meta-analysis
Source: Sci Rep. 2025 Sep 12;15:32476. doi: 10.1038/s41598-025-18746-9 (PMC12432200; doi:10.1038/s41598-025-18746-9)
Supplement: Supplementary file 4 — Supplementary Information 4. [file 41598_2025_18746_MOESM4_ESM.pdf]

Database	Title	Authors	Journals	Exclusion criteria*
MEDLINE/PUBMED	The effect of postural changes upon the ankle arterial perfusion pressure	Pollak EW, Chavis P, Wolfman EF.	Vasc Surg. 1976 Sep-Oct	3
	Surgical implications of biomechanics of the foot and ankle	Mann RA.	Clin Orthop Relat Res. 1980 Jan-Feb	3
	Current concepts of surgical management of deformities of the lower extremities in cerebral palsy	Samilson RL.	Clin Orthop Relat Res. 1981 Jul-Aug	3
	[Postural adjustments associated with voluntary mobilization of the arm in microgravity conditions]	Clément G, Gurfinkel VS, Lestienne F, Lipchits MI, Popov KE.	C R Seances Acad Sci III. 1983	3
	Posture and the arterial pressure in the ischaemic foot	Coni NK.	Age Ageing. 1983 May	3
	Examination of the cerebral palsy patient with foot and ankle problems	Kasser JR, MacEwen GD.	Foot Ankle. 1983 Nov-Dec	3
	Adaptation of postural control to weightlessness	Clément G, Gurfinkel VS, Lestienne F, Lipshits MI, Popov KE.	Exp Brain Res. 1984	3
	Preparatory process for anticipatory postural adjustments: modulation of leg muscles reflex pathways during preparation for arm movements in standing man	Woollacott MH, Bonnet M, Yabe K.	Exp Brain Res. 1984	3
	Changes of posture during transient perturbations in microgravity	Clément G, Gurfinkel VS, Lestienne F, Lipshits MI, Popov KE.	Aviat Space Environ Med. 1985 Jul	3
	The biomechanics of lower extremity action in distance running	Cavanagh PR.	Foot Ankle. 1987 Feb	3
	Response synergies over a single leg when it is perturbed during the complex rhythmic movement of pedalling	McIlroy WE, Brooke JD.	Brain Res. 1987 Mar 31	3
	A computer-controlled system to perturb the ankle joint of freely standing cats trained to maintain a given force	Sinkjaer T, Hoffer JA.	J Neurosci Methods. 1987 Oct	3
	Postural synergies in axial movements: short and long-term adaptation	Pedotti A, Crenna P, Deat A, Frigo C, Massion J.	Exp Brain Res. 1989	3
	Influence of central set on human postural responses	Horak FB, Diener HC, Nashner LM.	J Neurophysiol. 1989 Oct	3
	Postural maintenance during movement: simulations of a two joint model	Ramos CF, Stark LW.	Biol Cybern. 1990	3
	Three-dimensional lower extremity joint kinetics in normal pediatric gait	Ounpuu S, Gage JR, Davis RB.	J Pediatr Orthop. 1991 May-Jun	3
	Phase-dependent modulations of anticipatory postural activity during human locomotion	Hirschfeld H, Forssberg H.	J Neurophysiol. 1991 Jul	3
	Long latency postural responses are functionally modified by cognitive set	Beckley DJ, Bloem BR, Remler MP, Roos RA, Van Dijk JG.	Electroencephalogr Clin Neurophysiol. 1991 Oct	3
	Do fast voluntary movements necessitate anticipatory postural adjustments even if equilibrium is unstable?	Nouillot P, Bouisset S, Do MC.	Neurosci Lett. 1992 Nov 23	3
	Foot and ankle manifestations of Charcot-Marie-Tooth disease	Holmes JR, Hansen ST Jr.	Foot Ankle. 1993 Oct	3
	Radiographic alterations in the foot following toe-to-thumb free transfer	Tiamfook T, Sartoris DJ, Goldberg J.	J Foot Ankle Surg. 1994 May-Jun	3
	Cerebellar control of postural scaling and central set in stance	Horak FB, Diener HC.	J Neurophysiol. 1994 Aug	3
	Patterns of knee arthrosis and patellar subluxation	Harrison MM, Cooke TD, Fisher SB, Griffin MP.	Clin Orthop Relat Res. 1994 Dec	3
	Directional specificity of postural muscles in feed-forward postural reactions during fast voluntary arm movements	Aruin AS, Latash ML.	Exp Brain Res. 1995	3
	The role of anticipatory postural adjustments and gravity in gait initiation	Lepers R, Brenière Y.	Exp Brain Res. 1995	3
	Kinematic determinants of human locomotion	Borghese NA, Bianchi L, Lacquaniti F.	J Physiol. 1996 Aug 1	3
	Measurement of plantar pressure distribution during gait for diagnosis of functional lateral ankle instability	Becker H, Rosenbaum D, Claes L, Gerngro H.	Clin Biomech (Bristol, Avon). 1997 Apr	3
	Changes in the H-reflexes of ankle extensor and flexor muscles at the initiation of a stepping movement in humans	Komiyama T, Kasai T.	Brain Res. 1997 Aug 22	3
	Gait initiation in Parkinson's disease	Rosin R, Topka H, Dichgans J.	Mov Disord. 1997 Sep	3
	The influence of articular surface incongruity on lubrication and contact pressure distribution of loaded synovial joints	Hlavácek M, Vokoun D.	Proc Inst Mech Eng H. 1998	3
	Stiffness control of balance in quiet standing	Winter DA, Patla AE, Prince F, Ishac M, Gielo-Perczak K.	J Neurophysiol. 1998 Sep	3
	Changes to the gait initiation programme following a running exercise in human subjects	Lepers R, Brenière Y, Maton B.	Neurosci Lett. 1999 Jan 22	3
	Age-related kinetic changes in normal pediatrics	Cupp T, Oeffinger D, Tylkowski C, Augsburger S.	J Pediatr Orthop. 1999 Jul-Aug	3
	Adaptive motor strategy for squatting in spastic diplegia	Dan B, Bouillot E, Bengoetxea A, Noël P, Kahn A, Cheron G.	Eur J Paediatr Neurol. 1999	3
	Flat foot functional evaluation using pattern recognition of ground reaction data	Bertani A, Cappello A, Benedetti MG, Simoncini L, Catani F.	Clin Biomech (Bristol, Avon). 1999 Aug	3
	NACOB presentation CSB New Investigator Award. Balance recovery from medio-lateral perturbations of the upper body during standing. North American Congress on Biomechanics	Rietdyk S, Patla AE, Winter DA, Ishac MG, Little CE.	J Biomech. 1999 Nov	3
	The roles of proximal and distal muscles in anticipatory postural adjustments under asymmetrical perturbations and during standing on rollerskates	Shiratori T, Latash M.	Clin Neurophysiol. 2000 Apr	3
	Intrathecal baclofen normalizes motor strategy for squatting in familial spastic paraplegia: a case study	Dan B, Cheron G.	Neurophysiol Clin. 2000 Feb	3
	Anticipatory postural adjustments depend on final equilibrium and task complexity in vertical high jump movements	Le Pellec A, Maton B.	J Electromyogr Kinesiol. 2000 Jun	3
	Development of postural adjustment during gait initiation: kinematic and EMG analysis	Assaiante C, Woollacott M, Amblard B.	J Mot Behav. 2000 Sep	3
	The variability of goniometric measurements in ambulatory children with spastic cerebral palsy	McDowell BC, Hewitt V, Nurse A, Weston T, Baker R.	Gait Posture. 2000 Oct	3
	Anticipatory locomotor control for obstacle avoidance in mid-childhood aged children	McFadyen BJ, Malouin F, Dumas F.	Gait Posture. 2001 Feb	3
	Anticipatory postural adjustments associated with lateral and rotational perturbations during standing	Aruin AS, Ota T, Latash ML.	J Electromyogr Kinesiol. 2001 Feb	3
	Dynamic biomechanical model of the hand and arm in pistol grip power handtool usage	Lin JH, Radwin RG, Richard TG.	Ergonomics. 2001 Feb 20	3
	Kinematic approach to gait analysis in patients with rheumatoid arthritis involving the knee joint	Sakauchi M, Narushima K, Sone H, Kamimaki Y, Yamazaki Y,	Arthritis Rheum. 2001 Feb	3
	Biomechanical analysis of movement strategies in human forward trunk bending. II. Experimental study	Alexandrov AV, Frolov AA, Massion J.	Biol Cybern. 2001 Jun	3
	Normative data for passive ankle plantarflexion--dorsiflexion flexibility	Moseley AM, Crosbie J, Adams R.	Clin Biomech (Bristol, Avon). 2001 Jul	3
	Simulating mechanical consequences of voluntary movement upon whole-body equilibrium: the arm-raising paradigm revisited	Pozzo T, Ouamer M, Gentil C.	Biol Cybern. 2001 Jul	3
	Distinct multi-joint control strategies in spastic diplegia associated with prematurity or Angelman syndrome	Dan B, Bouillot E, Bengoetxea A, Boyd SG, Cheron G.	Clin Neurophysiol. 2001 Sep	3
	Effects of lower limb torsion on ankle kinematic data during gait analysis	Song KM, Concha MC, Haideri NF.	J Pediatr Orthop. 2001 Nov-Dec	3
	Effect of vibration-induced postural illusion on anticipatory postural adjustment of voluntary arm movement in standing humans	Kasai T, Yahagi S, Shimura K.	Gait Posture. 2002 Feb	3
	Anticipatory control of center of mass and joint stability during voluntary arm movement from a standing posture: interplay between active and passive control	Patla AE, Ishac MG, Winter DA.	Exp Brain Res. 2002 Apr	3
	Human balancing of an inverted pendulum: position control by small, ballistic-like, throw and catch movements	Loram ID, Lakie M.	J Physiol. 2002 May 1	3
	Contrast material travel times in patients undergoing peripheral MR angiography	Prince MR, Chabra SG, Watts R, Chen CZ, Winchester PA, K	Radiology. 2002 Jul	3
	Computer assisted surgery for total knee arthroplasty	Nizard R.	Acta Orthop Belg. 2002 Jun	3
	The impact of instrumented gait analysis on surgical planning: treatment of spastic equinovarus deformity of the foot and ankle	Fuller DA, Keenan MA, Esquenazi A, Whyte J, Mayer NH, Fid	Foot Ankle Int. 2002 Aug	3
	Ankle muscle stiffness alone cannot stabilize balance during quiet standing	Morasso PG, Sanguineti V.	J Neurophysiol. 2002 Oct	3
	Voluntary toe-walking gait initiation: electromyographical and biomechanical aspects	Couillandre A, Maton B, Brenière Y.	Exp Brain Res. 2002 Dec	3
	Kinematic behavior of the ankle following malleolar fracture repair in a high-fidelity cadaver model	Michelson JD, Hamel AJ, Buczek FL, Sharkey NA.	J Bone Joint Surg Am. 2002 Nov	3
	The study of muscle action during single support and swing phase of gait: clinical relevance of forward simulation techniques	Jonkers I, Stewart C, Spaepen A.	Gait Posture. 2003 Apr	3
	Kinematic adaptations of spinal cord-injured subjects during obstructed walking	Ladouceur M, Barbeau H, McFadyen BJ.	Neurorehabil Neural Repair. 2003 Mar	3
	Foot kinematics and kinetics during adolescent gait	MacWilliams BA, Cowley M, Nicholson DE.	Gait Posture. 2003 Jun	3
	Human balancing of an inverted pendulum with a compliant linkage: neural control by anticipatory intermittent bias	Lakie M, Caplan N, Loram ID.	J Physiol. 2003 Aug 15	3
	Case for gait analysis as part of the management of incomplete spinal cord injury	Patrick JH.	Spinal Cord. 2003 Sep	3
	Importance of body sway velocity information in controlling ankle extensor activities during quiet stance	Masani K, Popovic MR, Nakazawa K, Kouzaki M, Nozaki D.	J Neurophysiol. 2003 Dec	3
	Anticipatory control related to the upward propulsive force during the rising on tiptoe from an upright standing position	Ito T, Azuma T, Yamashita N.	Eur J Appl Physiol. 2004 Jun	3
	Paradoxical muscle movement in human standing	Loram ID, Maganaris CN, Lakie M.	J Physiol. 2004 May 1	3
	Does postural chain mobility influence muscular control in sitting ramp pushes?	Le Bozec S, Bouisset S.	Exp Brain Res. 2004 Oct	3
	The sway-density curve and the underlying postural stabilization process	Jacono M, Casadio M, Morasso PG, Sanguineti V.	Motor Control. 2004 Jul	3
	Classification of equinus in ambulatory children with cerebral palsy-discrimination between dynamic tightness and fixed contracture	Zwick EB, Leistritz L, Milleit B, Saraph V, Zwick G, Galicki M,	Gait Posture. 2004 Dec	3
	Gender differences in the kinematics of unanticipated cutting in young athletes	Ford KR, Myer GD, Toms HE, Hewett TE.	Med Sci Sports Exerc. 2005 Jan	5
	Active, non-spring-like muscle movements in human postural sway: how might paradoxical changes in muscle length be produced?	Loram ID, Maganaris CN, Lakie M.	J Physiol. 2005 Apr 1	3
	Physically active older adults display alterations in gait initiation	Henriksson M, Hirschfeld H.	Gait Posture. 2005 Apr	3
	The heel-contact gait pattern of habitual toe walkers	Crenna P, Fedrizzi E, Andreucci E, Frigo C, Bono R.	Gait Posture. 2005 Apr	3
	Anticipatory locomotor adjustments of the trail limb during surface accommodation	Rietdyk S.	Gait Posture. 2006 Apr	3
	Spatio-temporal separation of roll and pitch balance-correcting commands in humans	Grüneberg C, Duysens J, Honegger F, Allum JH.	J Neurophysiol. 2005 Nov	3
	Deceleration affects anticipatory and reactive components of triggered postural responses	Carpenter MG, Thorstensson A, Cresswell AG.	Exp Brain Res. 2005 Dec	3
	Effect of age on anticipatory postural adjustments in unilateral arm movement	Bleuse S, Cassim F, Blatt JL, Labyt E, Derambure P, Guieu JI	Gait Posture. 2006 Oct	3
	The mechanical consequences of dynamic frontal plane limb alignment for non-contact ACL injury	Chaudhari AM, Andriacchi TP.	J Biomech. 2006	3
	Dynamic modeling and torque estimation of FES-assisted arm-free standing for paraplegics	Kim JY, Popovic MR, Mills JK.	IEEE Trans Neural Syst Rehabil Eng. 2006 Mar	3
	The effect of trial number on the emergence of the 'broken escalator' locomotor aftereffect	Bunday KL, Reynolds RF, Kaski D, Rao M, Salman S, Bronste	Exp Brain Res. 2006 Sep	3
	Slip-related muscle activation patterns in the stance leg during walking	Chambers AJ, Cham R.	Gait Posture. 2007 Apr	3
	Changes in lower limb kinematics, kinetics, and muscle activity in subjects with functional instability of the ankle joint during a single leg drop jump	Delahunt E, Monaghan K, Caulfield B.	J Orthop Res. 2006 Oct	3
	Biomechanics of lower limb raising from the supine position	Gatti R, Corti M, Cervi P, Pulici L, Boccardi S.	Eura Medicophys. 2006 Sep	3
	Alterations in plantar pressure with different walking boot designs	DiLiberto FE, Baumhauer JF, Wilding GE, Nawoczenski DA.	Foot Ankle Int. 2007 Jan	3
	Indications for orthoses to improve gait in children with cerebral palsy	Davids JR, Rowan F, Davis RB.	J Am Acad Orthop Surg. 2007 Mar	3
	Evidence of proactive forefoot control during landings on inclined surfaces	Morey-Klapsing G, Arampatzis A, Brüggemann GP.	J Mot Behav. 2007 Mar	3
	Coordination of rapid stepping with arm pointing: anticipatory changes and step adaptation	Yiou E, Schneider C, Roussel D.	Hum Mov Sci. 2007 Jun	3
	Neuromuscular and lower limb biomechanical differences exist between male and female elite adolescent soccer players during an unanticipated run and crosscut maneuver	Landry SC, McKean KA, Hubley-Kozey CL, Stanish WD, Delu	Am J Sports Med. 2007 Nov	5
	Classification of spastic hemiplegic cerebral palsy in children	Riad J, Haglund-Akerlind Y, Miller F.	J Pediatr Orthop. 2007 Oct-Nov	3
				1
	Why anticipatory postural adjustments in gait initiation need to be modified when stepping up onto a new level?	Gélat T, Le Pellec A.	Neurosci Lett. 2007 Dec 11	3
	Human stance control beyond steady state response and inverted pendulum simplification	Schweigart G, Mergner T.	Exp Brain Res. 2008 Mar	3
	Controlling propulsive forces in gait initiation in transfemoral amputees	van Keeken HG, Vrieling AH, Hof AL, Halbertsma JP, Schopp	J Biomech Eng. 2008 Feb	3
	Ankle bracing, plantar-flexion angle, and ankle muscle latencies during inversion stress in healthy participants	Kernozek T, Durall CJ, Friske A, Mussallem M.	J Athl Train. 2008 Jan-Mar	3
	Age differences between children and young adults in the dynamics of dual-task prioritization: body (balance) versus mind (memory)	Schaefer S, Krampe RT, Lindenberger U, Baltes PB.	Dev Psychol. 2008 May	3
	Altered postural control in anticipation of postural instability in persons with recurrent low back pain	Brumagne S, Janssens L, Janssens E, Goddyn L.	Gait Posture. 2008 Nov	3
	Mid-diaphyseal fibular fractures with syndesmotic disruption: should we plate the fibula?	Ho JY, Ren Y, Kelikian A, Aminian A, Charnley I, Zhang LQ.	Foot Ankle Int. 2008 Jun	3
	The influence of ankle support on postural control	Broglio SP, Monk A, Sopiarz K, Cooper ER.	J Sci Med Sport. 2009 May	3
	Results of treatment when orthopaedic surgeons follow gait-analysis recommendations in children with CP	Lofterød B, Terjesen T.	Dev Med Child Neurol. 2008 Jul	3

The effect of short-term changes in body mass distribution on feed-forward postural control	Li X, Aruin AS.	J Electromyogr Kinesiol. 2009 Oct	3
Can prepared anticipatory postural adjustments be updated by proprioception?	Ruget H, Blouin J, Teasdale N, Mouchnino L.	Neuroscience. 2008 Aug 26	3
Measuring venous insufficiency objectively in the clinical setting	Kelechi TJ, Bonham PA.	J Vasc Nurs. 2008 Sep	3
Design features of current total ankle replacements: implants and instrumentation	Cracchiolo A 3rd, Deorio JK.	J Am Acad Orthop Surg. 2008 Sep	3
Normative values for the Foot Posture Index	Redmond AC, Crane YZ, Menz HB.	J Foot Ankle Res. 2008 Jul 31	3
The influence of seat height on the mechanical function of the triceps surae muscles during steady-rate cycling	Sanderson DJ, Amoroso AT.	J Electromyogr Kinesiol. 2009 Dec	3
The recurrent clubfoot: can gait analysis help us make better preoperative decisions?	Sankar WN, Rethlefsen SA, Weiss J, Kay RM.	Clin Orthop Relat Res. 2009 May	3
The development of an intraoperative plantar pressure assessment device	Ellis SJ, Hillstrom H, Cheng R, Lipman J, Garrison G, Deland	Foot Ankle Int. 2009 Apr	3
Menstrual cycle variations in oestradiol and progesterone have no impact on in vivo medial gastrocnemius tendon mechanical properties	Burgess KE, Pearson SJ, Onambélé GL.	Clin Biomech (Bristol, Avon). 2009 Jul	3
Lower limb muscle activity and kinematics of an unanticipated cutting manoeuvre: a gender comparison	Beaulieu ML, Lamontagne M, Xu L.	Knee Surg Sports Traumatol Arthrosc. 2009 Aug	5
Factors associated with pelvic asymmetry in transverse plane during gait in patients with cerebral palsy	de Morais Filho MC, Kawamura CM, Andrade PH, Dos Santos	J Pediatr Orthop B. 2009 Nov	3
Neuromuscular control and ankle instability	Gutierrez GM, Kaminski TW, Douex AT.	PM R. 2009 Apr	3
The effect of heel height on gait and posture: a review of the literature	Cowley EE, Chevalier TL, Chockalingam N.	J Am Podiatr Med Assoc. 2009 Nov-Dec	3
Gait termination control strategies are altered in chronic ankle instability subjects	Wikstrom EA, Bishop MD, Inamdar AD, Hass CJ.	Med Sci Sports Exerc. 2010 Jan	3
Nonmedicinal therapy in the management of ankle arthritis	Rao S, Ellis SJ, Deland JT, Hillstrom H.	Curr Opin Rheumatol. 2010 Mar	3
Similarity of joint kinematics and muscle demands between elliptical training and walking: implications for practice	Burnfield JM, Shu Y, Buster T, Taylor A.	Phys Ther. 2010 Feb	3
Gait Analysis before and after Gastrocnemius Fascia Lengthening for Spastic Equinus Foot Deformity in a 10-Year-Old Diplegic Child	Galli M, Cimolin V, Santambrogio GC, Crivellini M, Albertini G.	Case Rep Med. 2010	3
			1
Stepping with an ankle foot orthosis re-examined: a mechanical perspective for clinical decision making	Nair PM, Rooney KL, Kautz SA, Behrman AL.	Clin Biomech (Bristol, Avon). 2010 Jul	3
On the role of knee joint in balance control and postural strategies: effects of total knee replacement in elderly subjects with knee osteoarthritis	Gauchard GC, Vançon G, Meyer P, Mainard D, Perrin PP.	Gait Posture. 2010 Jun	3
The use of gait analysis in the treatment of pediatric foot and ankle disorders	Theologis T, Stebbins J.	Foot Ankle Clin. 2010 Jun	3
Decoupling of stretch reflex and background muscle activity during anticipatory postural adjustments in humans	Vedula S, Kearney RE, Wagner R, Stapley PJ.	Exp Brain Res. 2010 Aug	3
Gender-specific neuromuscular activity of the M. peroneus longus in healthy runners - A descriptive laboratory study	Baur H, Hirschmüller A, Cassel M, Müller S, Mayer F.	Clin Biomech (Bristol, Avon). 2010 Nov	3
The foot and ankle in cerebral palsy	Davids JR.	Orthop Clin North Am. 2010 Oct	3
Dynamic versus fixed equinus deformity in children with cerebral palsy: how does the triceps surae muscle work?	Svehlík M, Zwick EB, Steinwender G, Kraus T, Linhart WE.	Arch Phys Med Rehabil. 2010 Dec	3
What variables influence the ability of an AFO to improve function and when are they indicated?	Malas BS.	Clin Orthop Relat Res. 2011 May	3
Arthrometric measurement of ankle-complex motion: normative values	Schwarz NA, Kovaleski JE, Heitman RJ, Gurchiek LR, Gubler-	J Athl Train. 2011 Mar-Apr	3
[Tendon transfers for peroneal palsy - functional outcome]	Kremer T, Riedel K, Germann G, Heitmann C, Sauerbier M.	Handchir Mikrochir Plast Chir. 2011 Apr	3
Biomechanical reorganisation of stepping initiation during acute dorsiflexor fatigue	Yiou E, Ditcharles S, Le Bozec S.	J Electromyogr Kinesiol. 2011 Oct	3
Gait analysis comparison of cruciate retaining and substituting TKA following PCL sacrifice	Joglekar S, Gioe TJ, Yoon P, Schwartz MH.	Knee. 2012 Aug	3
Early visual cues associated with a directional place kick in soccer	Lees A, Owens L.	Sports Biomech. 2011 Jun	3
Unanticipated ankle inversions are significantly different from anticipated ankle inversions during drop landings: overcoming anticipation bias	Dicus JR, Seegmiller JG.	J Appl Biomech. 2012 May	8
A feedback inclusive neuromuscular training program alters frontal plane kinematics	Greska EK, Cortes N, Van Lunen BL, Oñate JA.	J Strength Cond Res. 2012 Jun	3
Gait pattern classification in children with Charcot-Marie-Tooth disease type 1A	Ferrarin M, Bovi G, Rabuffetti M, Mazzoleni P, Montesano A, I	Gait Posture. 2012 Jan	3
The influence of shoe sole's varying thickness on lower limb muscle activity	Ramanathan AK, Parish EJ, Arnold GP, Drew TS, Wang W, A	Foot Ankle Surg. 2011 Dec	3
Landing technique affects knee loading and position during athletic tasks	Cortes N, Morrison S, Van Lunen BL, Onate JA.	J Sci Med Sport. 2012 Mar	5
Predictors of pelvic retraction in children with cerebral palsy derived from gait parameters and clinical testing	Böhm H, Stief F, Dussa CU, Döderlein L.	Gait Posture. 2012 Feb	3
Visualisation to enhance biomechanical tuning of ankle-foot orthoses (AFOs) in stroke: study protocol for a randomised controlled trial	Carse B, Bowers RJ, Meadows BC, Rowe PJ.	Trials. 2011 Dec 5	3
Ankle dorsiflexor function after plantar flexor surgery in children with cerebral palsy	Davids JR, Rogozinski BM, Hardin JW, Davis RB.	J Bone Joint Surg Am. 2011 Dec 7	3
A multi-segment foot model based on anatomically registered technical coordinate systems: method repeatability in pediatric feet	Saraswat P, MacWilliams BA, Davis RB.	Gait Posture. 2012 Apr	3
Whole body kinematics and knee moments that occur during an overhead catch and landing task in sport	Dempsey AR, Elliott BC, Munro BJ, Steele JR, Lloyd DG.	Clin Biomech (Bristol, Avon). 2012 Jun	3
Sensitivity of the OLGA and VCM models to erroneous marker placement: effects on 3D-gait kinematics	Groen BE, Geurts M, Nienhuis B, Duysens J.	Gait Posture. 2012 Mar	3
Varus ankle and osteochondral lesions of the talus	Easley ME, Vineyard JC.	Foot Ankle Clin. 2012 Mar	3
The varus ankle and instability	Klammer G, Benninger E, Espinosa N.	Foot Ankle Clin. 2012 Mar	3
Gait termination strategies differ between those with and without ankle instability	Wikstrom EA, Hass CJ.	Clin Biomech (Bristol, Avon). 2012 Jul	3
Ankle and midfoot kinetics during normal gait: a multi-segment approach	Dixon PC, Böhm H, Döderlein L.	J Biomech. 2012 Apr 5	3
Osteochondral lesions of the talus: effect of defect size and plantarflexion angle on ankle joint stresses	Hunt KJ, Lee AT, Lindsey DP, Slikker W 3rd, Chou LB.	Am J Sports Med. 2012 Apr	3
Lower extremity kinematics in children with and without flexible flatfoot: a comparative study	Shih YF, Chen CY, Chen WY, Lin HC.	BMC Musculoskelet Disord. 2012 Mar 2	3
Initial Achilles tendon repair strength--synthesized biomechanical data from 196 cadaver repairs	Sadoghi P, Rosso C, Valderrabano V, Leithner A, Vavken P.	Int Orthop. 2012 Sep	3
Leg adjustments during running across visible and camouflaged incidental changes in ground level	Müller R, Ernst M, Blickhan R.	J Exp Biol. 2012 Sep 1	3
Examining anticipatory turn signaling in typically developing 4- and 5-year-old children for applications in active orthotic devices	Stirling L, Weatherly J.	Gait Posture. 2013 Mar	3
Effect of fine wire electrode insertion on gait patterns in children with hemiplegic cerebral palsy	Krzak JJ, Corcos DM, Graf A, Smith P, Harris GF.	Gait Posture. 2013 Feb	3
A tale of two soles: sociomechanical and biomechanical considerations in diabetic limb salvage and amputation decision-making in the worst of times	Fiorito J, Trinidad-Hernadez M, Leykum B, Smith D, Mills JL, /	Diabet Foot Ankle. 2012	3
Impact of ankle muscle fatigue and recovery on the anticipatory postural adjustments to externally initiated perturbations in dynamic postural control	Kennedy A, Guevel A, Sveistrup H.	Exp Brain Res. 2012 Dec	3
			1
Posterior malleolus fracture	Irwin TA, Lien J, Kadakia AR.	J Am Acad Orthop Surg. 2013 Jan	3
Effects of visual information on perceived posture of an experimental phantom foot	Inui N, Masumoto J.	Exp Brain Res. 2013 May	3
Using decision analysis to assess comparative clinical efficacy of surgical treatment of unstable ankle fractures	Michelson JD.	J Orthop Trauma. 2013 Nov	3
A comprehensive evaluation of the variation in ankle function during gait in children and youth with Charcot-Marie-Tooth disease	Õunpuu S, Garibay E, Solomito M, Bell K, Pierz K, Thomson J	Gait Posture. 2013 Sep	3
Systematic review and evidence-based clinical recommendations for dosing of pediatric supported standing programs	Paleg GS, Smith BA, Glickman LB.	Pediatr Phys Ther. 2013 Fall	3
Electromyographic patterns of lower limb muscles during apprehensive gait in younger and older female adults	Hallal CZ, Marques NR, Spinoso DH, Vieira ER, Gonçalves M.	J Electromyogr Kinesiol. 2013 Oct	3
Differences in lateral drop jumps from an unknown height among individuals with functional ankle instability	Rosen A, Swanik C, Thomas S, Glutting J, Knight C, Kaminsk	J Athl Train. 2013 Nov-Dec	3
Effects of limiting anterior displacement of the center of foot pressure on anticipatory postural control during bilateral shoulder flexion	Fujiwara K, Yaguchi C.	J Electromyogr Kinesiol. 2013 Dec	3
[Arthrodesis and endoprostheses of the ankle joint: indications, techniques and pitfalls]	Wirth SH, Klammer G, Espinosa N.	Unfallchirurg. 2013 Sep	3
Reorganised anticipatory postural adjustments due to experimental lower extremity muscle pain	Shiozawa S, Hirata RP, Graven-Nielsen T.	Hum Mov Sci. 2013 Dec	3
Review of the evidence: surgical management of 4th and 5th tarsometatarsal joint osteoarthritis	Russell DF, Ferdinand RD.	Foot Ankle Surg. 2013 Dec	3
Modulation of anticipatory postural adjustments of gait using a portable powered ankle-foot orthosis	Petrucci MN, MacKinnon CD, Hsiao-Wecksler ET.	IEEE Int Conf Rehabil Robot. 2013 Jun	3
Spontaneous locomotor activity in late-stage chicken embryos is modified by stretch of leg muscles	Bradley NS, Ryu YU, Yeseta MC.	J Exp Biol. 2014 Mar 15	3
Control of dynamic foot-ground interactions in male and female soccer athletes: females exhibit reduced dexterity and higher limb stiffness during landing	Lyle MA, Valero-Cuevas FJ, Gregor RJ, Powers CM.	J Biomech. 2014 Jan 22	3
Impact of knee modeling approach on indicators and classification of anterior cruciate ligament injury risk	Robinson MA, Donnelly CJ, Tsao J, Vanrenterghem J.	Med Sci Sports Exerc. 2014 Jul	3
Lengthening scarf osteotomy for recurrent hallux valgus	Rose B, Bowman N, Edwards H, Rajaratnam SS, Armitage AF	Foot Ankle Surg. 2014 Mar	3
Effect of diabetic neuropathy severity classified by a fuzzy model in muscle dynamics during gait	Watari R, Sartor CD, Picon AP, Butugan MK, Amorim CF, Orte	J Neuroeng Rehabil. 2014 Feb 8	3
Tendon transfers-how do they work? Planning and implementation	Dowd T, Bluman EM.	Foot Ankle Clin. 2014 Mar	3
First metatarsocuneiform joint mobility: radiographic, anatomic, and clinical characteristics of the articular surface	Doty JF, Coughlin MJ, Hirose C, Stevens F, Schutt S, Kenned	Foot Ankle Int. 2014 May	3
Exercise and ankle sprain injuries: a comprehensive review	Calatayud J, Borreani S, Colado JC, Flandez J, Page P, Ande	Phys Sportsmed. 2014 Feb	3
Reduced StartReact effect and freezing of gait in Parkinson's disease: two of a kind?	Nonnekes J, Geurts AC, Nijhuis LB, van Geel K, Snijders AH,	J Neurol. 2014 May	3
Midsole thickness affects running patterns in habitual rearfoot strikers during a sustained run	TenBroek TM, Rodrigues PA, Frederick EC, Hamill J.	J Appl Biomech. 2014 Aug	3
Internal lengthening device for congenital femoral deficiency and fibular hemimelia	Shabtai L, Specht SC, Standard SC, Herzenberg JE.	Clin Orthop Relat Res. 2014 Dec	3
Impact of forearm fatigue on the postural response to an externally initiated, predictable perturbation	Kennedy A, Guevel A, Sveistrup H.	Eur J Appl Physiol. 2014	3
Effects of strengthening, stretching and functional training on foot function in patients with diabetic neuropathy: results of a randomized controlled trial	Sartor CD, Hasue RH, Cacciari LP, Butugan MK, Watari R, Pá	BMC Musculoskelet Disord. 2014 Apr 27	3
			1
Individuals with chronic ankle instability exhibit decreased postural sway while kicking in a single-leg stance	dos Santos MJ, Gorges AL, Rios JL.	Gait Posture. 2014	3
Contributions to the understanding of gait control	Simonsen EB.	Dan Med J. 2014 Apr	3
Dynamic knee joint mechanics after anterior cruciate ligament reconstruction	Clarke SB, Kenny IC, Harrison AJ.	Med Sci Sports Exerc. 2015 Jan	3
Influence of ankle loading on the relationship between temporal pressure and motor coordination during a whole-body paired task	Yiou E, Hussein T, LaRue J.	Exp Brain Res. 2014 Oct	3
Modifications in ankle dorsiflexor activation by applying a torque perturbation during walking in persons post-stroke: a case series	Blanchette AK, Noël M, Richards CL, Nadeau S, Bouyer LJ.	J Neuroeng Rehabil. 2014 Jun 9	3
Sensitivity of a subject-specific musculoskeletal model to the uncertainties on the joint axes location	Martelli S, Valente G, Viceconti M, Taddei F.	Comput Methods Biomech Biomed Engin. 2015	3
The midfoot is really deformed after hindfoot arthrodesis: how to salvage?	Fortin PT.	Foot Ankle Clin. 2014 Sep	3
Effects of amplitude cueing on postural responses and preparatory cortical activity of people with Parkinson disease	Smith BA, Jacobs JV, Horak FB.	J Neurol Phys Ther. 2014 Oct	3
Soldier-relevant loads impact lower limb biomechanics during anticipated and unanticipated single-leg cutting movements	Brown TN, O'Donovan M, Hasselquist L, Corner B, Schiffman	J Biomech. 2014 Nov 7	8
Age and muscle-dependent variations in corticospinal excitability during standing tasks	Remaud A, Bilodeau M, Tremblay F.	PLoS One. 2014 Oct 13	3
Proactive and reactive neuromuscular control in subjects with chronic ankle instability: evidence from a pilot study on landing	Levin O, Vanwanseele B, Thijsen JR, Helsen WF, Staes FF, D	Gait Posture. 2015 Jan	3
Changes in muscle activity in typically developing children walking with unilaterally induced equinus	Houx L, Lempereur M, Rémy-Néris O, Gross R, Brochard S.	Clin Biomech (Bristol, Avon). 2014 Dec	3
Anticipatory changes in control of swing foot and lower limb joints when walking onto a moving surface traveling at constant speed	Hsu WC, Wang TM, Lu HL, Lu TW.	Gait Posture. 2015 Jan	3
Use of smartphones and portable media devices for quantifying human movement characteristics of gait, tendon reflex response, and Parkinson's disease hand tremor	LeMoyne R, Mastroianni T.	Methods Mol Biol. 2015	3
Shoe collar height effect on athletic performance, ankle joint kinematics and kinetics during unanticipated maximum-effort side-cutting performance	Lam GW, Park EJ, Lee KK, Cheung JT.	J Sports Sci. 2015	5
WARRIOR-trial - is routine radiography following the 2-week initial follow-up in trauma patients with wrist and ankle fractures necessary: study protocol for a randomized controlled trial	Weil NL, Termaat MF, Rubinstein SM, El Moumni M, Zuidema	Trials. 2015 Feb 27	3
Type synthesis and preliminary design of devices supporting lower limb's rehabilitation	Olinski M, Lewandowski B, Gronowicz A.	Acta Bioeng Biomech. 2015	3
Gender Differences in Plantar Loading During an Unanticipated Side Cut on FieldTurf	Queen RM, Vap A, Moorman CT, Garrett WE Jr, Butler RJ.	Clin J Sport Med. 2016 Mar	5
Contribution of lower limb eccentric work and different step responses to balance recovery among older adults	Nagano H, Levinger P, Downie C, Hayes A, Begg R.	Gait Posture. 2015 Sep	3
The Relationship of Anticipatory Gluteus Medius Activity to Pelvic and Knee Stability in the Transition to Single-Leg Stance	Kim D, Unger J, Lanovaz JL, Oates AR.	PM R. 2016 Feb	3

Biomechanical Comparison of External Fixation and Compression Screws for Transverse Tarsal Joint Arthrodesis	Latt LD, Glisson RR, Adams SB Jr, Schuh R, Narron JA, Easl	Foot Ankle Int. 2015 Oct	3
Individuals with chronic ankle instability compensate for their ankle deficits using proximal musculature to maintain reduced postural sway while kicking a ball	Rios JL, Gorges AL, dos Santos MJ.	Hum Mov Sci. 2015 Oct	3
Comparative gait initiation kinematics between simulated unilateral and bilateral ankle hypomobility: Does bilateral constraint improve speed performance?	Delafontaine A, Honeine JL, Do MC, Gagey O, Chong RK.	Neurosci Lett. 2015 Aug 31	3
Ankle anticipatory postural adjustments during gait initiation in healthy and post-stroke subjects	Sousa AS, Silva A, Santos R.	Clin Biomech (Bristol, Avon). 2015 Nov	3
			1
Comparison of 2D-3D Measurements of Hallux and First Ray Sagittal Motion in Patients With and Without Hallux Valgus	Swanson JE, Stoltman MG, Oyen CR, Mohrbacher JA, Orand	Foot Ankle Int. 2016 Feb	3
Development of a functional anatomical subtalar pronator and supinator strength training machine	Hagen M, Lemke M, Kutsch HP, Lahner M.	Technol Health Care. 2015	3
Prospectively identified deficits in sagittal plane hip-ankle coordination in female athletes who sustain a second anterior cruciate ligament injury after anterior cruciate ligament reconstruction and return to sport	Paterno MV, Kiefer AW, Bonnette S, Riley MA, Schmitt LC, Fo	Clin Biomech (Bristol, Avon). 2015 Dec	3
Asymmetries in reactive and anticipatory balance control are of similar magnitude in Parkinson's disease patients	Boonstra TA, van Kordelaar J, Engelhart D, van Vugt JP, van	Gait Posture. 2016 Jan	3
Effectiveness and safety of prolotherapy injections for management of lower limb tendinopathy and fasciopathy: a systematic review	Sanderson LM, Bryant A.	J Foot Ankle Res. 2015 Oct 20	3
Motor Control of Landing from a Jump in Simulated Hypergravity	Gambelli CN, Theisen D, Willems PA, Schepens B.	PLoS One. 2015 Oct 27	3
Effects of ankle foot orthoses on body functions and activities in people with floppy paretic ankle muscles: a systematic review	van der Wilk D, Dijkstra PU, Postema K, Verkerke GJ, Hijman	Clin Biomech (Bristol, Avon). 2015 Dec	3
Acute Achilles Tendon Ruptures	Gross CE, Nunley JA 2nd.	Foot Ankle Int. 2016 Feb	3
Relationship between gait initiation and disability in individuals affected by multiple sclerosis	Galli M, Coghe G, Sanna P, Cocco E, Marrosu MG, Pau M.	Mult Scler Relat Disord. 2015 Nov	3
Cavovarus deformity in Charcot-Marie-Tooth disease: is there a hindfoot equinus deformity that needs treatment?	Beckmann NA, Wolf SI, Heitzmann D, Wallroth A, Müller S, D	J Foot Ankle Res. 2015 Nov 26	3
Variability of Anticipatory Postural Adjustments During Gait Initiation in Individuals With Parkinson Disease	Lin CC, Creath RA, Rogers MW.	J Neurol Phys Ther. 2016 Jan	3
Higher body mass index is associated with plantar fasciopathy/'plantar fasciitis': systematic review and meta-analysis of various clinical and imaging risk factors	van Leeuwen KD, Rogers J, Winzenberg T, van Middelkoop M	Br J Sports Med. 2016 Aug	3
Muscle activation patterns related to diabetic neuropathy in elderly subjects: A Functional Reach Test study	Maranesi E, Di Nardo F, Rabini RA, Ghetti GG, Burattini L, Me	Clin Biomech (Bristol, Avon). 2016 Feb	3
Age-Related Changes in Dynamic Postural Control and Attentional Demands are Minimally Affected by Local Muscle Fatigue	Remaud A, Thuong-Cong C, Bilodeau M.	Front Aging Neurosci. 2016 Jan 21	3
Event-related brain potential and postural muscle activity during standing on an oscillating table while the knee, hip, and trunk are fixed	Fujiwara K, Irei M, Kiyota N, Yaguchi C, Maeda K.	J Physiol Anthropol. 2016 Feb 18	3
Both anticipatory and compensatory postural adjustments are adapted while catching a ball in unstable standing posture	Scariot V, Rios JL, Claudino R, Dos Santos EC, Angulski HBE	J Bodyw Mov Ther. 2016 Jan	3
The Effect of Stimulus Timing on Unplanned Gait Termination	Ohm K, Hahn ME.	J Appl Biomech. 2016 Aug	3
lower limbs kinematic assessment of the effect of a gym and hydrotherapy rehabilitation protocol after knee megaprosthesis: a case report	Lovecchio N, Sciumè L, Zago M, Panella L, Lopresti M, Sforza	J Phys Ther Sci. 2016 Mar	3
Lower extremity muscle activation onset times during the transition from double-leg stance to single-leg stance in anterior cruciate ligament reconstructed subjects	Dingenen B, Janssens L, Claes S, Bellemans J, Staes FF.	Clin Biomech (Bristol, Avon). 2016 Jun	3
The impact of vision on the dynamic characteristics of the gait: strategies in children with blindness	Gazzellini S, Lispi ML, Castelli E, Trombetti A, Carniel S, Vasc	Exp Brain Res. 2016 Sep	3
The Influence of the Aquatic Environment on Gait Initiation: A Pilot Study	Marinho-Buzelli AR, Barela AM, Barela JA, Celestino ML, Pop	Motor Control. 2017 Apr	3
Preparation time influences ankle and knee joint control during dynamic change of direction movements	Fuerst P, Gollhofer A, Gehring D.	J Sports Sci. 2017 Apr	5
Changes in Postural Control After a Ball-Kicking Balance Exercise in Individuals With Chronic Ankle Instability	Conceição JS, Schaefer de Araújo FG, Santos GM, Keighley .	J Athl Train. 2016 Jun 2	3
Balance and recovery on coronally-uneven and unpredictable terrain	Yeates KH, Segal AD, Neptune RR, Klute GK.	J Biomech. 2016 Sep 6	3
Proceedings of the 3rd IPLeiria's International Health Congress : Leiria, Portugal. 6-7 May 2016	Tomás CC, Oliveira E, Sousa D, Uba-Chupel M, Furtado G, R	BMC Health Serv Res. 2016 Jul 6	3
Joint dynamics of rear- and fore-foot unplanned sidestepping	Donnelly CJ, Chinnasee C, Weir G, Sasimontonkul S, Alderso	J Sci Med Sport. 2017 Jan	5
Postural control and contingent negative variation during transient floor translation while standing with the ankle fixed	Lytnev V, Fujiwara K, Kiyota N, Irei M, Toyama H, Yaguchi C.	J Physiol Anthropol. 2016 Jul 25	3
Functional and Gait Assessment in Children and Adolescents Affected by Friedreich's Ataxia: A One-Year Longitudinal Study	Vasco G, Gazzellini S, Petrarca M, Lispi ML, Pisano A, Zazza	PLoS One. 2016 Sep 6	3
Calibration of the Leg Muscle Responses Elicited by Predictable Perturbations of Stance and the Effect of Vision	Sozzi S, Nardone A, Schieppati M.	Front Hum Neurosci. 2016 Aug 30	3
The Neuro-Mechanical Processes That Underlie Goal-Directed Medio-Lateral APA during Gait Initiation	Honeine JL, Schieppati M, Crisafulli O, Do MC.	Front Hum Neurosci. 2016 Aug 31	3
Distinct cut task strategy in Australian football players with a history of groin pain	Edwards S, Brooke HC, Cook JL.	Phys Ther Sport. 2017 Jan	5
Performance monitoring and response conflict resolution associated with choice stepping reaction tasks	Watanabe T, Tsutou K, Saito K, Ishida K, Tanabe S, Nojima I.	Exp Brain Res. 2016 Nov	3
Quantifying effects of age on balance and gait with inertial sensors in community-dwelling healthy adults	Park JH, Mancini M, Carlson-Kuhta P, Nutt JG, Horak FB.	Exp Gerontol. 2016 Dec 1	3
Biomechanical Effects of an Injury Prevention Program in Preadolescent Female Soccer Athletes	Thompson JA, Tran AA, Gatewood CT, Shultz R, Silder A, De	Am J Sports Med. 2017 Feb	3
Redistribution of Mechanical Work at the Knee and Ankle Joints During Fast Running in Minimalist Shoes	Fuller JT, Buckley JD, Tsiros MD, Brown NA, Thewlis D.	J Athl Train. 2016 Oct	3
The influence of high- and low-heeled shoes on balance in young women	Mika A, Oleksy Ł, Kielnar R, Świerczek M.	Acta Bioeng Biomech. 2016	3
Reliability of Visual Estimation of the First Intermetatarsal Angle	Higashi M, Shofler D, Manji K, Penera K.	J Foot Ankle Surg. 2017 Jan-Feb	3
Precise coding of ankle angle and velocity by human calf muscle spindles	Peters RM, Dalton BH, Blouin JS, Inglis JT.	Neuroscience. 2017 May 4	3
Center of pressure and center of mass behavior during gait initiation on inclined surfaces: A statistical parametric mapping analysis	Vieira MF, de Brito AA Junior, Lehnen GC, Rodrigues FB.	J Biomech. 2017 May 3	3
Stance foot alignment and hand positioning alter star excursion balance test scores in those with chronic ankle instability: What are we really assessing?	Cuğ M.	Physiother Theory Pract. 2017 Apr	3
Physiological arousal accompanying postural responses to external perturbations after stroke	Pollock CL, Carpenter MG, Hunt MA, Gallina A, Vieira TM, Iva	Clin Neurophysiol. 2017 Jun	3
Asymmetrical stabilization and mobilization exploited during static single leg stance and goal directed kicking	King AC, Wang Z.	Hum Mov Sci. 2017 Aug	3
Rigid Ankle Foot Orthosis Deteriorates Mediolateral Balance Control and Vertical Braking during Gait Initiation	Delafontaine A, Gagey O, Colnaghi S, Do MC, Honeine JL.	Front Hum Neurosci. 2017 Apr 28	3
Gait patterns of children and adolescents with Charcot-Marie-Tooth disease	Wojciechowski E, Sman A, Cornett K, Raymond J, Refshauge K, Menezes MP, Burns J		3
In-vivo analysis of ankle joint movement for patient-specific kinematic characterization	Ferraresi C, De Benedictis C, Franco W, Maffiodo D, Leardini	Proc Inst Mech Eng H. 2017 Sep	3
Functional and clinical outcomes of total ankle arthroplasty in elderly compared to younger patients	Tenenbaum S, Bariteau J, Coleman S, Brodsky J.	Foot Ankle Surg. 2017 Jun	3
Comparison of Multisegmental Foot and Ankle Motion Between Total Ankle Replacement and Ankle Arthrodesis in Adults	Seo SG, Kim EJ, Lee DJ, Bae KJ, Lee KM, Lee DY.	Foot Ankle Int. 2017 Sep	3
Effects of amplitude and predictability of perturbations to the arm on anticipatory and reactionary muscle responses to maintain balance	Forghani A, Preuss R, Milner TE.	J Electromyogr Kinesiol. 2017 Aug	3
			1
Electromyographic Pattern during Gait Initiation Differentiates Yoga Practitioners among Physically Active Older Subjects	Lelard T, Doutrellot PL, Temfemo A, Ahmaidi S.	Front Hum Neurosci. 2017 Jun 14	3
Standing on wedges modifies side-specific postural control in the presence of lateral external perturbations	Lee YJ, Liang JN, Chen B, Ganesan M, Aruin AS.	J Electromyogr Kinesiol. 2017 Oct	3
Gait characteristics of children and youth with chemotherapy induced peripheral neuropathy following treatment for acute lymphoblastic leukemia	Wright MJ, Twose DM, Gorter JW.	Gait Posture. 2017 Oct	3
Detecting the presence of leg length discrepancy based on gait deviations and functional measurement of leg length during walking	Khamis S, Leisman G, Carmeli E.	BMJ Case Rep. 2017 Aug 7	3
Transfer of improved movement technique after receiving verbal external focus and video instruction	Benjaminse A, Welling W, Otten B, Gokeler A.	Knee Surg Sports Traumatol Arthrosc. 2018 Mar	3
Functional significance of extent and timing of muscle activation during double poling on-snow with increasing speed	Zoppirolli C, Boccia G, Bortolan L, Schena F, Pellegrini B.	Eur J Appl Physiol. 2017 Nov	3
Preparatory cortical and spinal settings to counteract anticipated and non-anticipated perturbations	Wälchli M, Tokuno CD, Ruffieux J, Keller M, Taube W.	Neuroscience. 2017 Dec 4	8
Comparison of pre-contact joint kinematics and vertical impulse between vertical jump landings and step-off landings from equal heights	Harry JR, Freedman Silvernail J, Mercer JA, Dufek JS.	Hum Mov Sci. 2017 Dec	3
Lower limb biomechanical analysis during an unanticipated step on a bump reveals specific adaptations of walking on uneven terrains	Panizzolo FA, Lee S, Miyatake T, Rossi DM, Siviy C, Speecka	J Exp Biol. 2017 Nov 15	3
Finite element simulation on posterior tibial tendinopathy: Load transfer alteration and implications to the onset of pes planus	Wong DW, Wang Y, Leung AK, Yang M, Zhang M.	Clin Biomech (Bristol, Avon). 2018 Jan	3
Balance control during gait initiation: State-of-the-art and research perspectives	Yiou E, Caderby T, Delafontaine A, Fourcade P, Honeine JL.	World J Orthop. 2017 Nov 18	3
Effect of dual tasking on anticipatory and compensatory postural adjustments in response to external perturbations in individuals with nonspecific chronic low back pain: Electromyographic analysis	Hemmati L, Piroozi S, Rojhani-Shirazi Z.	J Back Musculoskelet Rehabil. 2018	3
Investigating the anticipatory postural adjustment phase of gait initiation in different directions in chronic ankle instability patients	Ebrahimabadi Z, Naimi SS, Rahimi A, Sadeghi H, Hosseini SM	J Bodyw Mov Ther. 2018 Jan	3
Abandonment of assistive products: assessing abandonment levels and factors that impact on it	Sugawara AT, Ramos VD, Alfieri FM, Battistella LR.	Disabil Rehabil Assist Technol. 2018 Oct	3
			1
Effect of experimental muscle pain on the acquisition and retention of locomotor adaptation: different motor strategies for a similar performance	Bouffard J, Salomoni SE, Mercier C, Tucker K, Roy JS, van de	J Neurophysiol. 2018 May 1	3
Gum acacia stabilized silver nanoparticles based nano-cargo for enhanced anti-arthritic potentials of hesperidin in adjuvant induced arthritic rats	Rao K, Aziz S, Roome T, Razzak A, Sikandar B, Jamali KS, Ir	Artif Cells Nanomed Biotechnol. 2018	3
Structural interaction between bone and implants due to arthroplasty of the first metatarsophalangeal joint	Martínez Bocanegra MA, Bayod Lopez J, Vidal-Lesso A, Bece	Foot Ankle Surg. 2019 Apr	3
Peak Lower Extremity Landing Kinematics in Dancers and Nondancers	Hansberger BL, Acocello S, Slater LV, Hart JM, Ambegaonkar	J Athl Train. 2018 Apr	3
The Influence of Circadian Variation on Etiological Markers of Ankle Injury	Brogden C, Marrin K, Page R, Greig M.	J Sport Rehabil. 2019 Jul 1	3
EMG-Torque Dynamics Change With Contraction Bandwidth	Golkar MA, Jalaleddini K, Kearney RE.	IEEE Trans Neural Syst Rehabil Eng. 2018 Apr	3
Voluntary Control of Residual Antagonistic Muscles in Transtibial Amputees: Feedforward Ballistic Contractions and Implications for Direct Neural Control of Powered Lower Limb Prostheses	Huang S, Huang H.	IEEE Trans Neural Syst Rehabil Eng. 2018 Apr	3
Physical Therapists' Use of Evaluation Measures to Inform the Prescription of Ankle-Foot Orthoses for Children with Cerebral Palsy	Kane KJ, Lanovaz JL, Musselman KE.	Phys Occup Ther Pediatr. 2019	3
Computation of hip rotation kinematics retrospectively using functional knee calibration during gait	Sangeux M.	Gait Posture. 2018 Jun	3
The impact of ankle-foot orthoses on toe clearance strategy in hemiparetic gait: a cross-sectional study	Pongpipatpaiboon K, Mukaino M, Matsuda F, Ohtsuka K, Tani	J Neuroeng Rehabil. 2018 May 23	3
Modified conventional gait model versus cluster tracking: Test-retest reliability, agreement and impact of inverse kinematics with joint constraints on kinematic and kinetic data	Mentiplay BF, Clark RA.	Gait Posture. 2018 Jul	3
Effect of different casting design characteristics on offloading the diabetic foot	Westra M, van Netten JJ, Manning HA, van Baal JG, Bus SA.	Gait Posture. 2018 Jul	3
A Biomechanical Comparison of Single-Leg Landing and Unplanned Sidestepping	Chinnasee C, Weir G, Sasimontonkul S, Alderson J, Donnelly	Int J Sports Med. 2018 Jul	8
Central nervous system modulates the neuromechanical delay in a broad range for the control of muscle force	Del Vecchio A, Úbeda A, Sartori M, Azorín JM, Felici F, Farina	J Appl Physiol (1985). 2018 Nov 1	3
An anatomically-based masking protocol for the assessment of in-shoe plantar pressure measurement of the forefoot	Forghany S, Bonanno DR, Menz HB, Landorf KB.	J Foot Ankle Res. 2018 Jun 15	3
External ankle taping does not alter lower extremity side-step cut and straight sprint biomechanics in young adult males	Moore C, Donovan L, Murray AM, Armstrong C, Glaviano NR.	Sports Biomech. 2020 Jun	3
Lower-Extremity Kinematics During Ankle Inversion Perturbations: A Novel Experimental Protocol That Simulates an Unexpected Lateral Ankle Sprain Mechanism	Simpson JD, Stewart EM, Mosby AM, Macias DM, Chander H	J Sport Rehabil. 2019 Aug 1	8
Young and older adults adapt automatic postural responses equivalently to repetitive perturbations but are unable to use predictive cueing to optimize recovery of balance stability	Coelho DB, Silva MB, de Lima-Pardini AC, Martinelli AR, da S	Neurosci Lett. 2018 Oct 15	3
Transitioning from the level surface to stairs in children with and without Down syndrome: Motor strategy and anticipatory locomotor adjustments	Liang H, Ke X, Wu J.	Gait Posture. 2018 Oct	3
Kinematic adaptation and changes in gait classification in running compared to walking in children with unilateral spastic cerebral palsy	Krätschmer R, Böhm H, Döderlein L.	Gait Posture. 2019 Jan	3
Biomechanical but not timed performance asymmetries persist between limbs 9 months after ACL reconstruction during planned and unplanned change of direction	King E, Richter C, Franklyn-Miller A, Daniels K, Wadey R, Jac	J Biomech. 2018 Nov 16	6
Multi-joint gait clustering for children and youth with diplegic cerebral palsy	Kuntze G, Nettel-Aguirre A, Ursulak G, Robu I, Bowal N, Gold	PLoS One. 2018 Oct 24	3
Understanding cutting maneuvers - The mechanical consequence of preparatory strategies and foot strike pattern	David S, Mundt M, Komnik I, Potthast W.	Hum Mov Sci. 2018 Dec	3
The influence of maximum isometric muscle force scaling on estimated muscle forces from musculoskeletal models of children with cerebral palsy	Kainz H, Goudriaan M, Falisse A, Huenaerts C, Desloovere K	Gait Posture. 2018 Sep	3
Descending stairs: Good or bad task to discriminate women with patellofemoral pain?	Novello AA, Garbelotti S Jr, Rabelo NDDA, Ferraz AN, Bley A	Gait Posture. 2018 Sep	3
Stimulus Prediction and Postural Reaction: Phase-Specific Modulation of Soleus H-Reflexes Is Related to Changes in Joint Kinematics and Segmental Strategy in Perturbed Upright Stance	Ritzmann R, Lee K, Krause A, Gollhofer A, Freyler K.	Front Integr Neurosci. 2018 Dec 19	3
Combining muscle morphology and neuromotor symptoms to explain abnormal gait at the ankle joint level in cerebral palsy	Schless SH, Cenni F, Bar-On L, Hanssen B, Goudriaan M, Pa	Gait Posture. 2019 Feb	3
Anticipation modulates neuromechanics of drop jumps in known or unknown ground stiffness	Helm M, Ritzmann R, Gollhofer A, Freyler K.	PLoS One. 2019 Jan 25	8
Neuromuscular control in individuals with chronic ankle instability: A comparison of unexpected and expected ankle inversion perturbations during a single leg drop-landing	Simpson JD, Stewart EM, Turner AJ, Macias DM, Wilson SJ, (	Hum Mov Sci. 2019 Apr	8
Neuromuscular dysfunction, independent of gait dysfunction, modulates trabecular bone homeostasis in mice	Bain SD, Huber P, Ausk BJ, Kwon RY, Gardiner EM, Srinivas	J Musculoskelet Neuronal Interact. 2019 Mar 1	3
Postural control of a musculoskeletal model against multidirectional support surface translations	Kaminishi K, Jiang P, Chiba R, Takakusaki K, Ota J.	PLoS One. 2019 Mar 6	3
Biomechanical Comparison of Low-Profile Contoured Locking Plate With Single Compression Screw to Fully Threaded Compression Screws for First MTP Fusion	Fuld RS 3rd, Kumparatana P, Kelley J, Anderson N, Baldini T,	Foot Ankle Int. 2019 Jul	3

Anticipating ankle inversion perturbations during a single-leg drop landing alters ankle joint and impact kinetics	Simpson JD, Stewart EM, Rendos NK, Cosio-Lima L, Wilson S	Hum Mov Sci. 2019 Aug	8
Clinical usefulness and challenges of instrumented motion analysis in patients with intellectual disabilities	Hallemans A, Van de Walle P, Wyers L, Verheyen K, Schoonj	Gait Posture. 2019 Jun	3
Quantifying clinical misinterpretations associated to one-segment kinetic foot modelling in both a healthy and patient population	Eerdekens M, Staes F, Matricali GA, Wuite S, Peerlinck K, De	Clin Biomech (Bristol, Avon). 2019 Jul	3
Effect of Different Insole Materials on Kinetic and Kinematic Variables of the Walking Gait in Healthy People	Özmanevra R, Angin S, Günal İH, Elvan A.	J Am Podiatr Med Assoc. 2018 Sep	3
Lack of Consensus on Return-to-Sport Criteria Following Lateral Ankle Sprain: A Systematic Review of Expert Opinions	Wikstrom EA, Mueller C, Cain MS.	J Sport Rehabil. 2020 Feb 1	3
Proportional Myoelectric Control of a Virtual Inverted Pendulum Using Residual Antagonistic Muscles: Toward Voluntary Postural Control	Fleming A, Huang S, Huang H.	IEEE Trans Neural Syst Rehabil Eng. 2019 Jul	3
Step down tests are the tasks that most differentiate the kinematics of women with patellofemoral pain compared to asymptomatic controls	Lopes Ferreira C, Barton G, Delgado Borges L, Dos Anjos Ra	Gait Posture. 2019 Jul	3
Modulation of anticipatory postural adjustments using a powered ankle orthosis in people with Parkinson's disease and freezing of gait	Petrucci MN, MacKinnon CD, Hsiao-Wecksler ET.	Gait Posture. 2019 Jul	3
Robust Longitudinal Ankle Edema Assessment Using Wearable Bioimpedance Spectroscopy	Mabrouk S, Hersek S, Jeong HK, Whittingslow D, Ganti VG, W	IEEE Trans Biomed Eng. 2020 Apr	3
The impact of multimorbidity on foot health outcomes in podiatry patients with musculoskeletal foot pain: a prospective observational study	Hendry GJ, Fenocchi L, Mason H, Steultjens M.	J Foot Ankle Res. 2019 Jul 3	3
			1
The relationship between leg stiffness, forces and neural control of the leg musculature during the stretch-shortening cycle is dependent on the anticipation of drop height	Helm M, Freyler K, Waldvogel J, Gollhofer A, Ritzmann R.	Eur J Appl Physiol. 2019 Sep	3
Proportional Myoelectric Control of a Powered Ankle Prosthesis for Postural Control under Expected Perturbation: A Pilot Study	Fleming A, Huang HH.	IEEE Int Conf Rehabil Robot. 2019 Jun	3
Utilizing three dimensional clinical gait analysis to optimize mobility outcomes in incomplete spinal cord damage	Murphy AT, Kravtsov S, Sangeux M, Rawicki B, New PW.	Gait Posture. 2019 Oct	3
Kinematic Analysis of Mae-Geri Kicks in Beginner and Advanced Kyokushin Karate Athletes	Błaszczyszyn M, Szczęsna A, Pawlyta M, Marszałek M, Karcz	Int J Environ Res Public Health. 2019 Aug 29	3
The Receptive and Propulsive Behavior of Human Foot Joints During Running With Different Striking Strategies	Deschamps K, Matricali G, Eerdekens M, Wuite S, Leardini A,	J Appl Biomech. 2019 Oct 1	3
Test-retest reliability and minimal detectable change of ankle kinematics and spatiotemporal parameters in MS population	Andreopoulou G, Mahad DJ, Mercer TH, van der Linden ML.	Gait Posture. 2019 Oct	3
Effect of shoe modifications on biomechanical changes in basketball: A systematic review	Lam WK, Kan WH, Chia JS, Kong PW.	Sports Biomech. 2022 May	3
Electromyographic and Safety Comparisons of Common Lower Limb Rehabilitation Exercises for People With Hemophilia	Calatayud J, Pérez-Alenda S, Carrasco JJ, Escriche-Escuder	Phys Ther. 2020 Jan 23	3
Lower extremity characteristics in recurrent clubfoot: Clinical and gait analysis findings that may influence decisions for additional surgery	Pierz KA, Lloyd JR, Solomito MJ, Mack P, Õunpuu S.	Gait Posture. 2020 Jan	3
Metatarsalgia: Assessment Algorithm and Decision Making	Lopez V, Slullitel G.	Foot Ankle Clin. 2019 Dec	3
Lower extremity kinematic analysis in male athletes with unilateral anterior cruciate reconstruction in a jump-landing task and its association with return to sport criteria	Norouzi S, Esfandiarpour F, Mehdizadeh S, Yousefzadeh NK,	BMC Musculoskelet Disord. 2019 Oct 27	3
Patient reported outcomes and ankle plantarflexor muscle performance following gastrocnemius recession for Achilles tendinopathy: A prospective case-control study	DiLiberto FE, Nawoczenski DA, Tome J, DiGiovanni BF.	Foot Ankle Surg. 2020 Oct	3
Surgical Biomechanics: Principles of Procedure Choice	Shapiro J.	Clin Podiatr Med Surg. 2020 Jan	3
Anticipatory coadaptation of ankle stiffness and sensorimotor gain for standing balance	Le Mouel C, Brette R.	PLoS Comput Biol. 2019 Nov 22	3
Surgical treatment of pes planovalgus in ambulatory children with cerebral palsy: Static and dynamic changes as characterized by multi-segment foot modeling, physical examination and radiographs	Nahm NJ, Sohrweide SS, Wervey RA, Schwartz MH, Novache	Gait Posture. 2020 Feb	3
Peroneus longus muscle exhibits pre-programmed anticipatory activity before unilateral abduction of the lower limb while standing: a pilot study	Tomita H, Nojima O, Sasahara T, Imaizumi F, Kanai A.	J Phys Ther Sci. 2019 Nov	3
Clustering classification of diabetic walking abnormalities: a new approach taking into account intralimb coordination patterns	Sawacha Z, Sartor CD, Yi LC, Guiotto A, Spolaor F, Sacco IC	Gait Posture. 2020 Jun	3
Validity and reliability of the Australian Therapy Outcome Measures - Physiotherapy, for podiatry (AusTOMs-PT for use in podiatry)	Williams CM, Davies N, Kolic J, Caserta A, James AM, Unswo	J Foot Ankle Res. 2020 Apr 25	3
Can kinematic and kinetic differences between planned and unplanned volleyball block jump-landings be associated with injury risk factors?	Mercado-Palomino E, Richards J, Molina-Molina A, Benítez JM	Gait Posture. 2020 Jun	3
Effects of metatarsal domes on plantar pressures in older people with a history of forefoot pain	Landorf KB, Ackland CA, Bonanno DR, Menz HB, Forghany S	J Foot Ankle Res. 2020 May 6	3
Influences of Lateral Jump Smash Actions in Different Situations on the Lower Extremity Load of Badminton Players	Hung CL, Hung MH, Chang CY, Wang HH, Ho CS, Lin KC.	J Sports Sci Med. 2020 May 1	3
Use and usability of custom-made dorsiflexion-restricting ankle-foot orthoses for calf muscle weakness in polio survivors: a cross-sectional survey	Ploeger HE, Bus SA, Brehm MA, Nollet F.	Eur J Phys Rehabil Med. 2020 Oct	3
Mechanical work performed by distal foot-ankle and proximal knee-hip segments during anticipated and unanticipated cutting	Liew BXW, Sullivan L, Morris S, Netto K.	J Biomech. 2020 Jun 9	8
A Nonproprietary Movement Analysis System (MoJoXlab) Based on Wearable Inertial Measurement Units Applicable to Healthy Participants and Those With Anterior Cruciate Ligament Reconstruction Across a	Islam R, Bennasar M, Nicholas K, Button K, Holland S, Mulhol	JMIR Mhealth Uhealth. 2020 Jun 16	3
Repeatability of plantar pressure assessment during barefoot walking in people with stroke	Rogers A, Morrison SC, Gorst T, Paton J, Freeman J, Marsde	J Foot Ankle Res. 2020 Jun 29	3
Center of pressure excursion and muscle activation during gait initiation in individuals with and without chronic ankle instability	Yousefi M, Sadeghi H, Ilbiegi S, Ebrahimabadi Z, Kakavand M	J Biomech. 2020 Jul 17	3
Field hockey sport-specific postures during unanticipated sidestepping: Implications for anterior cruciate ligament injury prevention	Smith M, Weir G, Donnelly CJ, Alderson J.	J Sports Sci. 2020 Nov	5
Instrumented triple single-leg hop test: A validated method for ambulatory measurement of ankle and knee angles using inertial sensors	Ahmadian N, Nazarahari M, Whittaker JL, Rouhani H.	Clin Biomech (Bristol, Avon). 2020 Dec	3
Ankle kinematics, center of pressure progression, and lower extremity muscle activity during a side-cutting task in participants with and without chronic ankle instability	Simpson JD, Koldenhoven RM, Wilson SJ, Stewart EM, Turne	J Electromyogr Kinesiol. 2020 Oct	3
Prescribing joint co-ordinates during model preparation in OpenSim improves lower limb unplanned sidestepping kinematics	Donnelly CJ, Jackson C, Weir G, Alderson J, Robinson MA.	J Sci Med Sport. 2021 Feb	5
Ankle Joint Pressure in Supination-External Rotation Injuries: A Biomechanical Study in an Unrestrained Cadaver Model	Fösel AC, Seidel A, Attinger MC, Zderic I, Gueorguiev B, Krau	Foot Ankle Spec. 2022 Aug	3
The development of a consensus statement for the prescription of powered wheelchair standing devices in Duchenne muscular dystrophy	Schofield C, Evans K, Young H, Paguinto SG, Carroll K, Town	Disabil Rehabil. 2022 May	3
Lower-Limb Biomechanical Characteristics Associated with Unplanned Gait Termination Under Different Walking Speeds	Zhou H, Cen X, Song Y, Ugbolue UC, Gu Y.	J Vis Exp. 2020 Aug 25	3
[External fixator: temporary fixation and soft tissue management of the ankle]	Kamin K, Rammelt S, Kleber C, Marx C, Schaser KD.	Oper Orthop Traumatol. 2020 Oct	3
Validity and reliability of smartphone use in assessing balance in patients with chronic ankle instability and healthy volunteers: A cross-sectional study	Abdo N, ALSaadawy B, Embaby E, Rehan Youssef A.	Gait Posture. 2020 Oct	3
Time-frequency analysis of muscle activation patterns in people with chronic ankle instability during Landing and cutting tasks	Kim H, Palmieri-Smith R, Kipp K.	Gait Posture. 2020 Oct	4
The coordination patterns of the foot segments in relation to lateral ankle sprain injury mechanism during unanticipated changes of direction	van der Merwe C, Shultz SP, Colborne GR, Hébert-Losier K, F	Foot (Edinb). 2020 Dec	5
Patient-Preferred Prosthetic Ankle-Foot Alignment for Ramps and Level-Ground Walking	Shepherd MK, Simon AM, Zisk J, Hargrove LJ.	IEEE Trans Neural Syst Rehabil Eng. 2021	3
Type of unanticipated stimulus affects lower extremity kinematics and kinetics during sidestepping	Schroeder LE, Peel SA, Leverenz BH, Weinhandl JT.	J Sports Sci. 2021 Mar	5
Sex-specific landing biomechanics and energy absorption during unanticipated single-leg drop-jumps in adolescents: implications for knee injury mechanics	Romanchuk NJ, Del Bel MJ, Benoit DL.	J Biomech. 2020 Dec 2	5
Biomechanics of lower limb in badminton lunge: a systematic scoping review	Lam WK, Wong DW, Lee WC.	PeerJ. 2020 Nov 4	3
Changes in the range of angular variation of the ankle, knee, hip and neck joints related to the awareness of an impending perturbation	Pawlack AR, Papcke C, Scheeren EM.	J Bodyw Mov Ther. 2020 Oct	3
The influence of sagittal trunk lean on uneven running mechanics	AminiAghdam S, Blickhan R, Karamanidis K.	J Exp Biol. 2021 Jan 6	3
Changing Perspectives: Offloading a Patient With a Diabetic Foot Ulcer as Opposed to Offloading a Diabetic Foot Ulcer	Samuelson KL, Kiefer CT, Wu SC, Crews RT.	Foot Ankle Spec. 2021 Aug	3
Ground reaction force complexity in hallux valgus	Farzadi M, Sanjari MA, Jalali M, Saeedi H, Kamali M, Movahe	Clin Biomech (Bristol, Avon). 2021 Jan	3
Angular Velocity, Moment, and Power Analysis of the Ankle, Knee, and Hip Joints in the Goalkeeper's Diving Save in Football	Ibrahim R, Kingma I, de Boode V, Faber GS, van Dieën JH.	Front Sports Act Living. 2020 Feb 28	3
Influence of a light touch reference on cutaneous reflexes from the hand during standing	Misiaszek JE, Hackett H, McMahon AJ, Krutz J.	Exp Brain Res. 2021 Mar	3
Age-Related Changes in Presynaptic Inhibition During Gait Initiation	Filho SS, Coelho DB, Ugrinowitsch C, de Souza CR, Magalhã	J Gerontol A Biol Sci Med Sci. 2021 Mar 31	3
Using 3-Dimensional Motion Analysis to Optimize Treatment Planning for a Patient With Dropfoot: Case Report	Feng J, Bompiani E, Coats S, Patrick Do K, Aiona M.	Phys Ther. 2021 Mar 3	3
[Fracture of the posterior malleolus : A paradigm shift]	Mittlmeier T, Saß M, Randow M, Wichelhaus A.	Unfallchirurg. 2021 Mar	3
Paired nonlinear behavior of active and passive joint torques associated with preparation for walk-to-run gait transition	Pan J, Zhang S, Li L.	J Electromyogr Kinesiol. 2021 Apr	3
Peak Forces and Force Generating Capacities of Lower Extremity Muscles During Dynamic Tasks in People With and Without Chronic Ankle Instability	Kim H, Palmieri-Smith R, Kipp K.	Sports Biomech. 2022 Apr	4
Trunk, pelvis and lower limb coordination between anticipated and unanticipated sidestep cutting in females	Dutaillis B, Opar DA, Pataky T, Timmins RG, Hickey JT, Mani	Gait Posture. 2021 Mar	1
Which jump-landing task best represents lower extremity and trunk kinematics of unanticipated cutting maneuver?	Hanzlíková I, Richards J, Athens J, Hébert-Losier K.	Gait Posture. 2021 Mar	8
A Field-Based Approach to Determine Soft Tissue Injury Risk in Elite Futsal Using Novel Machine Learning Techniques	Ruiz-Pérez I, López-Valenciano A, Hernández-Sánchez S, Pu	Front Psychol. 2021 Feb 5	3
Coronal Lateral Collateral Ligament Sign: A Novel Magnetic Resonance Imaging Sign for Identifying Anterior Cruciate Ligament-Deficient Knees in Adolescents and Summarizing the Extent of Anterior Tibial Tr	Mitchell BC, Siow MY, Bastrom T, Bomar JD, Pennock AT, Pa	Am J Sports Med. 2021 Mar	3
The Utility of Functional Data Analyses to Reveal Between-Limbs Asymmetries in Those With a History of Anterior Cruciate Ligament Reconstruction	White MS, Horton WZ, Burland JP, Seeley MK, Lepley LK.	J Athl Train. 2021 Mar 1	3
Postural adjustments impairments in elderly people with chronic low back pain	Garcez DR, da Silva Almeida GC, Silva CFO, de Souza Nasci	Sci Rep. 2021 Feb 26	3
A Clinical Practice Guideline for the Use of Ankle-Foot Orthoses and Functional Electrical Stimulation Post-Stroke	Johnston TE, Keller S, Denzer-Weiler C, Brown L.	J Neurol Phys Ther. 2021 Apr 1	3
The Influence of Asymptomatic Hypermobility on Unanticipated Cutting Biomechanics	Hanzlíková I, Richards J, Athens J, Hébert-Losier K.	Sports Health. 2021 Nov-Dec	3
Stepping onto the unknown: reflexes of the foot and ankle while stepping with perturbed perceptions of terrain	Riddick RC, Farris DJ, Cresswell AG, Kuo AD, Kelly LA.	J R Soc Interface. 2021 Mar	3
Understanding the impact of lumbar disc degeneration and chronic low back pain: A cross-sectional electromyographic analysis of postural strategy during predicted and unpredicted postural perturbations	Deane JA, Lim AKP, McGregor AH, Strutton PH.	PLoS One. 2021 Apr 1	3
Lower extremity joint kinematics of a simulated lateral ankle sprain after drop landings in participants with chronic ankle instability	Simpson JD, Koldenhoven RM, Wilson SJ, Stewart EM, Turne	Sports Biomech. 2022 Apr	8
Mechanisms of postural control in older adults based on surface electromyography data	Kasahara S, Saito H.	Hum Mov Sci. 2021 Aug	3
Podiatrists' views and experiences of using real time clinical gait analysis in the assessment and treatment of posterior tibial tendon dysfunction	Harradine P, Gates L, Metcalf C, Bowen C.	J Foot Ankle Res. 2021 Jun 4	3
Muscle force contributions to ankle joint contact forces during an unanticipated cutting task in people with chronic ankle instability	Kim H, Palmieri-Smith R, Kipp K.	J Biomech. 2021 Jul 19	5
Effect of Static Alignment on Dynamic Knee Abduction Moments in Adolescent Athletes with Recent ACL Reconstruction	Mueske NM, Abousamra O, Katzel MJ, Vandenberg CD, Pace	Med Sci Sports Exerc. 2021 Aug 1	3
A Neural Network Estimation of Ankle Torques From Electromyography and Accelerometry	Siu HC, Sloboda J, McKindles RJ, Stirling LA.	IEEE Trans Neural Syst Rehabil Eng. 2021	3
Effects of ankle-foot orthoses on the stability of post-stroke hemiparetic gait	Tsuchiyama K, Mukaino M, Ohtsuka K, Matsuda F, Tanikawa	Eur J Phys Rehabil Med. 2022 Jun	3
Forefoot adduction and forefoot supination as kinematic indicators of relapse clubfoot	Grin L, van der Steen MC, Wijnands SDN, van Oorschot L, Be	Gait Posture. 2021 Oct	3
Functional assessment of stretch hyperreflexia in children with cerebral palsy using treadmill perturbations	Flux E, van der Krogt MM, Harlaar J, Buizer AI, Sloot LH.	J Neuroeng Rehabil. 2021 Oct 18	3
A Novel Viewpoint on the Anticipatory Postural Adjustments During Gait Initiation	Farinelli V, Bolzoni F, Marchese SM, Esposti R, Cavallari P.	Front Hum Neurosci. 2021 Oct 11	3
Visualization and quantification of the degenerative pattern of the distal tibia and fibula in unilateral varus ankle osteoarthritis	Seki H, Ogihara N, Kokubo T, Nagura T.	Sci Rep. 2021 Nov 3	3
Kinematic effects of different gait speeds during gait initiation movement	Sudo D, Hosokawa M, Maeda Y.	J Phys Ther Sci. 2021 Nov	3
Muscle Synergies in People With Chronic Ankle Instability During Anticipated and Unanticipated Landing-Cutting Tasks	Kim H, Palmieri-Smith R, Kipp K.	J Athl Train. 2023 Feb 1	4
Biomechanics Analysis of the Lower Limbs in 20 Male Sprinters Using the International Society of Biomechanics Six-Degrees-of-Freedom Model and the Conventional Gait Model	Ji Y, Xu R, Zuo H, Wang Z, Jin H.	Med Sci Monit. 2021 Nov 26	3
Arch-Support Induced Changes in Foot-Ankle Coordination in Young Males with Flatfoot during Unplanned Gait Termination	Cen X, Gao L, Yang M, Liang M, Bíró I, Gu Y.	J Clin Med. 2021 Nov 26	3
Clusterization of multi-channel electromyograms into muscle-specific activations to drive a subject-specific musculoskeletal model: towards fast and accurate clinical decision-making	Simonetti D, Koopman BFJM, Sartori M.	Annu Int Conf IEEE Eng Med Biol Soc. 2021 Nov	3
Posterior tibial tendon dysfunction alters the midfoot mechanics and energetics during gait	Wuite S, Deschamps K, Eerdekens M, Scheys L, Loomans L,	J Orthop Res. 2022 Sep	3
Copers adopt an altered dynamic postural control compared to individuals with chronic ankle instability and controls in unanticipated single-leg landing	Watabe T, Takabayashi T, Tokunaga Y, Kubo M.	Gait Posture. 2022 Feb	4
What are the long-term outcomes of lateral column lengthening for pes planovalgus in cerebral palsy?	Kruger KM, Constantino CS, Graf A, Flanagan A, Smith PA, K	J Clin Orthop Trauma. 2021 Nov 26	3
Continuous similarity analysis in patient populations	Hill CN, Ross S, Peebles A, Queen RM.	J Biomech. 2022 Jan	3
A randomized crossover study of functional electrical stimulation during walking in spastic cerebral palsy: the FES on participation (FESPa) trial	Moll I, Marcellis RGJ, Coenen MLP, Fleuren SM, Willems PJB	BMC Pediatr. 2022 Jan 13	3
The effect of interventions anticipated to improve plantar intrinsic foot muscle strength on fall-related dynamic function in adults: a systematic review	Willemse L, Wouters EJM, Bronts HM, Pisters MF, Vanwanse	J Foot Ankle Res. 2022 Jan 20	3
The importance of joint line obliquity: a radiological analysis of restricted boundaries in normal knee phenotypes to inform surgical decision making in kinematically aligned total knee arthroplasty	MacDessi SJ, Allom RJ, Griffiths-Jones W, Chen DB, Wood J	Knee Surg Sports Traumatol Arthrosc. 2022 Sep	3
Is subject-specific musculoskeletal modelling worth the extra effort or is generic modelling worth the shortcut?	Akhundov R, Saxby DJ, Diamond LE, Edwards S, Clausen P,	PLoS One. 2022 Jan 25	3
Influence of Landing in Neuromuscular Control and Ground Reaction Force with Ankle Instability: A Narrative Review	Lin JZ, Lin YA, Tai WH, Chen CY.	Bioengineering (Basel). 2022 Feb 10	3
Predicting biological joint moment during multiple ambulation tasks	Camargo J, Molinaro D, Young A.	J Biomech. 2022 Mar	3
Are Clinical Impairments Related to Kinematic Gait Variability in Children and Young Adults With Cerebral Palsy?	Tabard-Fougère A, Rutz D, Pouliot-Laforte A, De Coulon G, N	Front Hum Neurosci. 2022 Mar 2	3
Arthroplasty Surgeons Differ in Their Intraoperative Soft Tissue Assessments: A Study in Human Cadavers to Quantify Surgical Decision-making in TKA	Elmasry SS, Sculco PK, Kahlenberg CA, Mayman DJ, Cross M	Clin Orthop Relat Res. 2022 Aug 1	3

	Comparison of Azure Kinect and optical retroreflective motion capture for kinematic and spatiotemporal evaluation of the sit-to-stand test	Thomas J, Hall JB, Bliss R, Guess TM.	Gait Posture. 2022 May	3
	Comparing lab and field agility kinematics in young talented female football players: Implications for ACL injury prevention	Di Paolo S, Nijmeijer E, Bragonzoni L, Dingshoff E, Gokeler A	Eur J Sport Sci. 2023 May	5
	Effect of coronal fracture angle on the stability of screw fixation in medial malleolar fractures: A finite element analysis	Yunus Emre T, Kursat Celik H, Arik HO, Rennie AEW, Kose O	Proc Inst Mech Eng H. 2022 Jun	3
	Identification of Hip and Knee Joint Impedance During the Swing Phase of Walking	van der Kooij H, Fricke SS, Veld RCV, Prieto AV, Keemink AC	IEEE Trans Neural Syst Rehabil Eng. 2022	3
	Significant variations in surgical construct and return to sport protocols with syndesmotic injuries: an ISAKOS global perspective	Hunt KJ, Bartolomei J, Challa SC, McCormick JJ, D'Hooghe P, Tuffiash M, Amendola A		3
	Comparison of Azure Kinect overground gait spatiotemporal parameters to marker based optical motion capture	Guess TM, Bliss R, Hall JB, Kiselica AM.	Gait Posture. 2022 Jul	3
	Kinematics predictors of spatiotemporal parameters during gait differ by age in healthy individuals	de Campos DDSF, Shokur S, de Lima-Pardini AC, Runfeng M	Gait Posture. 2022 Jul	3
	kinesio taping	Russell Esposito E, Farrokhi S, Shuman BR, Sessoms PH, Sz	JMIR Res Protoc. 2022 Jun 22	3
	Intraoperative Assessment of Reduction of the Ankle Syndesmosis	Hao KA, Vander Griend RA, Nichols JA, Reb CW.	Curr Rev Musculoskelet Med. 2022 Oct	3
	Women's College Volleyball Players Exhibit Asymmetries During Double-Leg Jump Landing Tasks	Taylor JB, Nguyen AD, Westbrook AE, Trzeciak A, Ford KR.	J Sport Rehabil. 2022 Aug 8	8
	Surgical Fixation of Calcaneal Beak Fractures-Biomechanical Analysis of Different Osteosynthesis Techniques	Jordan MC, Hufnagel L, McDonogh M, Paul MM, Schmalzl J, I	Front Bioeng Biotechnol. 2022 Aug 4	3
	Kinematic alignment in total knee arthroplasty: a five-year prospective, multicentre, survivorship study	Tran T, McEwen P, Peng Y, Trivett A, Steele R, Donnelly W, (	Bone Jt Open. 2022 Aug	3
	Data-driven Gait-predictive Model for Anticipatory Prosthesis Control	Dey S, Schilling AF.	IEEE Int Conf Rehabil Robot. 2022 Jul	3
	Parents, health professionals and footwear stakeholders' beliefs on the importance of different features of young children's footwear: a qualitative study	Williams CM, Banwell HA, Paterson KL, Gobbi K, Burton S, Hi	J Foot Ankle Res. 2022 Oct 12	3
	Evaluation of assumptions in foot and ankle biomechanical models	Malakoutikhah H, de Cesar Netto C, Madenci E, Latt LD.	Clin Biomech (Bristol, Avon). 2022 Dec	3
	The medium-term effects of selective dorsal rhizotomy on gait compared to a matched cerebral palsy non-SDR group: A follow-up study	Marron A, O'Sullivan R, Leonard J, Kiernan D.	Gait Posture. 2023 Jan	3
	The Effect of Arch Stiffness on the Foot-Ankle Temporal Kinematics during Gait Termination: A Statistical Nonparametric Mapping Study	Cen X, Yu P, Song Y, Sárosi J, Mao Z, Bíró I, Gu Y.	Bioengineering (Basel). 2022 Nov 17	3
	Knee adduction moment decomposition: Toward better clinical decision-making	Baniasad M, Martin R, Crevoisier X, Pichonnaz C, Becce F, A	Front Bioeng Biotechnol. 2022 Nov 18	3
	Radiographic Evaluation of Isolated Continuous Compression Staples for Akin Osteotomy Fixation	Horner K, Summerhays B, Fiala K, Schweser KM.	J Foot Ankle Surg. 2023 May-Jun	3
	Pedobarographic evaluation of five commonly used orthoses for the lower extremity	Ehrnthaller C, Rellensmann K, Baumbach SF, Wuehr M, Schr	Arch Orthop Trauma Surg. 2023 Jul	3
	No Difference in Risk of Amputation or Frequency of Surgical Interventions Between Patients With Diabetic and Nondiabetic Charcot Arthropathy	Waibel FWA, Weber S, Selman F, Götschi T, Berli MC, Böni T	Clin Orthop Relat Res. 2023 Aug 1	3
	Use of fluorescence imaging to optimize location of tissue sampling in hard-to-heal wounds	Serena TE, Snyder RJ, Bowler PG.	Front Cell Infect Microbiol. 2023 Jan 12	3
	New Classification Based on CT and Its Value Evaluation for Fractures of the Lateral Process of the Talus	Wang Y, Wang Z, Zhu Y, Fu L, Deng X, Chen W, Zhang Y.	J Foot Ankle Surg. 2023 Jul-Aug	3
	A 3-DoF robotic platform for the rehabilitation and assessment of reaction time and balance skills of MS patients	Ersoy T, Hocaoglu E.	PLoS One. 2023 Feb 24	3
	Biomechanical Analysis of Unplanned Gait Termination According to a Stop-Signal Task Performance: A Preliminary Study	Koo DK, Kwon JW.	Brain Sci. 2023 Feb 10	3
	Effects of anticipation on joint kinematics during inversion perturbation in individuals with chronic ankle instability	Han S, Lee H, Hopkins JT.	Scand J Med Sci Sports. 2023 Jul	8
	Bone marrow stimulation in arthroscopic rotator cuff repair is a cost-effective and straightforward technique to reduce retear rates: A systematic review and meta-analysis	Zhang L, Zhu Y, Xu T, Fu W.	Front Surg. 2023 Feb 21	3
	Methods for Biomechanical Testing of Posterior Malleolar Fractures in Ankle Fractures: A Scoping Review	Stake IK, Douglass BW, Husebye EE, Clanton TO.	Foot Ankle Int. 2023 Apr	3
	Ankle Kinematics Characterization in Children with Idiopathic Toe Walking: Does the Foot Model Change the Clinical Evaluation?	Brasiliano P, Alvini M, Di Stanislao E, Vannozzi G, Di Rosa G,	Healthcare (Basel). 2023 Mar 16	3
	Effects of mental fatigue on biomechanical characteristics of lower extremities in patients with functional ankle instability during unanticipated side-step cutting	Kong L, Wu P, Zhang X, Meng L, Kong L, Zhang Q, Shen J.	Front Physiol. 2023 Mar 23	6
	Perspectives on ankle-foot technology for improving gait performance of children with Cerebral Palsy in daily-life: requirements, needs and wishes	Bayón C, Hoorn MV, Barrientos A, Rocon E, Trost JP, Asseld	J Neuroeng Rehabil. 2023 Apr 12	3
	The effect of a startle-based warning, age, sex, and secondary task on takeover actions in critical autonomous driving scenarios	Griffith M, Akkem R, Maheshwari J, Seacrist T, Arbogast KB, (	Front Bioeng Biotechnol. 2023 Mar 27	3
	A combined FE-hybrid MCDM framework for improving the performance of the conical stem tibial design for TAR with the addition of pegs	Jyoti, Ghosh R.	Comput Methods Programs Biomed. 2023 Jul	3
	Voluntary muscle coactivation in quiet standing elicits reciprocal rather than coactive agonist-antagonist control of reactive balance	Martino G, Beck ON, Ting LH.	J Neurophysiol. 2023 Jun 1	3
	The Influence of Experience on Neuromuscular Control of the Body When Cutting at Different Angles	Pan Z, Liu L, Li X, Ma Y.	J Mot Behav. 2023	3
	Effect of the COVID-19 Pandemic on Lower Extremity Injuries in Japanese Collegiate Men's Basketball Players	Sekine Y, Kamada K, Koyama T, Hoshikawa S, Ito E, Uchino	Orthop J Sports Med. 2023 May 30	3
	Machine learning-based prediction of joint moments based on kinematics in patients with cerebral palsy	Ozates ME, Karabulut D, Salami F, Wolf SI, Arslan YZ.	J Biomech. 2023 Jun	3
	The Association of Age and Sex With Joint Angles and Coordination During Unanticipated Cutting in Soccer Players	Robbins SM, Lopes Lima Y, Brown H, Morelli M, Pearsall DJ,	Motor Control. 2023 Jun 8	5
	Runners with a history of shank and foot injury: Interactions among local musculoskeletal factors, age, and running experience	Frederico RA, Santos TRT, Okai-Nóbrega LA, Ocarino JM, Sc	Phys Ther Sport. 2023 Jul	3
	Decision-Making and Management of Tarsal Coalition in the Young Adult Patient: A Critical Analysis Review	Catanzano AA Jr, Akoh CC, Easley ME, Mosca VS.	JBJS Rev. 2023 Jun 12	3
	Fractures and dislocations of the foot and ankle in people with diabetes: a literature review	Johnson MJ, Kandasamy S, Raspovic KM, Manchanda K, Liu	Ther Adv Endocrinol Metab. 2023 Jun 3	3
	Peroneal muscle response to single-leg drop-jump and unexpected leg-drop in young and middle-aged adults before and after one session of neuromuscular training	Hayek R, Gottlieb U, Gutman I, Springer S.	Eur Rev Aging Phys Act. 2023 Jun 17	3
	An Experimental Approach to Induce Trips in Lower-Limb Amputees	Rodacki ALF, Buckley JG, Passos de Oliveira AC, Marçal da S	J Vis Exp. 2023 Sep 22	3
	Neural prosthesis control restores near-normative neuromechanics in standing postural control	Fleming A, Liu W, Huang HH.	Sci Robot. 2023 Oct 25	3
	High Prevalence of Work-related Musculoskeletal Disorders and Limited Evidence-based Ergonomics in Orthopaedic Surgery: A Systematic Review.	Vasireddi N, Vasireddi N, Shah AK, Moyal AJ, Gausden EB, M	Clin Orthop Relat Res., 2023 Nov 28	3
	Effect of Chronic Ankle Instability on the Biomechanical Organization of Gait Initiation: A Systematic Review.	Yousefi M, Zivari S, Yiou E, Caderby T.	Brain Sci. 2023 Nov 17;	3
	Lower-extremity kinematics and kinetics differ based on drop vertical jump variation: An assessment of methodology for a return-to-play protocol using motion analysis.	Ulman S, Loewen AM, Erdman AL, Õunpuu S, Chafetz R, Tul	Gait Posture. 2023 Nov 28	3
	Biomechanical insights into ankle instability: a finite element analysis of posterior malleolus fractures.	Ying J, Liu J, Wang H, Zhuang Y, Yu T, Wang S, Huang D.	J Orthop Surg Res. 2023 Dec 12;18(1):957	3
	From data to action: a scoping review of wearable technologies and biomechanical assessments informing injury prevention strategies in sport.	Rebelo A, Martinho DV, Valente-Dos-Santos J, Coelho-E-Silva	BMC Sports Sci Med Rehabil. 2023 Dec 14;15(1):169	3
	How mechanics of individual muscle-tendon units define knee and ankle joint function in health and cerebral palsy-a narrative review.	Kaya Keles CS, Ates F.	Front Bioeng Biotechnol. 2023 Dec 5;11:1287385	3
	Lack of Proprioceptive Strategy Modulation Leads to At-Risk Biomechanics for Anterior Cruciate Ligament in Healthy Athletes.	Picot B, Lempereur M, Morel B, Forestier N, Rémy-Néris O.	Med Sci Sports Exerc. 2024 Jan 8	3
	Perceptions and experiences of first mobility aid provision for young children with cerebral palsy in the United States: a mixed-methods study.	Zaino NL, McKee Z, Caskey CD, Steele KM, Feldner HA	Disabil Rehabil Assist Technol. 2024 Feb 12:1-12.	3
				1
	Gender-based differences exist in the functional knee phenotypes classification of the osteoarthritic knee.	Chelli S, Rudyy T, Avram GM, Huegli RW, Amsler F, Hirschma	Knee Surg Sports Traumatol Arthrosc. 2024 Feb 28.	3
	Effect of unpredictable timing on the hip, knee, and ankle kinematics and center of mass during deceleration tasks.	Aoki A, Tamura T, Hoshi K, Gamada K.	J Sports Med Phys Fitness. 2024 Mar 6.	3
	A Data-Driven Approach to Estimate Human Center of Mass State During Perturbed Locomotion Using Simulated Wearable Sensors.	Leestma JK, Smith CR, Sawicki GS, Young AJ.	Ann Biomed Eng. 2024 Aug;52(8):2013-2023	3
	Innovative Design and Development of Personalized Ankle-Foot Orthoses for Survivors of Stroke With Equinovarus Foot: Protocol for a Feasibility and Comparative Trial.	Silva R, Morouço P, Lains J, Amorim P, Alves N, Veloso AP	JMIR Res Protoc. 2024 Apr 2;13:e52365.	3
	Multi-segment foot kinematics during gait in children with spastic cerebral palsy.	Schallig W, Piening M, Quirijnen L, Witbreuk MM, Buizer AI, va	Gait Posture. 2024 May;110:144-149. doi: 10.1016/j.gaitpost.2024.03.014.	3
	Biomechanics of the Human Osteochondral Unit: A Systematic Review.	Berni M, Marchiori G, Baleani M, Giavaresi G, Lopomo NF.	Materials (Basel). 2024 Apr 8;17(7):1698.	3
	Share Effects of Combining Transcranial Direct Current Stimulation With Balance Training on Anticipatory Postural Adjustments in Persons With Chronic Ankle Instability.	Beyraghi Z, Khanmohammadi R, Hadian MR.	Sports Health. 2024 May 8:19417381241247746.	3
	Neuromuscular and trunk control mediate factors associated with injury in fatigued runners.	Glover NA, Chaudhari AM,	J Biomech. 2024 Jun;170:112176. doi: 10.1016/j.jbiomech.2024.112176.	3
	Effects of ankle Kinesio taping on knee and ankle joint biomechanics during unanticipated jumps in collegiate athletes	Liu Q, Wang L, Dai F, Wang G, Chen P.	PLoS One. 2024 Aug 1;19(8):e0305480.	5
	A proposed evidence-guided algorithm for the adjustment and optimization of multi-function articulated ankle-foot orthoses in the clinical setting.	LeCursi NA, Janka BM, Gao F, Orendurff MS, He Y, Kobayasl	Front Rehabil Sci. 2024 Jul 24;5:1353303.	3
	Reduction in preparatory brain activity preceding gait initiation in individuals with chronic ankle instability: A movement-related cortical potential study.	Beyraghi Z, Khanmohammadi R, Hadian MR.	Eur J Neurosci. 2024 Sep;60(6):5284-5299.	3
	Design modification and selection of improved stem design of the conical stem tibial implant for TAR using FE analysis and different MCDM methods.	Jyoti, Ghosh R.	Proc Inst Mech Eng H. 2024 Aug-Sep;238(8-9):874-885.	3
	The relationship between executed cut angle and speed with lower extremity joint angles during unanticipated side-step cutting in soccer players	Robbins SM, Brown H, Lima YL, Morelli M, Pearsall DJ, Lamo	Gait Posture. 2024 Oct;114:84-89.	5
				1
	Rehabilitation for ankle fractures in adults.	Lewis SR, Pritchard MW, Parker R, Searle HKC, Beckenkamp	Cochrane Database Syst Rev. 2024 Sep 23;9(9):CD005595.	3
	A clinical investigation of force plate drift error on predicted joint kinetics during gait.	Milnes J, Kiernan D.	J Biomech. 2024 Nov;176:112351.	3
	Can Foot Orthoses Benefit Symptomatic Runners? Mechanistic and Clinical Insights Through a Scoping Revi	Del Duchetto F, Dussault-Picard C, Gagnon M, Dixon P, Cher	Sports Med Open. 2024 Oct 4;10(1):108.	3
	Evidence of invariant lower-limb kinematics in anticipation of ground contact during drop-landing and drop-jumping.	Bechet R, Tisserand R, Fradet L, Colloud F.	Hum Mov Sci. 2024 Dec;98:103297.	3
	Effect of Unanticipated Tasks on Side-Cutting Stability of Lower Extremity with Patellofemoral Pain Syndrome	Ma Y, Quan W, Wang X, Baker JS, Gao Z, Gu Y.	Sensors (Basel). 2024 Oct 4;24(19):6427.	6
	Decreased proprioception is associated with inferior postural control during unplanned landing in individuals with chronic ankle instability	Hou Z, Fong DTP, Winter SL.	J Sports Sci. 2024 Oct;42(20):1932-1938.	4
	Evaluation of drop vertical jump kinematics and kinetics using 3D markerless motion capture in a large cohort	Templin T, Riehm CD, Eliason T, Hulburt TC, Kwak ST, Medja	Front Bioeng Biotechnol. 2024 Oct 24;12:1426677.	3
	Does chronic ankle instability affect side-cutting in female soccer players?	Takeuchi S, Anan M.	Int J Sports Med . 2024 Nov 25	5
	Biomechanic Differences Between Anticipated and Unanticipated Volleyball Block Jump: Implications for Lower Limb Injury Risk.	Zhao H, Liu X, Dan L, Xu D, Li J.	Life (Basel). 2024 Oct 23;14(11):1357.	3
	Modern anatomical locking plates are associated with increased postoperative wound complications and unplanned surgical revisions compared to standard tubular plates in the manageme unstable ankle fractures: a comparative cohort study in 595 patients.	Gahr P, Matthis M, Schleese L, Fischer DC, Mittlmeier T.	Patient Saf Surg. 2024 Dec 3;18(1):33. doi: 10.1186/s13037-024-00419-7.	3
	Hindlimb biomechanics of Lagosuchus talampayensis (Archosauria, Dinosauriformes), with comments on skeletal morphology.	Otero A, Bishop PJ, Hutchinson JR.	J Anat. 2024 Dec 4. doi: 10.1111/joa.14183.	3
	Effects of ankle dorsiflexion training on anticipatory postural adjustments during gait initiation in patients with Parkinson's disease.	Nagai A, Marumoto K, Ohata K, Takasaki S, Moriyama H.	Gait Posture. 2025 Mar;117:109-114. doi: 10.1016/j.gaitpost.2024.12.015.	3
	Cadaveric Diagnostic Study of Subtle Syndesmotic Instability Using a 3-Dimensional Weight-Bearing CT Distance Mapping Algorith	de Cesar Netto C, Barbachan Mansur NS, Talaski G, et a.l	J Bone Joint Surg Am. 2025 Feb 19;107(4):397-407. doi: 10.2106/JBJS.24.001	3
	Comparing supine CT scanogram and standing long-leg radiograph for postoperative alignment in total knee arthroplasty: a prospective st	Mhaskar VA, Saggar R, Karan S, Maheshwari J.	Arch Orthop Trauma Surg. 2024 Dec 28;145(1):102. doi: 10.1007/s00402-024-0	3
	Comparing virtual reality and balance training effects on postural strategies during ball kicking in soccer players with chronic ankle instability.	Faghihi R, Khanmohammadi R.	Sci Rep. 2024 Dec 28;14(1):31448. doi: 10.1038/s41598-024-83071-6.	3
	Adding secondary cognitive tasks to drop vertical jumps alters the landing mechanics of athletes with anterior cruciate ligament reconstruc	Strong A, Markström JL.	J Biomech. 2025 Feb;180:112496. doi: 10.1016/j.jbiomech.2025.112496.	6
	Effects of lifestyle activity level on anticipatory locomotor adjustments for pedestrian circumvention: an exploratory st	Boulo J, Simon M, McFadyen BJ, Blanchette AK.	Exp Brain Res. 2025 Jan 18;243(2):48. doi: 10.1007/s00221-024-06980-y.	3
	The relationship between the supination resistance test, lower limb biomechanics and the effects of foot orthoses on foot and ankle biomechanics in individuals with posterior tibialis tendon dysfur during gait.	Moisan G, Dami A, Ghabdian T, Payen E, Isabelle PL, Farahp	Gait Posture. 2025 Mar;117:300-305. doi: 10.1016/j.gaitpost.2025.01.021.	3
	Orthotic bracing to treat equinus in children with spastic cerebral palsy: Recorded compliance and impact of wearing tir	Oestreich C, Böhm H, Hösl M, Döderlein L, Lewens D, Dussa	Gait Posture. 2025 May;118:75-84. doi: 10.1016/j.gaitpost.2025.01.034.	3
	Fully instrumented gait analysis in rare bone diseases - A scoping review of the literatu	Horn J, Leardini A, Benedetti MG, Hestnes TM, Mindler G, We	Gait Posture. 2025 May;118:168-177. doi: 10.1016/j.gaitpost.2025.02.001.	3
	Biomechanical Analysis of Cycle-Tempo Effects on Motor Control Among Jump Rope Elite	Zhou Q, Liu Y, Kang J, Wang X, Zhang K, Shan G.	Bioengineering (Basel). 2025 Feb 8;12(2):162. doi: 10.3390/bioengineering1202	3
	Gender Differences in Joint Biomechanics During Obstacle Crossing with Different Heigh	Wang C, Guo Y, Du W, Li Z, Chen W.	Bioengineering (Basel). 2025 Feb 16;12(2):189. doi: 10.3390/bioengineering120	3
	Comparison of Lower Limb Joint Reaction Forces in Patients with Cerebral Palsy and Typically Developing Individu	Dincel YM, Kidwai AN, Atmaca K, Sozener NA, Arslan YZ.	Medicina (Kaunas). 2025 Jan 31;61(2):246. doi: 10.3390/medicina61020246.	3
	Beliefs About and Use of Forefoot Lateral Wedging in Podiatric Medical Practice: A Survey of Podiatric Physicians in New Zealai	Jackson A, Sheerin K, Reid D, Carroll MR.	J Am Podiatr Med Assoc. 2025 Jan-Feb;115(1):22-022. doi: 10.7547/22-022.	3
	The effect of sex, skill level and a defender on cutting kinematics in soccer play	Chen K, Brown H, Guilmette S, Morelli M, Lamontagne A, Rob	Sports Biomech. 2025 Mar 25:1-18. doi: 10.1080/14763141.2025.2481496.	5
**Cochrane library**	Unanticipated Ankle Inversions Are Significantly Different From Anticipated Ankle Inversions During Drop Landings: overcoming Anticipation Bias	JR Dicus, JG Seegmiller	Journal of applied biomechanics, 2012, 28(2), 148155 |	2
	Open Reduction Syndesmosis Tightrope Versus Screw Fixation	NCT02199249	https://clinicaltrials.gov/show/NCT02199249, 2014	7
	The Influence of Circadian Variation on Etiological Markers of Ankle Injury	C Brogden, K Marrin, R Page, M Greig	Journal of sport rehabilitation, 2019, 28(5), 488493	2
	Effect of ankle taping on knee and ankle joint biomechanics in sporting tasks	KK Stoffel, RL Nicholls, AR Winata, AR Dempsey, JJ Boy	Medicine and science in sports and exercise, 2010, 42(11), 20892097	2
	Visualisation to enhance biomechanical tuning of ankle-foot orthoses (AFOs) in stroke: study protocol for a randomised controlled trial	B Carse, RJ Bowers, BC Meadows, PJ Rowe	Trials, 2011, 12, 25	2
	Patient-Preferred Prosthetic Ankle-Foot Alignment for Ramps and Level-Ground Walking	MK Shepherd, AM Simon, J Zisk, LJ Hargrove	IEEE transactions on neural systems and rehabilitation engineering, 2021, 29, 5529	2

	High-heel shoes wear affects postural control during gait initiation in healthy young female adults	A Delafontaine, P Fourcade, G Oustau, S Ditcharles, E Y	Computer methods in biomechanics and biomedical engineering, 2019, 22, S154-S15	3
	Uneven Treadmill Training for Rehabilitation of Lateral Ankle Sprains and Chronic Ankle Instability: Protocol for a Pragmatic Randomized Controlled Trial	E Russell Esposito, S Farrokhi, BR Shuman, PH Sessom	JMIR research protocols, 2022, 11(6), e38442	2
	Long latency postural responses are functionally modified by cognitive set	DJ Beckley, BR Bloem, MP Remler, RA Roos, JG Van Di	Electroencephalography and clinical neurophysiology, 1991, 81(5), 353-358	2
	Field hockey sport-specific postures during unanticipated sidestepping: implications for anterior cruciate ligament injury prevention	M Smith, G Weir, CJ Donnelly, J Alderso	Journal of sports sciences, 2020, 38(22), 2603-2610	2
	Effects of neurocentric training on the safety of unanticipated jump landings: a randomized controlled trial	DRKS00032171	https://trialsearch.who.int/Trial2.aspx?TrialID=DRKS00032171, 2023	7
	Changes in Postural Control After a Ball-Kicking Balance Exercise in Individuals With Chronic Ankle Instability	JS Conceição, FG Schaefer de Araújo, GM Santos, J Kei	Journal of athletic training, 2016, 51(6), 480-490	2
	Effects of strengthening, stretching and functional training on foot function in patients with diabetic neuropathy: results of a randomized controlled trial	CD Sartor, RH Hasue, LP Cacciari, MK Butugan, R Wata	BMC musculoskeletal disorders, 2014, 15(1)	2
	Combined effects of fatigue and decision making on female lower limb landing postures: central and peripheral contributions to ACL injury risk	BS Borotikar, R Newcomer, R Koppes, SG McLean	Clinical biomechanics (Bristol, Avon), 2008, 23(1), 81-92	2
	Impact of Powered Knee-Ankle Prosthesis Leg on Everyday Community Mobility and Social Interaction	NCT03204513	https://clinicaltrials.gov/show/NCT03204513, 2017	7
	Cochrane review of interventions for increasing ankle flexibility in neuromuscular disorders	KJ Rose, J Burns, D Wheeler, KN North	Journal of the peripheral nervous system, 2009, 14, 127-128	3
	The effect of functional training with audible-cues on cortico-motor excitability and motor unit behavior in athletes with chronic ankle instability	TCTR20230412001	https://trialsearch.who.int/Trial2.aspx?TrialID=TCTR20230412001, 2023	7
	Stance foot alignment and hand positioning alter star excursion balance test scores in those with chronic ankle instability: what are we really assessing?	M Cuğ	Physiotherapy theory and practice, 2017, 33(4), 316-322	2
	Gait patterns of children and adolescents with Charcot-Marie-Tooth disease	E Wojciechowski, A Sman, K Cornett, J Raymond, K Refs	Gait & posture, 2017, 56, 89-94	2
	Raised-heel stair-descent exercises as part of functional ambulation training post-stroke	ME Cochrane, CA Eksteen	Neurorehabilitation and neural repair, 2018, 32(4-5), 413-414	3
	Exploring the Impact of Child-Centered Play Therapy on Academic Achievement of At-Risk Kindergarten Students	PJ Blanco, RP Holliman, PL Ceballos, JL Farnam	International journal of play therapy, 2019, 28(3), 133-143	3
	Clinical use of a foot tapping test - A pilot study of agonist-antagonist coordination of the ankle joint in children with cerebral palsy	MB Speedtsberg, N Jacobsen, J Bencke, D Curtis	Gait & posture, 2017, 57, 42-43	3
	The mediating effect of coping styles and self-efficacy between perceived stress and satisfaction with QOL in Chinese adolescents with type 1 diabetes	J Yang, J Guo, Y Tang, L Huang, J Wiley, Z Zhou, R Whi	Journal of advanced nursing (john wiley & sons, inc.), 2019, 75(7), 1439-1449	3
	Comparison of lower-body 3D gait kinematics between Theia3D markerless and the CAST model marker-based systems in healthy adults and children	S D'souza, V Fohanno, S Schnell	Gait & posture, 2022, 97, S332-S333	3
	Effect of speed on mediolateral dynamic stability during stepping in older adults	T Caderby, J Begue, N Peyrot, G Dalleau	Computer methods in biomechanics and biomedical engineering, 2019, 22, S474-S47	3
	Intra-op biomechanical guidance improves articular fracture reduction, limiting post-traumatic OA risk	AM Kern, MC Willey, JL Marsh, DD Anderson	Osteoarthritis and cartilage, 2018, 26, S375	3
	Pain Induced during Both the Acquisition and Retention Phases of Locomotor Adaptation Does Not Interfere with Improvements in Motor Performance	J Bouffard, LJ Bouyer, JS Roy, C Mercier	Neural plasticity, 2016, 2016, 8539096	3
	Evaluation of a WeChat-based life review programme for cancer patients: a quasi-experimental study	X Zhang, H Xiao, Y Chen	Journal of advanced nursing (john wiley & sons, inc.), 2019, 75(7), 1563-1574	3
	A randomized crossover study of functional electrical stimulation during walking in spastic cerebral palsy: the FES on participation (FESPa) trial	I Moll, RGJ Marcellis, MLP Coenen, SM Fleuren, PJB Wil	BMC pediatrics, 2022, 22(1)	2
	Research protocol to evaluate the effectiveness of shockwave therapy, photobiomodulation and physical therapy in the management of non-insertional Achilles tendinopathy in runners: a randomised control tri	AS Tenforde, KEL Vogel, J Tam, KG Silbernagel	BMJ open sport & exercise medicine, 2022, 8(3), e001397	3
	Primed to perform: comparing different pre-performance routine interventions to improve accuracy in closed, self-paced motor tasks	C Mesagno, J Beckmann, VV Wergin, P Gröpel	Psychology of sport and exercise, 2019, 43, 73-81	3
	Do athletes' responses to coach autonomy support and control depend on the situation and athletes' personal motivation?	J Delrue, B Soenens, S Morbée, M Vansteenkiste, L Hae	Psychology of sport and exercise, 2019, 43, 321-332	3
	The Effect of Varying Prosthetic Foot Stiffness on Perceived Stiffness and Gait Biomechanics During Walking	J Czerniecki, E Halsne, D Morgenroth	Archives of physical medicine and rehabilitation, 2019, 100(10), e128-e129	3
	Balance and recovery on coronally-uneven and unpredictable terrain	KH Yeates, AD Segal, RR Neptune, GK Klute	Journal of biomechanics, 2016, 49(13), 2734-2740	2
	Dynamic biomechanical model of the hand and arm in pistol grip power handtool usage	JH Lin, RG Radwin, TG Richard	Ergonomics, 2001, 44(3), 295-312	2
	Effectiveness of EMS Versus TENS During Gait Training in Post Stroke Patients to Improve Gait and Quality of Life	NCT05738811	https://clinicaltrials.gov/show/NCT05738811, 2023	3
	Are measures of hindfoot alignment sensitive to pathology and correction?	AM Kern, C Anthony, JE Goetz, R Schumer, A Kruse, JE	Journal of orthopaedic research, 2017, 35	3
	A Quasi-Trial Investigation of an In-Service Training to Improve Social Workers' Professional Competence in China	H Zhang, Y Wang, Z Liu, EW Chui	Research on social work practice, 2019, 29(5), 506-518	3
	The Effectiveness of the Young–Old Link and Growth Intergenerational Program in Reducing Age Stereotypes	Q Sun, VW Lou, A Dai, C To, SY Wong	Research on social work practice, 2019, 29(5), 519-528	3
	Effects of strengthening, stretching and functional training on foot function in patients with diabetic neuropathy: results of a randomized controlled trial	CD Sartor, RH Hasue, LP Cacciari, MK Butugan, R Wata	BMC musculoskeletal disorders, 2014, 15, 137	2
	Spatio-temporal separation of roll and pitch balance-correcting commands in humans	C Grüneberg, J Duysens, F Honegger, JH Allum	Journal of neurophysiology, 2005, 94(5), 3143-3158	2
	Upper cervical spine trauma: WFNS spine committee recommendations	OL Alves, L Pereira, S-H Kim, A Grin, N Shimokawa, N K	Neurospine, 2020, 17(4), 723-736	3
	Cerebellar control of postural scaling and central set in stance	FB Horak, HC Diener	Journal of neurophysiology, 1994, 72(2), 479-493	2
	A randomized crossover study of functional electrical stimulation during walking in spastic cerebral palsy: the FES on participation (FESPa) trial	I Moll, RGJ Marcellis, MLP Coenen, SM Fleuren, PJB Wil	BMC pediatrics, 2022, 22(1), 37	2
	Effects of education and support groups organized by IBCLCs in early postpartum on breastfeeding	Y Lee, G Chang, H Chang	Midwifery, 2019, 75, 5-11	3
	Role-play versus lecture methods in community health volunteers	F Vizeshfar, M Zare, Z Keshtkaran	Nurse education today, 2019, 79, 175-179	3
	Effect of case study versus video simulation on nursing students' satisfaction, self-confidence, and knowledge: a quasi-experimental study	EK Herron, K Powers, L Mullen, B Burkhart	Nurse education today, 2019, 79, 129-134	3
	Transfer of improved movement technique after receiving verbal external focus and video instruction	A Benjaminse, W Welling, B Otten, A Gokeler	Knee surgery, sports traumatology, arthroscopy, 2018, 26(3), 955-962	2
	Biomechanical Effects of an Injury Prevention Program in Preadolescent Female Soccer Athletes	K Horner, B Summerhays, K Fiala, KM Schweser	Journal of foot and ankle surgery, 2023, 62(3), 487-491	2
	Targeted ballet program for people with multiple sclerosis	ISRCTN67916624	https://trialsearch.who.int/Trial2.aspx?TrialID=ISRCTN67916624, 2017	3
	Similarity of joint kinematics and muscle demands between elliptical training and walking: implications for practice	JM Burnfield, Y Shu, T Buster, A Taylor	Physical therapy, 2010, 90(2), 289-305	2
	The Effect of Bikram Yoga on Arterial Stiffness and Endothelial Function in Middle-Aged Adults	NCT02488148	https://clinicaltrials.gov/show/NCT02488148, 2015	7
	Pulmonary Rehabilitation Program and PROactive Tool	NCT02437994	https://clinicaltrials.gov/show/NCT02437994, 2015	7
	Online Simulation-Based Mastery Learning with Deliberate Practice: developing Interprofessional Communication Skill	VJ-H Yeh, G Sherwood, CF Durham, S Kardong-Edgren,	Clinical simulation in nursing, 2019, 32, 27-38	3
	Alfredson versus Silbernagel exercise therapy in chronic midportion Achilles tendinopathy: study protocol for a randomized controlled trial	B Habets, REH van Cingel, FJG Backx, BMA Huisstede	BMC musculoskeletal disorders, 2017, 18(1), 296	3
	Understanding the impact of footwear on young children's gait	ACTRN12617000999336	http://www.who.int/trialsearch/Trial2.aspx?TrialID=ACTRN12617000999336, 2017	3
	Patellar tendon pain: risk identification in athletes	RBR-4frygy	https://trialsearch.who.int/Trial2.aspx?TrialID=RBR-4frygy, 2017 | added to CENTRA	3
	Comparison of Functional Bracing vs Rigid Immobilization After Modified Percutaneous Achilles Tendon Repair	NCT04692883	https://clinicaltrials.gov/show/NCT04692883, 2021	7
	Quantitative Assessment of Training Effects Using EKSOGT Exoskeleton in Quantitative Assessment of Training Effects Using EKSOGT Exoskeleton in Parkinson Disease Patients	NCT04778852	https://clinicaltrials.gov/show/NCT04778852, 2021	7
	A Comprehensive Analysis for Identification of Risk Factors for Running-Related Injuries	NCT04642248	https://clinicaltrials.gov/show/NCT04642248, 2020	7
	Early Intervention Program for Preterm Infants and Their Parents: establishing the Impact at 18 Months Corrected Age	NCT02835612	https://clinicaltrials.gov/show/NCT02835612, 2016	7
	Effect of Milk Fat Globule Membrane (MFGM) on Gut Barrier Protection in Runners	NCT03176212	https://clinicaltrials.gov/show/NCT03176212, 2017	7
	Treatment of Impending Ulcers Associated With Hammer, Mallet and Claw Toe Deformities in the Diabetic Patient Setting	NCT04154046	https://clinicaltrials.gov/show/NCT04154046, 2019	7
	Maraviroc to Augment Rehabilitation Outcomes After Stroke	NCT03172026	https://clinicaltrials.gov/show/NCT03172026, 2017	7
	Robotic Gait Training in Spinal Cord Injury	NCT02749357	https://clinicaltrials.gov/show/NCT02749357, 2016	7
	Effects of a care program for risk factors for falls in a prevention program for elderly people who fall: a randomized clinical trial	RBR-3t85fd	https://trialsearch.who.int/Trial2.aspx?TrialID=RBR-3t85fd, 2020	7
	SECONDARY COGNITIVE TASKS ALTER DROP VERTICAL JUMP LANDING MECHANICS AND REDUCE JUMP HEIGHT IN INDIVIDUALS WITH ANTERIOR CRUCIATE LIGAMENT INJURY	Strong A, Markstrom J	BMJ open sport and exercise medicine, 2024, 10, A5	6
	Effects of Combining Transcranial Direct Current Stimulation With Balance Training on Anticipatory Postural Adjustments in Persons With Chronic Ankle Instability	Beyraghi Z, Khanmohammadi R, Hadian MR.	Sports Health. 2024 May 8:19417381241247746.	2
	Kinesio Taping Effects on Balance and Ankle Proprioception	NCT06832111	https://clinicaltrials.gov/ct2/show/NCT06832111	3
	Effects of ankle dorsiflexion training on anticipatory postural adjustments during gait initiation in patients with Parkinson's disease	A Nagai, K Marumoto, K Ohata, S Takasaki, H Moriyama	Gait & posture, **2025**, 117, 109-114	2
	Comparing virtual reality and balance training effects on postural strategies during ball kicking in soccer players with chronic ankle instability.	Faghihi R, Khanmohammadi R.	Sci Rep. 2024 Dec 28;14(1):31448. doi: 10.1038/s41598-024-83071-6.	2
	The Effects of Stroboscopic Balance Training in the Individuals with Chronic Ankle Instability	TCTR20240923003	https://trialsearch.who.int/Trial2.aspx?TrialID=TCTR20240923003, **2024**	3
	Comparison of the effects of exergame and balance training on dynamic postural stability	IRCT20230124057209N2	https://trialsearch.who.int/Trial2.aspx?TrialID=IRCT20230124057209N2, **20**	3
	NEUROBALANCE Training to Improve Postural Control in Individuals with Traumatic Brain Injury	NCT06584591	https://clinicaltrials.gov/ct2/show/NCT06584591, **2024**	3
	Increased Visual Attentional Demands Alter Lower Extremity Sidestep Cutting Kinematics in Male Basketball Players	KTH Rikken, T Panneman, F Vercauteren, A Gokeler, A E	International journal of sports physical therapy, **2024**, 19(11), 1304-1313	1
	Effects of resistance training with blood flow restriction on pain reduction, physical function improvement, and quality of life in patients with fibromyalgia	RBR-3mj5vhs	https://trialsearch.who.int/Trial2.aspx?TrialID=RBR-3mj5vhs, 2025	3
**science direct**	Forward dynamics computational modelling of a cyclist fall with the inclusion of protective response using deep learning-based human pose estimation			3
	Muscle contributions to reduced ankle joint contact force during drop vertical jumps in patients with chronic ankle instability			3
	Tibiotalocalcaneal arthrodesis with an intramedullary nail: The functional and clinical outcome of a challenging patient group and its comparison to a below knee amputation			3
	Understanding the effects of a sudden directional shift in somatosensory feedback and increasing task complexity on postural adaptation in individuals with and without chronic ankle instability			3
	Early experience with a 3-D printed porous surface, fixed-bearing, total ankle arthroplasty: A minimum of 2-year follow-up			3
	Effect of Sensor Location for Modifying Center of Pressure During Gait using Haptic Feedback in People with Chronic Ankle Instability			3
	Symmetric unipedal balance in quiet stance and dynamic tasks in older individuals			3
	Falling Decreased Anterior Cruciate Ligament Loading Variables during Single-leg Landings after Mid-flight External Trunk Perturbation			3
	Cortical activity and spatiotemporal parameters during gait termination and walking: A preliminary study			3
	A pelvis-oriented margin of stability is robust against deviations in walking direction			3
	Development of an experimental method for well-controlled blast induced traumatic limb fracture in rats			3
	PTEN knockout using retrogradely transported AAVs transiently restores locomotor abilities in both acute and chronic spinal cord injury			3
	Diabetes mellitus as a risk factor for postoperative complications following arthroscopic rotator cuff repair			3
	Food nutrition and toxicology targeting on specific organs in the era of single-cell sequencing			3
	Effect of age and speed on the step-to-step transition strategies in children			3
	Differences in lower extremity joint stiffness during drop jump between healthy males and females			3
	Short partial doffs of release/relock sockets may effectively stabilize limb fluid volume in prosthesis users with transtibial amputation			3
	Axial muscle activation provides stabilization against perturbations while running			3
	Outcome analysis of ilizarov and monorail fixators in the treatment of nonunion of long bones: A systematic review and proportion meta-analysis			3
	Effects of gender and fatigue on strength and activity of gluteus medius muscle during a controlled cutting maneuver in preadolescent athletes			3
	An Artificial Intelligence model for smart post-stroke assessment using wearable sensors			3
	Ankle fusion outcomes utilizing anterior ankle plating techniques: A systematic review			3
	Lower body kinematics estimation during walking using an accelerometer			3
	Susceptibility to walking balance perturbations in young adults is largely unaffected by anticipation			3
	The effect of neuromuscular and vestibular-ocular reflex training program on balance, isokinetic muscle strength and proprioception in people with chronic ankle instability			3
	Reactive postural responses predict risk for acute musculoskeletal injury in collegiate athletesJournal of Science and Medicine in Sport11 January 2023 Amanda MorrisNora F. FinoPeter C. Fino			3
	Reliability of the running vertical jump test in female team sport athletes			3
	Indirect contact matters: Mid-flight external trunk perturbation increased unilateral anterior cruciate ligament loading variables during jump-landings			3
	Are proximal and distal neuromuscular parameters able to predict hip and knee frontal plane kinematics during single-leg landing?			3
	The Incidence of Complications Following Scarf Osteotomy for the Treatment of Hallux Valgus: A Systematic Review With Meta-Analysis			3

Radiographic Evaluation of Isolated Continuous Compression Staples for Akin Osteotomy Fixation	2
Effects of movement direction and limb dominance on ankle muscular force in sidestep cutting	3
A two-stage disto-proximal braking modality to interrupt gait initiation in healthy adults	3
Foot health status in pregnant women	3
Is there association between cutting and jump-landing movement quality in semi-professional football players? Implications for ACL injury risk screening	3
Orthopaedic Innovation and the Balance With Conflicts of Interest	3
Utility of an obstacle-crossing test to classify future fallers and non-fallers at hospital discharge after stroke: A pilot study	3
A structured accelerated versus control rehabilitation pathway after anterior cruciate ligament reconstruction using autologous hamstrings demonstrates earlier improvement in physical outcomes without increasing graft laxity: A randomized controlled trial	3
Effect of foot orthoses vs sham insoles on first metatarsophalangeal joint osteoarthritis symptoms: a randomized controlled trial	3
Clinicians' experience of the diagnosis and management of patellofemoral pain: A qualitative exploration	3
Females exhibit lower limb biomechanics associated with an increased risk of ACL injury during a handball-specific side stepping	3
Unimpaired performance during cognitive and visual manipulations in persons with anterior cruciate ligament reconstruction compared to healthy adults	3
Minimum 5-Year Outcomes of a Multicenter, Prospective, Randomized Control Trial Assessing Clinical and Radiological Outcomes of Patient-Specific Instrumentation in Total Knee Arthroplasty	3
Mass customization and testing of braces using additive manufacturing	3
Biomechanical evaluation of a novel 3D printing tibiotalocalcaneus nail with trilobular cross-sectional design and self-compression effect	3
Differential activation of the plantar flexor muscles in balance control across different feet orientations on the ground	3
Proprioceptive postural control strategies differ among non-injured athletes	3
A Comparison of Trans-Articular Screw Versus Dorsal Bridge Plate Versus Compression Locking Plate Fixation in B2 Lis Franc Injuries: A 5-Year Experience in a Level 1 Trauma Center, in the United Kingdom	3
Effects of foot progression angle on knee mechanics during an anticipated cutting task: A statistical parametric mapping approach	3
Operative Ankle Fractures in Complicated Diabetes: Outcomes of Prolonged Non-Weightbearing	3
Are Transmetatarsal Amputations a Durable Limb Salvage Option? A Single-Institution Descriptive Analysis	3
Acquisition of mechanical energy directly contributing to sideward propulsion in sidestep cutting manoeuvre	3
Acute effects of a neuromuscular warm-up on potential re-injury risk factors associated with unanticipated jump landings after anterior cruciate ligament reconstruction: A crossover trial	4
Arthrodesis in Acute and Chronic Lisfranc's Patients: A Retrospective Cohort Study	3
The influence of age and fall history on single transition step kinematics	3
Changes in landing mechanics using augmented feedback: 4-Week training and retention study	3
Neurocognitive function influences dynamic postural stability strategies in healthy collegiate athletes	3
When puberty strikes: Longitudinal changes in cutting kinematics in 172 high-school female athletes	3
Sex differences in muscle activation patterns associated with anterior cruciate ligament injury during landing and cutting tasks: A systematic review	3
Return-to-sport following anterior cruciate ligament reconstruction in team sport athletes. Part I: From initial injury to return-to-competition	3
The Clinical Efficacy of Suture-Button Fixation and Trans-Syndesmotic Screw Fixation in the Treatment of Ankle Fracture Combined With Distal Tibiofibular Syndesmosis Injury: A Retrospective Study	3
An optimized and chaotic intelligent system for a 3DOF rehabilitation robot for lower limbs based on neural network and genetic algorithm	3
Muscle force contributions to ankle joint contact forces during an unanticipated cutting task in people with chronic ankle instability	5
1-Year Results From the RANGER II SFA Randomized Trial of the Ranger Drug-Coated BalloonJACC: Cardiovascular Interventions17 May 2021 Ravish SacharYoshimitsu SogaThomas Zeller	3
Automated analysis of medial gastrocnemius muscle-tendon junction displacements in heathy young adults during isolated contractions and walking using deep neural networks	3
Reduction of Risk Factors for ACL Re-injuries using an Innovative Biofeedback Approach: Rationale and Design	3
The PITCH study: pitcher injuries during the first 30 days of the coronavirus disease 2019 halted Major League Baseball season	3
Lower extremity muscle contributions to ACL loading during a stop-jump task	5
Fewer reoperations after posterolateral plate positioning compared with lateral plate positioning in ankle fractures—a retrospective study on 453 AO/OTA 44-B injuriesInjury	3
A longitudinal investigation of landing biomechanics following anterior cruciate ligament reconstruction	3
Occupational safety and health in marine aquaculture in Atlantic Canada: What can be learned from an analysis of provincial occupational injury compensation claims data?	3
Infected Tibial Nonunion: Assessment of compression distraction Ilizarov technique without debridement	3
An instrument for methodological quality assessment of single-subject finite element analysis used in computational orthopaedics	3
Daily acute intermittent hypoxia combined with walking practice enhances walking performance but not intralimb motor coordination in persons with chronic incomplete spinal cord injury	3
The use of circular frame external fixation in the treatment of ankle/hindfoot Charcot Neuroarthropathy	3
Which jump-landing task best represents lower extremity and trunk kinematics of unanticipated cutting maneuver?	2
Short and long versions of a 12-week netball specific neuromuscular warm-up improves landing technique in youth netballers	3
Outcomes of Drug-Coated Balloon Angioplasty for Isolated Chronic Occlusion of the Popliteal Artery: A Retrospective Single-Institution Study	3
Hemodialysis Centers Guide 2020	3
Comparative Tribology: Articulation-induced Rehydration of Cartilage Across Species	3
Trunk, pelvis and lower limb coordination between anticipated and unanticipated sidestep cutting in females	2
Non-specific chronic low back pain elicits kinematic and neuromuscular changes in walking and gait termination	3
Analysis of invoked slips while wearing flip-flops in wet and dry conditions: Does alternative footwear alter slip kinematics?	3
Notions of "optimal" posture are loaded with meaning. Perceptions of sitting posture among asymptomatic members of the community	3
Comparing walking biomechanics of older females in maximal, minimal, and traditional shoes	3
Sex-specific landing biomechanics and energy absorption during unanticipated single-leg drop-jumps in adolescents: implications for knee injury mechanics	2
Time-frequency analysis of muscle activation patterns in people with chronic ankle instability during Landing and cutting tasks	2
The coordination patterns of the foot segments in relation to lateral ankle sprain injury mechanism during unanticipated changes of direction	2
Reducing knee pain and loading with a gait retraining program for individuals with knee osteoarthritis: Protocol for a randomized feasibility trial	3
Prescribing joint co-ordinates during model preparation in OpenSim improves lower limb unplanned sidestepping kinematics	5
Endocan: A novel biomarker for risk stratification, prognosis and therapeutic monitoring in human cardiovascular and renal diseases	3
Increased flexor hallucis longus tension decreases ankle dorsiflexion	3
The effect of silicone ankle sleeves and lace-up ankle braces on neuromuscular control, joint torque, and cutting agility	3
Development and Validation of Wearable Inertial Sensor System for Postural Sway Analysis	3
Divergence analysis of failed and successful unanticipated single-leg landings reveals the importance of the flight phase and upper body biomechanics	5
An exploration of the experiences of people living with painful ankle osteoarthritis and the non-surgical management of this condition	3
Biomechanics during cross-body lunging in individuals with and without painful cam and/or pincer morphology	3
Can kinematic and kinetic differences between planned and unplanned volleyball block jump-landings be associated with injury risk factors?	2
Individuals with unilateral transtibial amputation exhibit reduced accuracy and precision during a targeted stepping task	3
Differences in anterior cruciate ligament injury risk factors between female dancers and female soccer players during single- and double-leg landing	3
Cortical suture button fixation vs. bicortical screw fixation in the Latarjet procedure: a biomechanical comparison	3
Iatrogenic Articular Cartilage Injury in Arthroscopic Hip and Knee Videos and the Potential for Cartilage Cell Death When Simulated in a Bovine Model	3
Whole-body dynamic stability in side cutting: Implications for markers of lower limb injury risk and change of direction performance	5
Assessing in vivo articular cartilage mechanosensitivity as outcome of high tibial osteotomy in patients with medial compartment osteoarthritis: Experimental protocol	3
Outcome of Ray Resection as Definitive Treatment in Forefoot Infection or Ischemia: A Cohort Study	3
Athletic groin pain patients and healthy athletes demonstrate consistency in their movement strategy selection when performing multiple repetitions of a change of direction test	3
The timing of locomotor propulsion in healthy adults walking at multiple speeds	3
Altered gait mechanics are associated with severity of chondropathy after hip arthroscopy for femoroacetabular impingement syndrome	3
Consent in foot and ankle surgery	3
Talectomy as Part of Chronic Foot and Ankle Deformity Correction Procedure: A Retrospective Study	3
Anterior fall-recovery training applied to individuals with chronic stroke	3
On-field player workload exposure and knee injury risk monitoring via deep learning	3
Nurses and Orals Abstracts	3
Aging effects of motor prediction on protective balance and startle responses to sudden drop perturbations	3
Reliability of measures of dynamic stability for the assessment of balance recovery after a forward loss of balance	2
Sex differences in lower extremity coordinative variability during running	2
Reliability of measures of dynamic stability for the assessment of balance recovery after a forward loss of balance	2
Sex differences in lower extremity coordinative variability during running	2
Anticipating ankle inversion perturbations during a single-leg drop landing alters ankle joint and impact kinetics	2
The effect of the NetballSmart Dynamic Warm-up on physical performance in youth netball players	3
Gait termination on declined compared to level surface; contribution of terminating and trailing limb work in arresting centre of mass velocity	3
Risk Factors Associated with 30-Day Mortality After Open Reduction and Internal Fixation of Vertebral Fractures	3
Surgical Treatment of Developmental Spondylolisthesis: Contemporary Series With a Two-Surgeon Team	3
Right in Comparison to Left Cerebral Hemisphere Damage by Stroke Induces Poorer Muscular Responses to Stance Perturbation Regardless of Visual Information	3
Suture button versus syndesmosis screw constructs for acute ankle diastasis injuries: A meta-analysis and systematic review of randomised controlled trials	3
Knee mechanics during a change of direction movement in division I athletes following full return to sport from anterior cruciate ligament reconstruction	3
Multidisciplinary sarcoma care	3
Understanding cutting maneuvers – The mechanical consequence of preparatory strategies and foot strike pattern	3
The effects of cognitive load and optical flow on antagonist leg muscle coactivation during walking for young and older adults	3
Aging and Chronic Disease	3
Application of a Ni-Ti arched shape-memory connector in unstable lateral malleolus fractures: A retrospective study	3

Lower-limb muscle function during sidestep cutting	5
Central adiposity and mechanical, perceptual and physiological loading during long duration, repetitive lifting	3
Biomechanical but not timed performance asymmetries persist between limbs 9 months after ACL reconstruction during planned and unplanned change of direction	2
Hip-abductor fatigue influences sagittal plane ankle kinematics and shank muscle activity during a single-leg forward jump	3
Young and older adults adapt automatic postural responses equivalently to repetitive perturbations but are unable to use predictive cueing to optimize recovery of balance stability	2
Aging effects on the Achilles tendon moment arm during walking	3
Kinematic algorithm to determine the energy cost of running with changes of direction	3
Comparing the effects of mechanical perturbation training with a compliant surface and manual perturbation training on joints kinematics after ACL-rupture	3
Through-knee amputation is a feasible alternative to above-knee amputation	3
Light touch leads to increased stability in quiet and perturbed balance: Equivalent effects between post-stroke and healthy older individuals	3
Differences in neuromuscular activity of ankle stabilizing muscles during postural disturbances: A gender-specific analysis	3
Increased movement variability in one-leg hops about 20 years after treatment of anterior cruciate ligament injury	3
Biomechanical and physiological age differences in a simulated forward fall on outstretched hands in women	3
Long-Term Results of Hemiarthroplasty Compared With Arthrodesis for Osteoarthritis of the First Metatarsophalangeal Joint	3
Feasibility of a bone and soft tissue chimeric anterolateral thigh free flap? Anatomic study and report of two cases for oral cavity reconstruction	3
Effect of Taijiquan practice versus wellness education on knee proprioception in patients with knee osteoarthritis: a randomized controlled trial	3
Biomechanical adaptations during running differ based on type of exercise and fitness level	3
Prediction of ground reaction forces for Parkinson's disease patients using a kinect-driven musculoskeletal gait analysis model	3
Dual-task and anticipation impact lower limb biomechanics during a single-leg cut with body borne load	4
Strategy quantification using body worn inertial sensors in a reactive agility task	3
Right cerebral hemisphere specialization for quiet and perturbed body balance control: Evidence from unilateral stroke	3
Innovations and pitfalls in the use of wearable devices in the prevention and rehabilitation of running related injuries	3
Impact of ankle foot orthosis stiffness on Achilles tendon and gastrocnemius function during unimpaired gait	3
Pilates Reformer exercises for fall risk reduction in older adults: A randomized controlled trial	3
Kinematic differences during a jump cut maneuver between individuals with and without a concussion history	3
Hybrid-state driven autonomous control for planar bipedal locomotion over randomly sloped non-uniform stairs	3
Effect of limb dominance and sex on neuromuscular activation patterns in athletes under 12 performing unanticipated side-cuts	5
Automatic postural responses are generated according to feet orientation and perturbation magnitude	3
Effects of amplitude and predictability of perturbations to the arm on anticipatory and reactionary muscle responses to maintain balance	2
Identification and risk estimation of movement strategies during cutting maneuvers	3
Shoe cushioning reduces impact and muscle activation during landings from unexpected, but not self-initiated, drops	3
The use of non-slip socks to prevent falls among hospitalized older adults: A literature review	3
The independent effects of speed and propulsive force on joint power generation in walking	3
The Impact of Mechanical and Restricted Kinematic Alignment on Knee Anatomy in Total Knee Arthroplasty	3
Biomechanical demand analysis of older passengers in a standing position during bus transport	3
Preparatory co-activation of the ankle muscles may prevent ankle inversion injuries	2
Non-MTC gait cycles: An adaptive toe trajectory control strategy in older adults	3
The effect of performance demands on lower extremity biomechanics during landing and cutting tasks	3
Higher order balance control: Distinct effects between cognitive task and manual steadiness constraint on automatic postural responses	3
Replacement of daily load attenuates but does not prevent changes to the musculoskeletal system during bed rest	3
An examination of the startle response during upper limb stretch perturbations	3
Distinct cut task strategy in Australian football players with a history of groin pain	2
Biomechanical analysis of gait termination in 11–17year old youth at preferred and fast walking speeds	3
External fixation versus open reduction and internal fixation for tibial pilon fractures: A meta-analysis based on observational studies	3
Joint dynamics of rear- and fore-foot unplanned sidestepping	2
Hybrid-state driven autonomous control for planar bipedal locomotion	3
Braking characteristics during cutting and pivoting in female soccer players	3
Preliminary development of a complex intervention for osteopathic management of dysfunctional breathing	3
Effects of differences in visual acuity on gait time and trunk acceleration when older women negotiate stairs	3
Safety, Tolerability, Pharmacokinetic and Pharmacodynamic Properties of SBI-087, a CD20-Directed B-cell Depleting Agent: Phase 1 Dose Escalating Studies in Patients With Either Mild Rheumatoid Arthritis or	3
A sex comparison of reactive knee stiffness regulation strategies under cognitive loads	3
The effects of fatigue and anticipation on the mechanics of the knee during cutting in female athletes	4
Reliability and sensitivity of a novel dynamic balance test for alpine skiers	3
Effects of Tai Ji Quan training on gait kinematics in older Chinese women with knee osteoarthritis: A randomized controlled trial	3
Kinematic TKA using navigation: Surgical technique and initial results	3
Muscle activation timing and balance response in chronic lower back pain patients with associated radiculopathy	3
Phase resetting behavior in human gait is influenced by treadmill walking speed	3
Technique determinants of knee abduction moments during pivoting in female soccer players	3
Evaluation of ergonomic approach and musculoskeletal disorders in two different organizations in a truck assembly plant	3
Sensory reweighting is altered in adolescent patients with scoliosis: Evidence from a neuromechanical model	3
Ranking risk exposures for situational surveillance of falls with sensors	3
Technique determinants of knee joint loads during cutting in female soccer players	3
Lower limb kinematics of male and female soccer players during a self-selected cutting maneuver: Effects of prolonged activity	3
Contribution of lower limb eccentric work and different step responses to balance recovery among older adults	2
Acceleration and Orientation Jumping Performance Differences Among Elite Professional Male Handball Players With or Without Previous ACL Reconstruction: An Inertial Sensor Unit-Based Study	3
Lagged Syndesmotic Fixation: Our Clinical Experience	3
24-Month Data from the BRAVISSIMO: A Large-Scale Prospective Registry on Iliac Stenting for TASC A & B and TASC C & D Lesions	3
Low back pain affects trunk as well as lower limb movements during walking and running	3
Age-related changes in trunk neuromuscular activation patterns during a controlled functional transfer task include amplitude and temporal synergies	3
The effect of different methods of stability assessment on fixation rate and complications in supination external rotation (SER) 2/4 ankle fractures	3
Soldier-relevant loads impact lower limb biomechanics during anticipated and unanticipated single-leg cutting movements	2
Correction of deformity—The implant is never enough!	3
Talar neck fracture—A rare but important complication following subtalar arthroereisis	3
Effect of turf on the cutting movement of female football players	3
Dance floor force reduction influences ankle loads in dancers during drop landings	3
Improved Radiographic Outcomes With Patient-Specific Total Knee Arthroplasty	3
Reduced hamstring strength increases anterior cruciate ligament loading during anticipated sidestep cutting	3
Slipping during side-step cutting: Anticipatory effects and familiarization	3
Effect of lace-up ankle braces on electromyography measures during walking in adults with chronic ankle instability	3
Inter-session reliability and sex-related differences in hamstrings total reaction time, pre-motor time and motor time during eccentric isokinetic contractions in recreational athlete	3
Lower limb joint motion during a cross cutting movement differs in individuals with and without chronic ankle instability	2
Expecting ankle tilts and wearing an ankle brace influence joint control in an imitated ankle sprain mechanism during walking	2
Step length after discrete perturbation predicts accidental falls and fall-related injury in elderly people with a range of peripheral neuropathy	3
Dynamic stability of a human standing on a balance board	3
Automated detection of gait initiation and termination using wearable sensors	3
Stepping in Persons Poststroke: Comparison of Voluntary and Perturbation-Induced Responses	3
Anticipatory effects on anterior cruciate ligament loading during sidestep cutting	2
Evaluating runners with and without anterior knee pain using the time to contact the ankle joint complexes' range of motion boundary	3
Spatio-temporal parameters and lower-limb kinematics of turning gait in typically developing children	3
A comparison of lateral ankle ligament suture anchor strength	3
The Effect of a Knee-ankle Restraint on ACL Injury Risk Reduction during Jump-landing	3
Whole-Body Vibration During Passive Standing in Individuals With Spinal Cord Injury: Effects of Plate Choice, Frequency, Amplitude, and Subject's Posture on Vibration Propagation	3
COMAP: A new computational interpretation of human movement planning level based on coordinated minimum angle jerk policies and six universal movement elements	3
Isolated calcaneofibular ligament injuries treated with osteopathic manipulative treatment: A case series	3
Evaluation of gait and slip parameters for adults with intellectual disability	3
Kinematic and kinetic analysis of planned and unplanned gait termination in children	8
Biomechanics of lower limb haemophilic arthropathy	3
Nutritional status and gastrointestinal symptoms in systemic sclerosis patients	3
Sensorimotor and neuropsychological correlates of force perturbations that induce stepping in older adults	3
113th Annual Meeting of the American Associaton of Colleges of Pharmacy, Kissimmee, FL, July 14-18, 2012	3
Knee moments during run-to-cut maneuvers are associated with lateral trunk positioning	3

Musculoskeletal changes following non-invasive knee injury using a novel mouse model of post-traumatic osteoarthritis	3
Principal component based analysis of biomechanical inter-trial variability in individuals with chronic ankle instability	3
Optimizing whole-body kinematics to minimize valgus knee loading during sidestepping: Implications for ACL injury risk	8
Relationship between cartilage and subchondral bone lesions in repetitive impact trauma-induced equine osteoarthritis	3
Gait termination strategies differ between those with and without ankle instability	2
Ice hockey skate boot mechanics: Direct torque and contact pressure measures	3
A qualitative study on overuse injuries: The beliefs of athletes and coaches	3
Landing technique affects knee loading and position during athletic tasks	2
Ageing and limb dominance effects on foot-ground clearance during treadmill and overground walking	3
Knee and hip sagittal and transverse plane changes after two fatigue protocols	3
Prevention of Diabetic Neuropathy by Regulatable Expression of HSV-Mediated Erythropoietin	3
ACL Injury Prevention in the Athlete	3
Simulation of human movement: applications using OpenSim	3
The effect of varying footwear configurations on the peroneus longus muscle function following inversion	3
Reamer-irrigator-aspirator bone graft and bi Masquelet technique for segmental bone defect nonunions: a review of 25 cases	3
Modulation of Integrin Activation by an Entropic Spring in the β-Knee	3
Balance recovery after an evoked forward fall in unilateral transtibial amputees	3
Gait retraining to reduce the knee adduction moment through real-time visual feedback of dynamic knee alignment	3
Gait adaptations in response to perturbations in adults with Down syndrome	3
Efficacy of Intra-Articular Botulinum Toxin Type A in Painful Knee Osteoarthritis: A Pilot Study	3
Lower limb muscle pre-motor time measures during a choice reaction task associate with knee abduction loads during dynamic single leg landings	4
The Ilizarov Method of External Fixation: Current Intraoperative Concepts	3
Prospective, Randomized Single-center Trial to Compare Cryoplasty versus Conventional Angioplasty in the Popliteal Artery: Midterm Results of the COLD Study	3
Anatomic Double-Bundle Anterior Cruciate Ligament Reconstruction: Kinematics and Knee Flexion Angle–Graft Tension Relation	3
Flexible intramedullary nailing in paediatric femoral shaft fractures	3
A comparison of subtalar joint motion during anticipated medial cutting turns and level walking using a multi-segment foot model	3
Kinematics and kinetics of unanticipated misstep conditions: Femoral fracture implications in the elderly	3
The influence of foot position on body dynamics	3
Joint-specific power production and fatigue during maximal cycling	3
AMA-MOSAICI: An automatic module assigning hierarchical structure to control human motion based on movement decomposition	3
The effect of isolated valgus moments on ACL strain during single-leg landing: A simulation study	3
Quantitative evaluation of balance in patients with spinocerebellar ataxia type 1: A case control study	3
Gender differences exist in neuromuscular control patterns during the pre-contact and early stance phase of an unanticipated side-cut and cross-cut maneuver in 15–18 years old adolescent soccer players	3
Selective Dorsal Rhizotomy in Hong Kong: Multidimensional Outcome Measures	3
Fatigue-related changes in stance leg mechanics during sidestep cutting maneuvers	3
Effect of the HamSprint Drills training programme on lower limb neuromuscular control in Australian football players	3
Combined effects of fatigue and decision making on female lower limb landing postures: Central and peripheral contributions to ACL injury risk	2
Industrial Medicine and Acute Musculoskeletal Rehabilitation. 1. Diagnostic Testing in Industrial and Acute Musculoskeletal Injuries	3
The Outcome of Intra-Articular Distal Radius Fractures Treated With Fragment-Specific Fixation	3
Slip-related muscle activation patterns in the stance leg during walking	2
Video motion analysis for the synthesis of dynamic cues and Futurist art	3
Examination of extrinsic foot muscles during running using mfMRI and EMG	3
Effect of High-Intensity Strength-Training on Functional Measures of Balance Ability in Balance-Impaired Older Adults	3
A comparison of dynamic coronal plane excursion between matched male and female athletes when performing single leg landings	3
La distraction physaire dans les inégalités de longueur et déviations angulaires des membres: Physeal distraction for limb length discrepancy and angular deformity	3
Wartenberg's pendulum, repose and the 'gripped' patella—part 1: quadriceps normotonia	3
Aging with a disability	3
Age and walking speed effects on muscle recruitment in gait termination	3
Gait termination in young and older adults: effects of stopping stimulus probability and stimulus delay	3
Muscle activation patterns of selected lower extremity muscles during stepping and cutting tasks	3
Associations of knee angles, moments and function among subjects that are healthy and anterior cruciate ligament deficient (ACLD) during straight ahead and crossover cutting activities	3
Work-related Strain Injuries in Physiotherapists: Prevalence and prevention of musculoskeletal disorders	3
Increasing exercise tolerance of persons limited by claudication pain using polestriding	3
Botulinum Neurotoxin Intramuscular Chemodenervation: Role in the Management of Spastic Hypertonia and Related Motor Disorders	3
Changes in muscle moment arms following split tendon transfer of tibialis anterior and tibialis posterior	3
Nonlinear analysis of orthostatic posture in patients with vertigo or balance disorders	3
Increased variability of continuous overground walking in neuropathic patients is only indirectly related to sensory loss	3
The importance of pediatric injury prevention to trauma care	3
Assessment of neuromuscular response characteristics at the knee following a functional perturbation	3
Highlights of the Fifteenth Annual Summer Meeting of the American Orthopaedic Foot and Ankle Society, Fajardo, Puerto Rico, 9–11 July 1999	3
Biomechanics: An integral part of sport science and sport medicine	3
The local management of soft tissue sarcoma	3
Patellar contact forces with and without patellar resurfacing in total knee arthroplasty	3
Movement-induced modulation of soleus H reflexes with altered length of biarticular muscles	3
The Influence of Tobacco Use on Endosseous Implant Failures	3
Antiplatelet therapy in atherosclerotic cardiovascular disease	3
SENSORI-SENSORY AFFERENT CONDITIONING WITH LEG MOVEMENT: GAIN CONTROL IN SPINAL REFLEX AND ASCENDING PATHS	3
Moderate peripheral neuropathy impairs weight transfer and unipedal balance in the elderly	3
SPINA BIFIDA	3
Relationship between foot flexibility and urinary incontinence in nulliparous varsity athletes	3
The Potential for Adverse Reactions Due to the Presence of Additives and Preservatives in Intravenous Solutions and Medications	3
Effect of tibial component position on patellar strain following total knee arthroplasty	3
The early history of arthroplasty of the wrist From amputation to total wrist implant	3
Tibial translation and hamstring activity during active and passive arthrometric assessment of knee laxity	3
Effects of patella alta and patella infera on patellofemoral contact forces	3
RECENT ADVANCES IN MAGNETIC RESONANCE IMAGING OF THE MUSCULOSKELETAL SYSTEM	3
Rehabilitation of the Physically Challenged Athlete	3
Changes in early 'automatic' postural responses associated with the prior-planning and execution of a compensatory step	3
Do postural responses to transient and continuous perturbations show similar vision and amplitude dependence?	3
Total Knee Arthroplasty: Indications, Preparation, Procedure	3
NEUROGENIC FACTORS IN THE ETIOPATHOGENESIS OF OSTEOARTHRITIS	3
Treatment of congenital and acquired hemophilia patients by extracorporeal removal of antibodies to coagulation factors: A review of US clinical studies 1987–1990	3
Spinal Cord Injury	3
Treatment of Malunions and Mal-Nonunions of the Femur and Tibia by Detailed Preoperative Planning and the Ilizarov Techniques	3
Soft tissue sarcomas	3
Aging of human segmental oligosynaptic reflexes for control of leg movement	3
Pathologic Anatomy of Hallux Abducto Valgus	3
Impact of drug screening in suspected overdose	3
Learning and locomotor reaction times	3
Stanozolol in postmenopausal osteoporosis: Therapeutic efficacy and possible mechanisms of action	3
8: The Antibiotic Therapy of Septic Arthritis	3
Altered movement dynamics in soldiers undergoing multiple bouts of load carriage	3
Applicability of the Madymo Pedestrian Model for forensic fall analysis	3
Naviculocuneiform joint arthrodesis in a cohort of 36 patients	3
Does an acute transition to different footwear conditions affect walking patterns in people with different experiences of minimalist footwear?	3
Obese adolescents have higher risk for femur fracture after motor vehicle collision	3
The relationship between executed cut angle and speed with lower extremity joint angles during unanticipated side-step cutting in soccer players	2
Think fast, stay healthy? A narrative review of neurocognitive performance and lower extremity injury	7
The effects of subsensory electrical noise stimulation on the reactive control of balance during support surface perturbations	3
Quantifying the difference between male and female agility in football players: A cross-sectional study	3
Lower extremity osteotomies for limb preservation: Indications, outcomes, and risk factors	3

	Comparison of elective implant removal and complication rates between mini and small fragment implants for lateral malleolar fixation	3
	Effect of joint angle positioning on shearwave speed and variability with ultrasound shearwave elastography in asymptomatic Achilles and patellar tendons	3
	Modified Lapidus procedure with a nitinol staple and two screw construct technique	3
	Adding secondary cognitive tasks to drop vertical jumps alters the landing mechanics of athletes with anterior cruciate ligament reconstruction	2
	Anticipation augments distal leg muscle neuromechanics before, during, and after treadmill-induced perturbations applied during walking	3
	Effect of the cutting angle on intra-foot coordination pattern during unanticipated side-cutting maneuvers in female soccer players	5
	Perceived barriers and facilitators to exercise adherence in osteoarthritis: A thematic synthesis of qualitative studies	3
	Proactive modifications to walking stability under the threat of large, anterior or posterior perturbations	3
	Relationship of knee abduction moment to lower extremity segment accelerations during sport-specific movements in youth anterior cruciate ligament reconstruction patients at return-to-play	3
	Effects of somatosensory-stimulating foot orthoses on postural balance in older adults: A computerized dynamic posturography analysis	3
	Time-frequency analysis of muscle activation patterns in individuals with chronic ankle instability during walking	3
**google scholar**	Shoe collar height effect on athletic performance, ankle joint kinematics and kinetics during unanticipated maximum-effort side-cutting performance	2
	Gender differences in the kinematics of unanticipated cutting in young athletes	2
	Ankle anticipatory postural adjustments during gait initiation in healthy and post-stroke subjects	2
	Unanticipated ankle inversions are significantly different from anticipated ankle inversions during drop landings: overcoming anticipation bias	2
	The influence of ankle dorsiflexion range of motion on unanticipated cutting kinematics	5
	Stepping with an ankle foot orthosis re-examined: a mechanical perspective for clinical decision making	2
	Effect of ankle taping on knee and ankle joint biomechanics in sporting tasks	2
	Muscle force contributions to ankle joint contact forces during an unanticipated cutting task in people with chronic ankle instability	2
	Effects of anticipation on joint kinematics during inversion perturbation in individuals with chronic ankle instability	2
	Copers adopt an altered dynamic postural control compared to individuals with chronic ankle instability and controls in unanticipated single-leg landing	2
	Anticipating ankle inversion perturbations during a single-leg drop landing alters ankle joint and impact kinetics	2
	Type of unanticipated stimulus affects lower extremity kinematics and kinetics during sidestepping	2
	Biomechanics of ankle giving way: A case report of accidental ankle giving way during the drop landing test	3
	Preparation time influences ankle and knee joint control during dynamic change of direction movements	2
	The role of anticipatory postural adjustments and gravity in gait initiation	2
	Effects of ankle bracing on knee joint biomechanics during an unanticipated cutting maneuver	3
	Impact of ankle muscle fatigue and recovery on the anticipatory postural adjustments to externally initiated perturbations in dynamic postural control	2
	Arch-support induced changes in foot-ankle coordination in young males with flatfoot during unplanned gait termination	2
	Biomechanical analysis and inertial sensing of ankle joint while stepping on an unanticipated bump	3
	Lower limb muscle activity and kinematics of an unanticipated cutting manoeuvre: a gender comparison	2
	Modulation of anticipatory postural adjustments using a powered ankle orthosis in people with Parkinson's disease and freezing of gait	2
	Joint dynamics of rear-and fore-foot unplanned sidestepping	5
	Effects of mental fatigue on biomechanical characteristics of lower extremities in patients with functional ankle instability during unanticipated side-step cutting	2
	Lower extremity joint kinematics of a simulated lateral ankle sprain after drop landings in participants with chronic ankle instability	2
	Gait termination strategies differ between those with and without ankle instability	2
	Changes in lower limb kinematics, kinetics, and muscle activity in subjects with functional instability of the ankle joint during a single leg drop jump	2
	The effect of ankle taping and balance exercises on postural stability indices in healthy women	3
	Investigating the anticipatory postural adjustment phase of gait initiation in different directions in chronic ankle instability patients	2
	Anticipatory coadaptation of ankle stiffness and sensorimotor gain for standing balance	2
	The role of anticipatory postural adjustments in compensatory control of posture: 2. Biomechanical analysis	3
	Influence of Landing in Neuromuscular Control and Ground Reaction Force with Ankle Instability: A Narrative Review	2
	Effect of anticipation on lower extremity biomechanics during side-and cross-cutting maneuvers in young soccer players	2
	Kinematics and muscle activities of the lower limb during a side-cutting task in subjects with chronic ankle instability	3
	Lower-extremity kinematics during ankle inversion perturbations: a novel experimental protocol that simulates an unexpected lateral ankle sprain mechanism	2
	Anticipatory control of center of mass and joint stability during voluntary arm movement from a standing posture: interplay between active and passive control	2
	Anticipatory effects on lower extremity neuromechanics during a cutting task	2
	Altered postural control in anticipation of postural instability in persons with recurrent low back pain	2
	The Effect of Arch Stiffness on the Foot–Ankle Temporal Kinematics during Gait Termination: A Statistical Nonparametric Mapping Study	3
	Anticipatory effects on anterior cruciate ligament loading during sidestep cutting	2
	External ankle taping does not alter lower extremity side-step cut and straight sprint biomechanics in young adult males	2
	Do fast voluntary movements necessitate anticipatory postural adjustments even if equilibrium is unstable?	2
	Effects of decision making on landing mechanics as a function of task and sex	2
	Neuromuscular control in individuals with chronic ankle instability: A comparison of unexpected and expected ankle inversion perturbations during a single leg drop …	3
	Biomechanical approach to quantifying anticipatory postural adjustments in the elderly	3
	Ankle and midfoot kinetics during normal gait: a multi-segment approach	2
	Anticipatory kinematics and muscle activity preceding transitions from level-ground walking to stair ascent and descent	3
	Anticipatory locomotor adjustments of the trail limb during surface accommodation	2
	Soldier-relevant loads impact lower limb biomechanics during anticipated and unanticipated single-leg cutting movements	2
	The effects of an unanticipated side-cut on lower extremity kinematics and ground reaction forces during a drop landing	2
	The coordination patterns of the foot segments in relation to lateral ankle sprain injury mechanism during unanticipated changes of direction	2
	Ankle muscle stiffness alone cannot stabilize balance during quiet standing	2
	Mechanical work performed by distal foot-ankle and proximal knee-hip segments during anticipated and unanticipated cutting	2
	The sway-density curve and the underlying postural stabilization process	2
	Center of pressure excursion and muscle activation during gait initiation in individuals with and without chronic ankle instability	2
	Does wearing a prophylactic ankle brace during drop landings affect lower extremity kinematics and ground reaction forces?	3
	Combined effects of fatigue and decision making on female lower limb landing postures: central and peripheral contributions to ACL injury risk	2
	Foot kinematics and kinetics during adolescent gait	2
	Asymmetries in reactive and anticipatory balance control are of similar magnitude in Parkinson's disease patients	2
	A biomechanical comparison of single-leg landing and unplanned sidestepping	2
	Sex-specific landing biomechanics and energy absorption during unanticipated single-leg drop-jumps in adolescents: implications for knee injury mechanics	2
	A comprehensive evaluation of the variation in ankle function during gait in children and youth with Charcot–Marie–Tooth disease	3
	Footwear-induced changes in ankle biomechanics during unanticipated side-step cutting in female soccer players	5
	Gait termination control strategies are altered in chronic ankle instability subjects	2
	Ankle kinematics, center of pressure progression, and lower extremity muscle activity during a side-cutting task in participants with and without chronic ankle instability	2
	Individuals with chronic ankle instability exhibit decreased postural sway while kicking in a single-leg stance	2
	The role of anticipatory postural adjustments during whole body forward reaching movements	3
	Reaching-lifting-placing task during standing after stroke: coordination among ground forces, ankle muscle activity, and hand movement	3
	Kinematics and kinetics of normal and planovalgus feet during walking	3
	Visualisation to enhance biomechanical tuning of ankle-foot orthoses (AFOs) in stroke: study protocol for a randomised controlled trial	2
	Biomechanically based clinical decision making in pediatric foot and ankle surgery	3
	Do people with Parkinson's disease change strategy during unplanned gait termination?	3
	Expecting ankle tilts and wearing an ankle brace influence joint control in an imitated ankle sprain mechanism during walking	2
	Normative data for passive ankle plantarflexion–dorsiflexion flexibility	3
	Importance of body sway velocity information in controlling ankle extensor activities during quiet stance	2
	Weight-bearing dorsiflexion range of motion and landing biomechanics in individuals with chronic ankle instability	3
	Biomechanical but not timed performance asymmetries persist between limbs 9 months after ACL reconstruction during planned and unplanned change of direction	2
		1
	Proactive and reactive neuromuscular control in subjects with chronic ankle instability: evidence from a pilot study on landing	2
	The effect of external ankle support on the kinematics and kinetics of the lower limb during a side step cutting task in netballers	3
	Criteria-based return to sport decision-making following lateral ankle sprain injury: a systematic review and narrative synthesis	3
	The Biomechanics of the Foot and Ankle	3
	The mechanical consequences of dynamic frontal plane limb alignment for non-contact ACL injury	2
	How to sprain your ankle–a biomechanical case report of an inversion trauma	3
	Joint kinetics: methods, interpretation and treatment decision-making in children with cerebral palsy and myelomeningocele	3
	The relationship between the sensory responses to ankle-joint loading and corticomotor excitability	3
	Test-retest reliability and minimal detectable change of ankle kinematics and spatiotemporal parameters in MS population	2
	Preparatory co-activation of the ankle muscles may prevent ankle inversion injuries	2
	Time-frequency analysis of muscle activation patterns in people with chronic ankle instability during Landing and cutting tasks	2
	Video analysis of anterior cruciate ligament injury: abnormalities in hip and ankle kinematics	3

Muscle Synergies in People With Chronic Ankle Instability During Anticipated and Unanticipated Landing-Cutting Tasks	2
Chronic ankle instability: diagnosis and treatment	3
Effects of a dynamic core stability program on the biomechanics of cutting maneuvers: A randomized controlled trial	9
The effect of high intensity exercise and anticipation on trunk and lower limb biomechanics during a crossover cutting manoeuvre	2
Elderly adults delay proprioceptive reweighting during the anticipation of collision avoidance when standing	3
Closing the wearable gap—part III: use of stretch sensors in detecting ankle joint kinematics during unexpected and expected slip and trip perturbations	3
Head-trunk movement coordination in the standing posture	3
Changes in gait when anticipating slippery floors	3
Altered neuromuscular control and ankle joint kinematics during walking in subjects with functional instability of the ankle joint	3
Three-dimensional kinematics of the knee and ankle joints for three consecutive push-offs during ice hockey skating starts	3
Foot and ankle kinematics during descent from varying step heights	3
Effects of ankle Kinesio taping on knee and ankle joint biomechanics during unanticipated jumps in collegiate athletes	3
The effect of ankle supports on lower limb biomechanics during functional tasks: a systematic review with meta-analysis	3
The effect of choice reaction time task on pre-landing muscle timing in athletes with and without chronic ankle instability	2
Dual-task and anticipation impact lower limb biomechanics during a single-leg cut with body borne load	3
Foot and ankle kinematics and ground reaction forces during ambulation	3
The effects of anticipation on the mechanics of the knee during singleg cutting tasks: A systematic review	7
Anticipation of landing leg masks ankle inversion orientation deficits and peroneal insufficiency during jump landing in people with chronic ankle instability	2
Effect of external ankle support on ankle and knee biomechanics during the cutting maneuver in basketball players	3
Distinct Motion Control Strategy during Unanticipated Landing: Transitioning from Copers to Chronic Ankle Instability	5
Flexibility of anticipatory postural adjustments revealed by self-paced and reaction-time arm movements	3
Biomechanical study of the programming of anticipatory postural adjustments associated with voluntary movement	3
Anticipatory postural adjustments during cutting manoeuvres in football and their consequences for knee injury risk	4
Jump landing biomechanics during a laboratory recorded recurrent ankle sprain	3
Effect of Chronic Ankle Instability on the Biomechanical Organization of Gait Initiation: A Systematic Review	2
The ankle dorsiflexion kinetics demand to increase swing phase foot-ground clearance: implications for assistive device design and energy demands	3
Expecting ankle tilts and wearing an ankle brace influence joint control in an imitated ankle sprain mechanism during walking	2
Influence of ankle loading on the relationship between temporal pressure and motor coordination during a whole-body paired task	2
The effects of fatigue and anticipation on the mechanics of the knee during cutting in female athletes	4
Decreased proprioception is associated with inferior postural control during unplanned landing in individuals with chronic ankle instability	2
Coordination and variability during anticipated and unanticipated sidestepping	2
Preparatory co-activation of the ankle muscles may prevent ankle inversion injuries	2
Modulation of anticipatory postural adjustments using a powered ankle orthosis in people with Parkinson’s disease and freezing of gait	3
Anticipatory postural adjustments during standing in below-the-knee amputees	3
Medial foot loading on ankle and knee biomechanics	3
Effect of age on anticipatory postural adjustments in unilateral arm movement	2
Decision making and experience level influence frontal plane knee joint biomechanics during a cutting maneuver	2
Neuromuscular control in individuals with chronic ankle instability: a comparison of unexpected and expected ankle inversion perturbations during a single	3
Ankle and midfoot kinetics during normal gait: a multi-segment approach	3
Effects of ankle dorsiflexion training on anticipatory postural adjustments during gait initiation in patients with Parkinson's disease	2
Effects of stroboscopic proprioception training on unplanned landing deficits in chronic ankle instability	7
Ground reaction force data in functional ankle instability during two cutting movements	3
Does chronic ankle instability affect side-cutting in female soccer players?	2
Changes in the range of angular variation of the ankle, knee, hip and neck joints related to the awareness of an impending perturbation	2
The influence of asymptomatic hypermobility on unanticipated cutting biomechanics	2
Evaluation of the interaction between contact force and decision making on lower extremity biomechanics during a side-cutting maneuver	2
The effect of changes in the body configuration on anticipatory postural adjustments	3
Investigation of the effects of high-intensity, intermittent exercise and unanticipation on trunk and lower limb biomechanics during a side-cutting maneuver using	2
Ankle joint control in people with chronic ankle instability during run-and-cut movements	2
The effect of dual tasking on foot kinematics in people with functional ankle instability	2
External postural perturbations induce multiple anticipatory postural adjustments when subjects cannot pre-select their stepping foot	3
Trunk, pelvis and lower limb coordination between anticipated and unanticipated sidestep cutting in females	2
Anticipatory postural control strategies related to predictive perturbations	3
Assessment of Muscle Synergies in Chronic Ankle Instability Patients During Unanticipated and Anticipated Landing	6
Ankle bracing, plantar-flexion angle, and ankle muscle latencies during inversion stress in healthy participants	2
Proportional myoelectric control of a powered ankle prosthesis for postural control under expected perturbation: A pilot study	2
Application of multi-criteria decision-making methods in the selection of additive manufacturing materials for solid ankle foot orthoses	3
Peak forces and force generating capacities of lower extremity muscles during dynamic tasks in people with and without chronic ankle instability	2
FOOT SEGMENTS DURING UNANTICIPATED CHANGES OF DIRECTION: THEORETICAL IMPLICATIONS FOR PROPHYLACTIC LATERAL ANKLE SPRAIN …	5
Effects Of Chronic Ankle Instability On Landing Kinematics During Unanticipated Single-leg Drop Landing	3
A survey of clinical practice patterns of physical therapists for the use of ankle-foot orthoses or functional electrical stimulation poststroke	3
Analysing lower limb motion and muscle activation in athletes with ankle instability during dual-task drop-jump	3
Effects of Elastic Ankle Braces and Anticipation Conditions on Lower Limb Biomechanics During Badminton Lunge Movements	7
Sex and limb differences in hip and knee kinematics and kinetics during anticipated and unanticipated jump landings: implications for anterior cruciate ligament injury	4
Anticipatory postural adjustments in a bimanual, whole body lifting task with an object of known weight	3
Effect of unpredictable timing on the hip, knee, and ankle kinematics and center of mass during deceleration tasks.	2
Anticipatory muscle responses in transitions from rigid to compliant surfaces: towards smart ankle-foot prostheses	3
Peak lower extremity landing kinematics in dancers and nondancers	3
Copers exhibit altered ankle and trunk kinematics compared to the individuals with chronic ankle instability during single-leg landing	8
Hip, knee, and ankle kinematics of high range of motion activities of daily living	3
A comparative biomechanical analysis during planned and unplanned gait termination in individuals with different arch stiffnesses	3
EFFECTS OF ANKLE IMMOBILIZATION ON KNEE JOINT BIOMECHANICS DURING AN UNANTICIPATED CUTTING MANEUVER	2
Individuals with chronic ankle instability exhibit altered ankle kinematics and neuromuscular control compared to copers during inversion single-leg landing	2
Effects of Combining Transcranial Direct Current Stimulation With Balance Training on Anticipatory Postural Adjustments in Persons With Chronic Ankle Instability	3
Variability of anticipatory postural adjustments during gait initiation in individuals with Parkinson disease	3
Both anticipatory and compensatory postural adjustments are adapted while catching a ball in unstable standing posture	3
Reduced hamstring strength increases anterior cruciate ligament loading during anticipated sidestep cutting	3
The influence of prophylactic ankle bracing on knee joint moments and ground reaction force during an unanticipated 90° side-step cutting	2
Voluntary toe-walking gait initiation: electromyographical and biomechanical aspects	3
Evaluation of assumptions in foot and ankle biomechanical models	3
Strain in the tibial and plantar nerves with foot and ankle movements and the influence of adjacent joint positions	3
Kinematics analysis of ankle inversion ligamentous sprain injuries in sports: five cases from televised tennis competitions	2
A coactivation strategy in anticipatory postural adjustments in persons with Down syndrome	3
The foot and ankle in cerebral palsy	2
The effect of interventions anticipated to improve plantar intrinsic foot muscle strength on fall-related dynamic function in adults: a systematic review	3
Comparative gait initiation kinematics between simulated unilateral and bilateral ankle hypomobility: does bilateral constraint improve speed performance?	3
Effects of repetitive lifting on kinematics: inadequate anticipatory control or adaptive changes?	3
Coordination of rapid stepping with arm pointing: anticipatory changes and step adaptation	2
The alteration of neuromuscular control strategies during gait initiation in individuals with chronic ankle instability	2
Differences in lateral drop jumps from an unknown height among individuals with functional ankle instability	2
A comparison of subtalar joint motion during anticipated medial cutting turns and level walking using a multi-segment foot model	2
Lower limb joint motion during a cross cutting movement differs in individuals with and without chronic ankle instability	2
The effect of asymmetry of posture on anticipatory postural adjustments	3
Normal function of the ankle and foot: biomechanics and quantitative analysis	3
Simulated ankle equinus affects knee kinematics during gait	3
Direct measurement of ankle stiffness during quiet standing: implications for control modelling and clinical application	3
Directional specificity of postural muscles in feed-forward postural reactions during fast voluntary arm movements	2
Ankle biomechanics of the three-step layup in a basketball player with chronic ankle instability	3
The role of military footwear and workload on ground reaction forces during a simulated lateral ankle sprain mechanism	3
Ankle Rotation and Muscle Loading Effects on the Calcaneal Tendon Moment Arm: An In Vivo Imaging and Modeling Study	3
Prophylactic ankle braces and knee varus-valgus and internal-external rotation torque	3

	Decoupling of laxity and cortical activation in functionally unstable ankles during joint loading	3
	Effect of shoe wearing time and midsole hardness on ground reaction forces, ankle stability and perceived comfort in basketball landing	3
	Evaluating anticipatory control strategies for their capability to cope with step-down perturbations in computer simulations of human wal	3
	P1 Kinematic difference during anticipated and unanticipated cut following drop landings in individuals with chronic ankle instability and healthy cor	2
	Ankle and subtalar kinematics during dorsiflexion-plantarflexion activiti	3
	Muscle activation during landing before and after fatigue in individuals with or without chronic ankle instat	2
	Postural threat influences the coupling between anticipatory and compensatory postural adjustments in response to an external perturb	3
	Comparison of skating kinetics and kinematics on ice and on a synthetic surfa	2
	Assessment Algorithm and Decision Makin	3
	Ankle strategies for step-aside movement during straight walkir	2
	Individuals with chronic ankle instability compensate for their ankle deficits using proximal musculature to maintain reduced postural sway while kicking	3
	Anticipatory control related to the upward propulsive force during the rising on tiptoe from an upright standing posi	2
	Hip-abductor fatigue influences sagittal plane ankle kinematics and shank muscle activity during a single-leg forward ju	3
	Ankle arthroplasty with preoperative coronal plane deformity: short-term resu	3
	Effects of midsole thickness on ground reaction force, ankle stability, and sports performances in four basketball moveme	3
	Improving independence in the community for stroke survivors: The role of biomechanics visualisation in ankle-foot orthosis tu	3
	A model of the neuro-musculo-skeletal system for anticipatory adjustment of human locomotion during obstacle avoida	3
	Soft tissue sarcomas of the foot and ankle: impact of unplanned excision, limb salvage, and multimodality ther	3
	Effects of preparatory period on anticipatory postural control and contingent negative variation associated with rapid arm movement in standing pos	3
	Analysis of ankle kinetics during walking in individuals with Down syndror	3
	Evaluation of a powered ankle-foot prosthetic system during walki	7
	Mechanical work performed by distal foot-ankle and proximal knee-hip segments during anticipated and unanticipated cut	3
	Neuromuscular fatigue and cognitive constraints independently modify lower extremity landing biomechanics in healthy and chronic ankle instability indivi	3
	Robotically quantifying finger and ankle proprioception: Role of range, speed, anticipatory errors, and learn	3
	Which functional tests and self-reported questionnaires can help clinicians make valid return to sport decisions in patients with chronic ankle instab	3
	Comparison of in vivo ankle joint kinematics after total ankle replacement with different types of talar compone	3
	Altered biomechanics in individuals with chronic ankle instability compared with copers and controls during g	2
	Muscle contributions to reduced ankle joint contact force during drop vertical jumps in patients with chronic ankle instal	2
	The effect of sex, skill level and a defender on cutting kinematics in soccer play	3
	Ankle Kinematics Characterization in Children with Idiopathic Toe Walking: Does the Foot Model Change the Clinical Evaluati	2
	Are landing biomechanics altered in elite athletes with chronic ankle instabil	2
	Changes in postural control after a ball-kicking balance exercise in individuals with chronic ankle instab	3
	Within-socket myoelectric prediction of continuous ankle kinematics for control of a powered transtibial prosthe	3
	Return-to-play decision-making following ankle injury: a comprehensive case analysis of the functional hop	3
	Alterations in gait initiation are present in those with posttraumatic ankle osteoarthritis: a pilot stu	3
	Inversion injury biomechanics in functional ankle instability: a cadaver study of simulated	3
	Comparative effects of different manual techniques on electromyography activity, kinematics, and muscle force in limited ankle dorsiflexion synd	3
	The effect of various footwear types and materials, and of fixing of the ankles by footwear, on upright posture co	3
	Ankle bracing effects on knee and hip mechanics during landing on inclined surfac	2
	Anticipatory postural adjustments associated with lateral and rotational perturbations during stand	2
	Effects of Differing Platform Orientations on Forefoot and Hindfoot Kinematics in Chronic Ankle Instability During Single Leg Lan	3
	Effect of the sagittal ankle angle at initial contact on energy dissipation in the lower extremity joints during a single-leg lan	3
	Quantitative segmental analysis of weight-bearing radiographs of the foot and ankle for children: normal align	3
	Unexpected inversion perturbation during a single-leg landing in patients with chronic ankle instab	3
	Effect of vibration-induced postural illusion on anticipatory postural adjustment of voluntary arm movement in standing hur	2
	Neuromuscular control and ankle instabili	2
	Reactive and anticipatory control of posture and bipedal locomotion in a nonhuman prim	3
	Effects of External Ankle Taping on Lower Extremity Kinetics and Kinematics in Young Adult Ma	2
	Anticipatory locomotor control for obstacle avoidance in mid-childhood aged childr	2
	Gait pattern classification in children with Charcot–Marie–Tooth disease type 1	3
	The Effect of Different Degrees of Ankle Dorsiflexion Restriction on the Biomechanics of the Lower Extremity in S-Jumping	3
	Total ankle arthroplasty: Strength, pain, and moti	3
	Anticipatory locomotor adjustments for avoiding visible, fixed obstacles of varying proxim	3
	Predicting biological joint moment during multiple ambulation tas	2
	Comparison of Anticipatory and Compensatory Postural Adjustment Timing Between Individuals with Chronic Ankle Instability and Healthy Controls Durin	3
	Analysis of stress response distribution in patients with lateral ankle ligament injuries: A study of neural control strategies utilizing predictive computing m	3
	Neuromuscular and lower limb biomechanical differences exist between male and female elite adolescent soccer players during an unanticipated side-cut mane	3
	Closed-Loop Reflex Responses of the Lateral Ankle Musculature From Various Thresholds During a Lateral Ankle Sprain Perturba	3
	Ankle arthrodesis versus total ankle replacement: how do I decid	3
	Exploring the relationship between the supination resistance test and the effects of foot orthoses on the foot and ankle biomechanics during wa	3
	The effects of predisposition and direction on ankle sprain risk predictive factors during jump landi	3
	Effects of movement direction and limb dominance on ankle muscular force in sidestep cutt	3
	of a novel limb symmetry index to discriminate movement strategies during bilateral jump landing in individuals with ACLR and with and without a history of an	3
	Alterations in surgical decision making in patients with cerebral palsy based on three-dimensional gait analy	3
	Comparing virtual reality and balance training effects on postural strategies during ball kicking in soccer players with chronic ankle instal	3
	Total ankle replacement: the results in 200 ankl	3
	Modifications in ankle dorsiflexor activation by applying a torque perturbation during walking in persons post-stroke: a case se	3
	Physically active older adults display alterations in gait initiati	3
	Variations of tool and task characteristics reveal that tool-use postures are anticipat	3
	Prelanding movement strategies among chronic ankle instability, coper, and control subj	2
	Control of stair ascent and descent with a powered transfemoral prosthes	3
	Are biomechanical stability deficits during unplanned single-leg landings related to specific markers of cognitive functi	2
	Design of an Electromechanical Ankle-Foot Orthosis with Controlled Locking and Dorsiflexion Ass	3
	Effect of a combined inversion and plantarflexion surface on ankle kinematics and EMG activities in land	3
	Biomechanics of the normal and arthritic ankle joi	3
	Does flip-flop style footwear modify ankle biomechanics and foot loading pattern	3
	Anticipatory postural adjustment during gait initiation in multiple sclerosis patients: A systematic revi	3
	Biomechanical and neuromuscular characteristics of male athletes: implications for the development of anterior cruciate ligament injury prevention prog	3
	Comparing lab and field agility kinematics in young talented female football players: Implications for ACL injury preven	3
	Kinematic behavior of the ankle following malleolar fracture repair in a high-fidelity cadaver mo	2
	Validation of the Ottawa Ankle Rules for acute foot and ankle injuri	3
	Effects of a semi-rigid ankle brace on ankle joint loading during landing on inclined surfac	3
	Ankle Dorsiflexion displacement is associated with hip and knee kinematics in females following anterior cruciate ligament reconstruc	3
	Mechanical demand and multijoint control during landing depend on orientation of the body segments relative to the reaction f	3
	Sensitivity of the OLGA and VCM models to erroneous marker placement: Effects on 3D-gait kinemat	3
	People with recurrent ankle sprains do not change their ankle strategy in anticipation of a perturbation e	3
	Biomechanics of supination ankle sprain: a case report of an accidental injury event in the laborat	3
	Directional deficits in reactive postural control during perturbations among groups of chronic ankle instability, ankle sprain coper, and healthy cc	3
	The influence of circadian variation on etiological markers of ankle inj	3
	Effect of Tilted surfaces on Ankle Kinematics and EMG activities in landi	3
	Stimulus prediction and postural reaction: phase-specific modulation of soleus H-reflexes is related to changes in joint kinematics and segmental strategy in pertu	3
	Biomechanical effects of an injury prevention program in preadolescent female soccer athle	2
	Anticipatory Muscle Responses for Transitioning Between Rigid Surface and Surfaces of Different Compliance: Towards Smart Ankle-foot Prosthe	3
	Slip-related muscle activation patterns in the stance leg during walki	2
	Dynamic finite element analyses to compare the influences of customised total talar replacement and total ankle arthroplasty on foot biomechanics durin	3
	Biomechanics of the Ankle: Exploring Structure, Function, and Injury Mechanis	3
	Prophylactic ankle taping: influence on treadmill-running kinematics and running econ	3
	Neuromuscular risk factors for knee and ankle ligament injuries in male youth soccer play	2
	Preparatory process for anticipatory postural adjustments: modulation of leg muscles reflex pathways during preparation for arm movements in standing	3
	Differences in lateral drop-jumps from an unknown height among individuals with ankle instab	3
	Efficacy of shoes and boots in preventing motorcycle-related ankle inversi	3
	The effect of tape on ankle joint landing kinematics in subjects with chronic ankle instab	3
	Unplanned surgical excision of tumors of the foot and ank	3
	Visualization and in situ analysis of leukocyte trafficking into the ankle joint in a systemic murine model of rheumatoid art	2
	Adaptation of postural control to weightlessnes	3
	Frequency of Foot and Ankle Injuries in Professional Soccer Players Following the Introduction of Prehab that Incorporates Elements of the FIF	3
	Clinical validation of a system for the analysis of pediatric foot and ankle kinematics during g	3
	Postural Control During Sudden, Unanticipated Perturbations with and without Visual Disturbance Among Groups of Chronic Ankle Instability, Coper, and He	3
	The role of anticipatory postural adjustments in compensatory control of posture: 1. Electromyographic anal	3
	THE EFFECT OF ANKLE TAPING ON ANKLE AND KNEE JOINT BIOMECHANICS IN SPORTING TAS	2
**Reference lists**	The influence of prophylactic ankle bracing on knee joint moments and ground reaction force during an unanticipated 90° side-step cutting	5
	EFFECTS OF ANKLE IMMOBILIZATION ON KNEE JOINT BIOMECHANICS DURING AN UNANTICIPATED CUTTING MANEUVER	5
	Individuals with chronic ankle instability exhibit altered ankle kinematics and neuromuscular control compared to copers during inversion single-leg landing	3
	Lower limb joint motion during a cross cutting movement differs in individuals with and without chronic ankle instability	2
	Ankle joint control in people with chronic ankle instability during run-and-cut movements	5
	Kinematic difference during anticipated and unanticipated cut following drop landings in individuals with chronic ankle instability and healthy controls	7

Mechanical work performed by distal foot-ankle and proximal knee-hip segments during anticipated and unanticipated cutting Article type: Short …	2
Muscle activation during landing before and after fatigue in individuals with or without chronic ankle instability	3
Ankle dorsiflexion affects hip and knee biomechanics during landing	3
The effect of dual tasking on foot kinematics in people with functional ankle instability	3
Are landing biomechanics altered in elite athletes with chronic ankle instability	3
functional tests and self-reported questionnaires can help clinicians make valid return to sport decisions in patients with chronic ankle instability? A narrative …	3
Preparation time influences ankle joint control during dynamic change of direction movements	2
Neuromuscular and lower limb biomechanical differences exist between male and female elite adolescent soccer players during an unanticipated side-cut maneuver	5
Effects of External Ankle Taping on Lower Extremity Kinetics and Kinematics in Young Adult Males	2
FOOT SEGMENTS DURING UNANTICIPATED CHANGES OF DIRECTION: THEORETICAL IMPLICATIONS FOR PROPHYLACTIC LATERAL ANKLE SPRAIN	5
Criteria-based return to sport decision-making following lateral ankle sprain injury: a relevant part of the prevention–performance paradox for secondary and	3
Ankle bracing effects on knee and hip mechanics during landing on inclined surfaces	3
Are biomechanical stability deficits during unplanned single-leg landings related to specific markers of cognitive function?	4
The effect of choice reaction time task on pre-landing muscle timing in athletes with and without chronic ankle instability	2
Anticipation of landing leg masks ankle inversion orientation deficits and peroneal insufficiency during jump landing in people with chronic ankle instability.	2
Prelanding movement strategies among chronic ankle instability, coper, and control subjects	3
Coordination and variability during anticipated and unanticipated sidestepping	4
Decision Making and Experience Level Influence Frontal Plane Knee Joint Biomechanics During a Cutting Maneuver	4
	1
	1

**15**	**Included =1**
**180**	**duplicates= 2**
**948**	**wrong Topic: No Unanticipation-status or no decision-making = 3**
**15**	**no 3D ankle kinematic/kinetics = 4**
**39**	**no preplanned/anticipated control condition = 5**
**6**	**not healthy = 6**
**21**	**study design = 7**
**16**	**Others = 8**
**1**	**Relevant data not reported/provided = 9**
