# Supplementary material for: Effects of sport specific unplanned movements on ankle kinetics and kinematics in healthy athletes from systematic review with meta-analysis
Source: Sci Rep. 2025 Sep 12;15:32476. doi: 10.1038/s41598-025-18746-9 (PMC12432200; doi:10.1038/s41598-025-18746-9)
Supplement: Supplementary file 5 — Supplementary Information 5. [file 41598_2025_18746_MOESM5_ESM.pdf]

GRADE Criteria	Rating	Explanation
Study design	Low	Although, this study type is appropriate to investigate the research question of the present work, “low quality” evidence is attributed to this kind of study design.
Downgrading criteria		
Study limitations (Risk of Bias)	-1 level (all outcomes in the sagittal plane, except for plantarflexion angle)<br>-2 levels (all outcomes in the frontal and transverse plane)	Although overall study quality was rated good, potential risk of bias (mainly because potential confounding) cannot be excluded (outcomes in the sagittal plane).<br>Due to an even more pronounced risk of bias (inaccurate measures, e.g. lack of a biomechanical foot model), outcomes in the frontal and transverse plane were downgraded by 2 levels).<br>No clear indication of publication bias was observed for ankle plantarflexion angles. However, for all remaining outcomes, the risk of publication bias could not be assessed due to an insufficient number of studies and therefore cannot be ruled out, resulting in a downgrade of the certainty of evidence by one level.
Inconsistency	-1 level (for all outcomes except for ankle plantar- and dorsiflexion angles, which received no downgrade)	Effect sizes showed low to moderate variance, with heterogeneity across studies ranging from low to high (I^2 statistics). Confidence intervals were relatively wide, likely due to small sample sizes, though they largely overlapped, particularly for plantar- and dorsiflexion angles. For dorsiflexion angles, sensitivity analysis revealed low between-study heterogeneity. Within studies heterogeneity (more than one ES) was low (ω^2).
Uncertainty about directness	No downgrading	Rather high homogeneity of study population, conditions, and outcomes, direct comparisons available; certainty about directness rather high. However, the homogeneity of movement tasks between the included studies was rather low. The confounding influence of these factors on the effects found can therefore not be excluded
Imprecise or sparse data	-1 level (plantarflexion and inversion angle)<br>No downgrading for the remaining outcomes	For ankle plantarflexion and inversion angle the confidence intervals are rather wide, but mostly on the same side of effect (see forest plots); we believe that wide confidence intervals are the result of the small sample sizes and the greater range kinematic outcomes can reach compared to the subtler changes of kinetic outcomes. Nevertheless, this limits the precision of the estimated effect sizes.
Upgrading criteria	+1 level (a significant small effect size was found for both plantar-	No outcome received an upgrade for the potentially upgrading criteria: large magnitude of effect (significant small effect sizes or non-significant moderate effect

	and dorsiflexion angles, while a non-significant but medium effect size was observed for the external eversion moment, although this was primarily driven by outliers)	sizes), plausible residual confounding (control or exclusion for or of potential confounders), and dose-response gradient (not applicable)
**Overall grading**	- Low to moderate certainty of evidence for ankle plantarflexion angles)<br>- low certainty of evidence for ankle dorsiflexion angles<br>- Very low certainty of evidence for frontal and transverse ankle kinematics/kinetics	We are fairly confident that the observed effect estimates for ankle plantarflexion angles at initial contact closely reflect the true effect, and it is unlikely that future studies will substantially alter the magnitude or direction of these estimates. In contrast, for dorsiflexion angles, we are less confident in the observed effects, as they tend to be closer to null, and future research may meaningfully change both the size and direction of the estimates. For all other outcomes, there is very low certainty, and the true effects of unplanned movement remain highly uncertain.
