# Supplementary material for: Effects of sport specific unplanned movements on ankle kinetics and kinematics in healthy athletes from systematic review with meta-analysis
Source: Sci Rep. 2025 Sep 12;15:32476. doi: 10.1038/s41598-025-18746-9 (PMC12432200; doi:10.1038/s41598-025-18746-9)
Supplement: Supplementary file 6 — Supplementary Information 6. [file 41598_2025_18746_MOESM6_ESM.pdf]

R Foundation for Statistical Computing, Vienna, Austria, packages Meta and Robumeta (V 2.1)

```
# Open packages
> library(robumeta)
> library(meta)


# Open dataset
> datenblatt=read.csv2("C:\\...\\Datenblatt.csv") # Datenblatt = data sheet
> View(datenblatt)
```

Main effects of unplanned movement on each outcome (e.g. ankle plantarflexion etc.)

```
auswertung <- robu(Effekt ~ 1,
                var.eff.size = VE_2,
                data = subset(datenblatt, Outcome == X),
                studynum = Study,
                modelweights = "HIER",
                small = FALSE)
# auswertung = analysis/evaluation

# I² calculations
tau2 <- auswertung$tau.sq # Auswertung = analysis
v_bar <- mean(subset(datenblatt, Outcome == X)$VE_2)
I2 <- (tau2 / (tau2 + v_bar)) * 100
(-->v_bar = StErr^2)
print(I2)
```

etc.

Moderator/Subgroup analysis for significant outcomes

```
# Coding of moderator variables
datenblatt$Study=as.character(datenblatt$Study)
datenblatt$Sex=factor(datenblatt$Sex)
datenblatt$Expertise_level=factor(datenblatt$Expertise_level)
datenblatt$Run_landing=factor(datenblatt$Run_landing)
datenblatt$Stance_phase=factor(datenblatt$Stance_phase)

# Within subgroups:
auswertung = robu(Effekt ~ Sex-1, var.eff.size = VE_2, data=subset(datenblatt,(Outcome==X)),
studynum = Study,modelweights="HIER",small=FALSE)
print(auswertung)


# Between subgroups:
auswertung = robu(Effekt ~ Sex, var.eff.size = VE_2, data=subset(datenblatt,(Outcome==X)),
studynum = Study,modelweights="HIER",small=FALSE)
print(auswertung)
```

etc.

Forest Plots for each outcome (e.g. ankle plantarflexion etc.)

```
# Set dataframe
effekt <- 0.xxx      # pooled effect
ci_lower <- 0.xxx
ci_upper <- 0.xxx

# Create dataframe
df_summary <- data.frame(
Study = "Gepoolter Effekt (Outcome X)",
Effekt = effekt,
CI_lower = ci_lower,
CI_upper = ci_upper
)

# Set outcome
daten_outcomeX <- subset(datenblatt, Outcome == X)

# Create forest plot
forest_modell <- metagen(
TE = Effekt,
seTE = sqrt(VE),
studlab = Study,
data = daten_outcomeX,
sm = "SMD"
)
forest(forest_modell)

# robu-corrected values
forest(forest_modell)
forest_modell$TE.random <- 0.xxx
forest_modell$lower.random <- 0.xxx
forest_modell$upper.random <- 0.xxx
forest(forest_modell)

library(grid)

# Add p-value and I2
grid::grid.text("Gepoolter Effekt (robu): p = 0xxx, I² = xx%",
  x = 0.5, y = unit(1, "lines"), gp = grid::gpar(fontsize = 10))

etc.
```

Funnel Plot

```
auswertungfunnel=metagen(Effekt,sqrt(VE_2),byvar=Study,data=subset(datenblatt,Outcome==x))
funnel(auswertungfunnel)
metabias(auswertungfunnel)
```
